# Swingers in Germany: Sociodemography and Event Preferences Assessed from Harvested Web Data

**DOI:** 10.1007/s10508-025-03198-z

**Published:** 2025-08-01

**Authors:** Oliver Maor

**Affiliations:** FernUniversität in Hagen, Universitätsstraße 47, 58097 Hagen, Germany

**Keywords:** Sexuality, Consensual non-monogamy, Germany, Swingers, Data harvesting

## Abstract

This exploratory study investigated Germany’s heterosexual swinger community within the consensual non-monogamy spectrum. It used anonymized, verified data harvested in September and October 2023 from Joyclub, the leading online platform in Germany focused on sexual exploration. The first part examined anonymized profiles from self-declared swingers, totaling 22,973 individuals, revealing a mature demographic, with median ages of 44 for women and 46 for men. Despite high regional variations, no clear correlations between swinger prevalence and regional socioeconomic factors were found. Subgroup analysis identified BDSM-affiliated swingers and those involved in open relationships or polyamory, with a higher concentration in urban areas. The second study assessed 14,008 attendees at 82 categorized swinger events, which showed a preference for sex-focused events across all age strata. Contrary to the male-centric perspective, the data revealed events strongly focused on women’s preferences, including those targeting younger male partners. Events also combined sexual activities with social elements and drew participants from greater distances, indicating a strong commitment to the community lifestyle. A prominent presence of solo women was observed, contradicting earlier findings from other countries. Reduced pricing for women did not correlate with higher attendance, questioning the effectiveness of such strategies. The use of verified data and a large sample size provides robust insights into Germany’s swinger community, emphasizing gender participation and regional variations. This study offers an empirical foundation for future research and highlights the importance of local contexts and terminology in studying sexual behavior.

**Supplementary Information:**

The online version contains supplementary material available at 10.1007/s10508-025-03198-z.

## Introduction

This study aims to build on previous research by exploring the German swinger scene, which differs notably from other contexts in its openness and organization. Utilizing data from the prominent Joyclub (www.joyclub.de) website, this research seeks to provide updated insights into the age, gender, and social backgrounds of swingers by addressing gaps in current understanding. The specific characteristics of the Joyclub platform and the German context provide a novel opportunity for comprehensive data collection. The methodology applied in this study has both strengths and limitations, which will be discussed throughout the paper.

Swingers, as a subgroup of consensual non-monogamy (CNM), represent a unique dimension of intimate relationships. CNM broadly encompasses arrangements where individuals in a romantic relationship mutually consent to engage in sexual, romantic, or intimate relations with additional partners. This includes swinging, open relationships, and polyamory, as outlined by Balzarini and Muise (2020) and others.

Despite some media interest in consensual non-monogamy, limited quantitative data on the prevalence and acceptance of non-monogamous relationships are available for Germany. As Mayer (2024) highlighted, many studies focused on questions framed within a monogamy-centered perspective. This leaves gaps in understanding CNM practices because they are not adequately captured by traditional questions about extradyadic sexual encounters.

According to a study by Haversath et al. (2017), which did not distinguish between consensual and non-consensual non-monogamy, 7% of respondents (8% of men, 6% of women) reported engaging in sexual activity “outside their current relationship” (p. 548). The GeSiD study, conducted between 2018 and 2019, provided the first comprehensive data on sexual behavior in Germany (Matthiesen et al., 2021). In the course of that study, it was noted that 5% of women aged 18–35, 3% of women aged 36–75, and 9% of men across both age groups engaged in sexual activity outside their permanent relationship. Among them, 15% of women and 6% of men did so with their partner’s consent (Institut für Sexualforschung, Sexualmedizin und Forensische Psychiatrie, Universitätsklinikum Hamburg-Eppendorf [UKE], 2020, p. 28). This means that 0.6% of the heterosexual adults in Germany living in permanent partnerships engage in CNM. By contrast, North American CNM studies report prevalence rates of 4–9.7% for couples and 2.6–5.6% for the general adult population (Conley et al., 2013; Fairbrother et al., 2019; Levine et al., 2018; Rubel & Burleigh, 2020; Rubin et al., 2014).

Lehmiller (2020) found that, in the USA, 32.6% of respondents, all in monogamous relationships, imagined engaging in CNM. In Germany, a commercial survey by ElitePartner (PE Digital, 2023) reported lower figures, with 23% of men and 11% of women indicating they could imagine living in an open relationship. Lack of time for more than one partner, emotional concerns like potential jealousy, fear of being left alone by the partner, and the danger of falling in love were stated as frequent barriers. Religious concerns were not mentioned in the survey, aligning with Wolf’s (2008) finding that Germany is one of the most secular countries in the world. The ElitePartner survey should be interpreted with some caution, as it is not an academic study.

### Definition of Swinging

Swinging specifically involves couples who exchange partners temporarily, often at social events designed for this purpose (Balzarini & Muise, 2020; Wood et al., 2021). This practice is distinguished by its focus on sexual activities, rather than emotional or romantic involvement with external partners. Swingers typically maintain a primary emotional commitment to their partner while engaging in sexual activities with others, often in social settings (Wood et al., 2021).

Whereas earlier literature, such as Jenks (1998) and Vaillancourt (2006), focused predominantly on married couples engaging in swinging for sexual purposes, contemporary definitions have become more inclusive. Swinging is now seen as part of a broader spectrum of CNM relationships, where emotional and sexual exclusivity is not mandatory (Conley et al., 2018; Moors et al., 2013). This flexibility allows for overlapping identities and practices, such as “swolly” individuals who identify as both swingers and polyamorous (Moors et al., 2013).

Furthermore, swinging differs from polyamory, which emphasizes emotional and romantic commitments to multiple partners, and open relationships, which generally focus on sexual relations outside the primary couple with minimal emotional involvement (Balzarini et al., 2017; Baumgartner, 2020).

The swinging subculture itself is diverse, with varying degrees of involvement and practices among individuals and couples (Venn, 2015). This diversity is echoed in the broader postmodern understanding of subcultures, which emphasizes fluidity, lifestyle choices, and varying degrees of commitment (Frank, 2019; Kimberly & Hans, 2017).

In summary, swingers within the CNM framework are best defined as individuals or couples who, with mutual consent, engage in sexual activities with others, typically within social environments. This practice is distinct from other forms of CNM due to its focus on sexual rather than emotional or romantic connections with external partners.

### Methodological Challenges

Research on swingers has long faced challenges such as regional sampling biases and access issues (Jenks, 1998; Niekamp et al., 2013). Vaynman (2024) criticized the lack of comprehensive methods and theory, and Rubin et al. (2014) noted that techniques like snowball sampling are prone to bias. To address these issues, researchers have adopted more advanced methods, such as online surveys (Platteau et al., 2016) and data harvesting from a UK-based swinger website (Haywood, 2022). Data harvesting has the particular advantage that the result quality does not rely on response rates and that participation is not restricted to persons belonging to the same peer groups, ensuring more comprehensive and unbiased data collection. By using data harvesting, this study leverages these advantages.

As critically described by Frank (2019) in detail, previous research on swingers has sometimes encountered resistance within the academic community, with accusations of bias (Conley et al., 2018), and a tendency to focus on negative aspects such as STI risks (e.g., Dukers-Muijrers et al., 2010; Friedman et al., 2017; Niekamp et al., 2013), whereas in the long term, it was established that swingers did correspond to the normal clinic population in terms of STI prevalence (Dukers-Muijrers et al., 2017). Anderson (2010) saw the so-called monogamy script as widely viewed as authoritatively compelling in the research context. In this study, I maintain a strictly neutral position on swinging, focusing on statistical outcomes without excessive interpretation.

### Earlier Research Results on Size and Demographics of the Swinger Scene

As a consequence of the described methodological challenges, not much is yet known about the demographic composition of the group of individuals who, using one of the existing definitions, can be considered swingers.

Regarding social background, Jenks (1998) reported the assumption, which persisted until the 1990s, that the typical American swinger was a “white, middle to upper middle-class person in his or her late 30 s who is fairly conventional in all ways” (p. 510). Sheff and Hammers (2011) reached similar conclusions. O’Byrne and Watts (2011) found that swingers in their studies were in their 40 s and more likely to be educated and economically well off. Haywood (2022) calculated an average age of 43 for females and 45 for males who provided feedback on swinger events. Cerwenka et al. (2020) reported with regard to Germany that partner swapping and swinging are most prevalent among individuals aged 45–60, with 12% of men and 7% of women reporting current or past experiences with swinging, compared to just 3% for both sexes aged 18–30. These studies therefore suggest an ostensible profile of a typical swinger.

In contrast, research on polyamorous individuals has yielded different results. In particular, Moors et al. (2021), referring to the USA, found that previous engagement in polyamory was more likely among individuals with lower levels of education. Balzarini et al. (2019), diverging from Haupert et al. (2017), reported an association of polyamorous persons with lower income levels. This indicates that findings related to specific subgroups cannot be considered representative of the CNM population as a whole.

### Contextual Background: the German Swinger Scene

In this exploratory research, I aim to overcome some constraints in accessing swingers by focusing on the well-organized German swinger scene. In Germany, swinging is not handled as a clandestine affair. This contrasts sharply with societies where more rigid attitudes on sexuality prevail, notably the UK, as reported by Haywood (2022).

Swinging is legally permissible in Germany under general laws concerning consent and age. Swinger clubs, often licensed as restaurants, operate without facing allegations of immorality, as evidenced by several rulings from German courts (Bundesverwaltungsgericht, 2002, 2022; Rheinland-Pfalz, 2021). These decisions underscore the principle of individual sexual autonomy and lack of societal harm in such activities. Participation in swinging does not result in legal disadvantages, such as job loss due to extramarital relations (Mayer, 2013), nor does it lead to family law repercussions based on assumptions of an unwritten public order, as seen previously in Spain (Navarro, 2017). Swingers’ rights to privacy and personal freedom in their sexual lives are respected, aligning with broader societal and legal norms. The German media, also tabloids (Selig, 2023; Witte, 2023), tend to portray swinger clubs neutrally or positively, without invoking public outcry or sensationalism. This contributes to a normalized view of swinging activities within society. Swinging activities and clubs in Germany operate openly, without the need for secrecy. This is exemplified by the visibility of clubs such as “Steinenhaus” in Hattingen, which is even recognized in public transport networks by naming a bus stop after it (Verkehrsgesellschaft Ennepe-Ruhr mbH & Verkehrsverbund Rhein-Ruhr AöR, 2020). Joyclub, the major online platform for the swinger community, also reflects this openness.

Therefore, the German context offers an opportunity for transparent and open research into the swinger community, contrasting with the more clandestine approaches in other regions.

### Research Aims

By providing updated demographic data and insights into the German swinger scene, this research seeks to offer a more nuanced understanding of swingers, test existing stereotypes, and lay a foundation for future studies. In parallel to Niekamp et al. (2013), I focused on swinger website use as well as attendance at physical swinger events. The aim was to reassess assumptions about the typical age of swingers, gender composition, age differences in couples, and, based on regional indicators, the social positioning of the phenomenon in terms of income, occupational status, and migration background. I also examined whether swinging is primarily a practice of couples or includes significant numbers of solo participants, and whether participation by men and women occurs at similar levels, as observed in earlier studies of CNM (Rubin et al., 2014). These aims were addressed through two distinct but thematically connected studies.

The specific aim of Study 1 was to identify structural characteristics of the swinger scene in Germany based on verifiable indicators: age distributions, gender composition, couple and solo structures, and regional density patterns. These indicators were examined in relation to social variables such as income level, occupational sector, and migration background to assess the broader social positioning of the phenomenon. A follow-up data collection extended this analysis to subgroups defined by self-declared interest in BDSM, open relationships, and polyamory.

Study 2 examined publicly announced swinger events to capture how swinging manifests in physical space. It aimed to establish the age structure of admitted visitors, differences between solo and couple admissions, and the event types associated with different age and gender profiles. Additional attention was given to pricing structures, gender-based access rules, and their effects on participation. The spatial distribution and reach of events were analyzed using residence data of registered visitors. Long-distance attendance and its correlation with event pricing were examined to approximate the commitment and travel behavior associated with different event types.

## Study 1: Localized Swinger Profiles

### Method

Data from residents of selected counties of Germany who could be identified as swingers were harvested from the Joyclub (www.joyclub.de) online community, a platform dedicated to recreational intimacy, and were analyzed. The data retrieved, which consisted of age, gender, and the residence data at the county level, were anonymized from the onset. In the case of couples, both ages of the partners were collected and saved as one case.

#### Process of Data Selection from the Joyclub Website

The data selection process involved a series of structured steps, including the identification of the platform-based data source, the application of specific inclusion criteria, and the construction of the final data set.

##### Data Source: Joyclub Website

The Joyclub platform enjoys visibility, in part, due to mentions in the national media landscape (Ponath, 2022; Süddeutsche Zeitung, 2012). The veracity of the claim that the number of active member profiles amounts to upwards of five million remains unverified. According to traffic data from SimilarWeb (n.d.), which provides estimates based on a variety of data sources, Joyclub.de was ranked as the 66th most visited website in Germany in August 2024, placing it alongside major platforms such as Lidl.de (ranked 64th), Microsoft.com (ranked 65th), and Vodafone.de (ranked 68th). This high ranking underscores Joyclub’s prominence in Germany’s online landscape, indicating substantial user engagement and reinforcing the platform’s significance for research into online swinger communities. Since its establishment as a significant player in 2012, the website has fostered a distinctive business model primarily centered around user authenticity and quality of experience, abstaining from the utilization of paid advertising. This approach appears to have contributed to its robust financial health in 2022, marked by an appreciable increase in sales and a solid equity ratio (Ackermann & Noack, 2024).

Central to the business model is a rigorous mechanism aimed at mitigating the presence of fake profiles. Comprehensive verification processes, including video and on-site validations requiring official identification, are employed to uphold the integrity of user profiles (Joyclub, n.d.-a). The platform’s operational practices suggest a prioritization of authentic and quality user experiences, although this has not been formally evaluated in scientific studies.

German court rulings have clearly established that it is illegal for platform providers to employ fake profiles on dating platforms, with severe penalties for violations (Landgericht Berlin, 2022; Landgericht Flensburg, 2022). Attempts by platforms to justify such conduct through terms and conditions have been dismissed. Legal protections do not guarantee compliance, but the high likelihood of detecting violations, combined with the threat of penalties and reputational damage, enhances the credibility of Joyclub’s assertion that it does not utilize fake profiles. Additionally, platforms using fake profiles often follow a revenue model that charges per interaction (Verbraucherzentrale Bayern e.V., 2017), which is not the case with Joyclub. Details are provided in part B.1–2 of the Supplementary Material.

Joyclub membership at its foundational level is complimentary, but there exist options for upgrades. For instance, a premium membership entails entitlements such as a minimum 10% discount on entry fees for events facilitated by participating organizers once registered through Joyclub (Joyclub, n.d.-c). Joyclub profile configurations allow for pseudonymous nicknames, incorporation of images, disclosure of sexual inclinations, and textual inputs structured within the platform’s framework. By default, profile contents are accessible to all Joyclub registrants. A dedicated search feature facilitates member discovery based on variables such as gender, age, locale, and sexual predilections (Joyclub, n.d.-d).

##### Structural and Terminological Basis for Profile Classification

In compliance with ethical standards and to minimize bias, the terminology in this research reflects the demographic composition of the user base of Joyclub. An analysis of the Joyclub profiles relating to entries across Germany indicated a predominant orientation toward heterosexual and bisexual preferences, with 0.21% of the profiles showing identification with exclusive same-gender preferences. Details are provided in the data package for this study (Maor, 2025a, 2025b). Although Joyclub allows for registrations indicating “diverse” and multiple gender identities, a manual, hands-on review of search and event pages showed their minimal presence within the scope of this study.

Accordingly, the term couple is used to denote pairings of women and men, mirroring the predominant user demographic and accurately representing the heterosexual population of the study. Profiles on Joyclub not categorized as belonging to a couple are referred to as solo in this research. This designation highlights the type of profile rather than relational status, adhering to the privacy considerations integral to the research design.

##### Selection of Representative Counties

To limit the effort of data collection, not all suitable profile data from Germany were called up. Therefore, a representative selection of counties had been made. The selection also enabled structural alignment between the two studies, as the verification of Study 1 through independently gathered data in Study 2 required a consistent regional framework.

Germany is divided into states, intermediary regions, counties, and independent cities. Berlin and Hamburg, as city-states, operate as singular entities, without further subdivision. In line with the European Union’s Nomenclature of Territorial Units for Statistics (NUTS), counties and autonomous cities typically correspond to the NUTS-3 classification (European Commission: Eurostat, 2022). City-states function at the NUTS-2 level, bypassing the NUTS-3 designation (Statistisches Bundesamt, n.d.).

For simplicity, the term counties will refer to all administrative regions included in this study, encompassing counties, independent cities, and the city-states of Berlin and Hamburg.

For the selection of counties that are representative for Germany as a whole, principal component analysis was conducted on official data from German counties to identify 24 representative counties that reflect the country’s demographic and structural characteristics.

To identify a representative sample of German counties, a principal component analysis (PCA) was conducted using SPSS 29.0.1.1. Input variables included structural data from all 400 German counties, comprising average disposable income, population density, total population, and age distribution across seven age strata (from 30 to 75 years).

The suitability of the data for factor analysis was confirmed prior to performing PCA. The extraction was conducted using the correlation matrix. Components with eigenvalues exceeding 1.0 were retained (Kaiser criterion) and no rotation was applied, as the primary goal was dimensional reduction for sampling purposes rather than interpretation of underlying factors.

The PCA yielded three principal components with eigenvalues greater than 1, collectively explaining 80.57% of the total variance (Component 1: 47.51%, Component 2: 21.60%, Component 3: 11.46%). The first component strongly correlated with population density (0.800) and younger age groups (30–34 years: 0.913; 35–39 years: 0.805) positively, while negatively correlating with older age groups (55–59 years: − 0.877; 60–64 years: − 0.907; 65–75 years: − 0.823). The second component showed strong associations with average income (0.767) and middle-aged groups (45–49 years: 0.884; 50–54 years: 0.705). The third component exhibited strong correlation with the 40–44 age group (0.716) (see Table S1 in the Supplementary Material).

Based on these PCA results, 24 counties were selected that represented diverse combinations of factor scores across all three components. Counties near the median values of the component scores (e.g., Ilm-Kreis, Frankfurt (Oder), Düren, Traunstein) were included to ensure balanced representation across the sociodemographic spectrum. The selection deliberately also included economic outliers: the wealthiest (Starnberg and Hochtaunuskreis) and the poorest counties in Germany (Gelsenkirchen and Duisburg), measured by average available income. For East Germany, Potsdam-Mittelmark was included as an economic outlier (highest income in East Germany). Leipzig and Halle were selected as population density outliers in East Germany. This selection strategy allowed for examining whether extreme sociodemographic characteristics correlated with swinger demographics.

##### Assessment of the Demographic Data of the Representative Counties

At a 95% confidence level, this selection represents a cross-section of the national demographic and occupational landscape across the examined variables, namely average disposable income, population density, total population, and age distribution across seven age strata, as well as workforce participation in the service sector and in occupations in sectors related to mathematics, information technology, natural sciences, and technology, as defined in German statistical classifications (MINT), and divorce rates in 2021, normalized per 100,000 residents. For population density, I used a population-weighted national average as a benchmark rather than the simple mean of county-level densities, which would overrepresent sparsely populated regions, and not reflect the average environment in terms of population density which the inhabitants were living in. The 24 counties identified in this manner are referred to as representative counties.

Spearman bivariate correlation analysis, accompanied by bootstrap calculations, was applied to the sociodemographic data of the representative counties, excluding age-related variables, and including data on the quota of inhabitants having an immigration background, to identify correlations between the variables of these data. The assumption of normality was not met; Spearman’s rho correlation analysis is a nonparametric test, which is robust against nonnormal distributions.

##### Selection Criteria with Respect to Profiles

The search function on the Joyclub website displays results with profile nicknames, ages (or, in the case of couples, both ages), and the county of residence (Joyclub, n.d.-d). For mixed-gender couples, the woman’s age appears first. Each search result links to the full profile page.

The search function includes filters that allow the display of profiles matching specific parameters. For this study, I identified swingers based on users’ self-identification as swingers in their profiles. I applied filters to include only profiles that were verified by Joyclub through their verification routines. Additionally, I used only profiles with a profile picture, as one of my inclusion criteria. In the following discussions, individuals with profiles meeting these criteria (verified profile, bearing a profile picture, and self-identification as swingers) will be referred to as identified swingers.

##### Collection of Swinger Data

Using these filters, I searched for profiles within a radius that extended beyond the maximum distances possible within each county. Searches were conducted separately for men, women, and couples across the 24 representative counties. In the case of Leipzig, both the city and the surrounding county were treated as a single unit, as Joyclub displays both under “Leipzig” in the search results. The county data for Leipzig were adjusted accordingly.

Only the results pages were accessed, and not the profile pages themselves. This was sufficient to meet the research aims, because the results page output can be filtered to show only men, women, or couple profiles, and show the relevant data, namely the age and the county of residence.

In this way, I harvested the search results as raw data. From these, I extracted the age or ages and ascertained that the place of residence of each profile entry referred to the target county. Ultimately, I inserted these extracted data, pertaining to the age, the residence county, and the gender information, into the corpus. In case of couple profiles, I retained the link between the man and the woman in the respective couples. I performed these steps described using Python scripts, which are contained in the data package for this study (Maor, 2025a, 2025b). After random testing for accuracy of the results provided by the scripts, I deleted the raw data.

In addition, I conducted the same search and harvesting with regard to the four cities in Germany with more than one million inhabitants, referred to as big cities: Berlin, Munich, Hamburg, and Cologne.

##### Assessment of Duplicate Profiles

Because the extrapolation of the overall number of identified swingers and the interpretation of demographic distributions rely on profile-based data, it was necessary to assess whether—and to what extent—profile duplication could affect the results. I therefore estimated the prevalence and structural impact of duplicate profiles across the data set.

Two duplication scenarios must be distinguished. The first involves a person maintaining a solo profile and also partaking in a couple profile, which is allowed under Joyclub’s rules (solo-couple duplication). The second pertains to profiles of the same type—two solo profiles of the same gender or two couple profiles—managed by the same individual or couple (same-type duplication).

Theoretical frameworks of online identity management (Bullingham & Vasconcelos, 2013), contextual identity representation (Hogan, 2010), and research on identity construction in anonymous online environments (Zhao et al., 2008) suggest that solo-couple duplicates should not be interpreted as deceptive. Rather, such profiles reflect different social contexts in which swingers operate, allowing them to present themselves both independently and as part of a couple, depending on the situation. The same rationale applies to cases in which a person maintains a solo profile and, for example, a second profile as an artist—something commonly done by authors or photographers.

To accurately estimate the prevalence of duplicate profiles and to understand their impact on the findings, I developed two Python scripts to calculate both solo-couple duplication and same-type duplication rates. These scripts compared real data from the representative county set (proven to be representative for Germany) against randomly generated duplication patterns using Monte Carlo simulations. The simulation parameters were informed by data independently collected in Study 2. For solo-couple duplications, county-specific age distribution data from official sources (see Part H of the Supplementary Material) further refined the estimates.

The scripts computed the divergence between the real data set and the synthetic duplication scenarios, accounting for variables such as age, gender, and location. A third Python script applied a bootstrap procedure with 10,000 iterations to assess the robustness of the solo-couple duplication estimates. For same-type duplications, the bootstrap was integrated into the respective script. All scripts, along with the detailed methodology, are included in the data package accompanying this study (Maor, 2025a), and a complete explanation is provided in Sections B.6 and B.7 of the Supplementary Material.

#### Data Analysis

The following analyses were based on the previously described data corpus, which combined profile-level information on identified swingers with structural county-level indicators. The swinger data set contained age-related information for solo and couple profiles, enabling the calculation of gender-specific median ages and density measures. The county-level structural data included all variables used for county selection and representativity assessment—namely, average disposable income, population density, total population, age distribution across seven strata, divorce rate, and workforce participation in both the service sector and in MINT-related occupations. In addition, the quota of residents with a migration background was included for use in correlation analyses.

The analyses described below were carried out to characterize the swinger population, to assess structural correlations with demographic patterns, and to explore potential clustering of counties based on their socioeconomic environments. Each step is described in detail in the subsections that follow.

##### Descriptive Data Analysis

From the collected data, I calculated the median ages of men, women, solo men, solo women, and the median age difference in couples, together with the standard deviations.

In a further step, I calculated a variable that will be referred to as swinger density, representing the number of identified swingers normalized against 100,000 inhabitants of the county. In the same way, with the aim of detecting possible gender differences, I calculated density rates for women, men, solo women, and solo men.

##### Correlations Assessment

The correlations between the aggregated age data of identified swingers, the age difference in identified swinger couples, the swinger density rates, and the county structural data were examined. A bivariate Spearman’s rho correlation analysis, accompanied by 1000 bootstrap calculations, was used as the data did not meet the assumption of normality and this nonparametric test is robust against nonnormal distributions. Statistical power was calculated post hoc using the software G*POWER, Release 3.1.9.7 (Faul et al., 2009), and in all cases reached the typical threshold of 80%.

##### Cluster Analysis

To enable a more nuanced interpretation of sociodemographic influences on swinger profiles, I conducted a cluster analysis. This approach was chosen for two main reasons. First, analyzing correlations between swinger characteristics and each sociodemographic variable individually would not adequately capture how these factors interact within integrated structural contexts. Second, the presence of multicollinearity among structural variables—identified through the correlation analysis of county-level data—made it difficult to interpret their independent effects in isolation.

To therefore allow better interpretation when analyzing data at the county level, I used IBM SPSS software in version 29.0.1.1 to identify four clusters from the 24 representative counties based on the structural county data, excluding those related to age. These clusters will be referred to as sociodemographic clusters. Using the corpus, in which I had amended the structural data of the county of residence to each case, the clustering was normalized against the numbers of swinger cases, thus reflecting the environments in which the identified swingers live. Therefore, the cluster membership of the county of residence can be considered a variable broadly reflecting the personal living environment of the persons studied. Details of this clustering process are provided in a file within the data package for this study (Maor, 2025a). Then, the mean and median swinger data for each of the clusters were calculated.

This calculation was replicated for the data pertaining to each of the big cities (Berlin, Cologne, Hamburg, and Munich). The objective was to uncover any potential variations attributable to different urban contexts.

##### Analysis of Subgroups

In August 2024, I collected data on identified swingers who declared BDSM as an interest or who indicated open relationship or polyamory as their relationship status. I used the corresponding filter criteria and otherwise followed the same methodology as described above.

##### Extrapolation of Swinger Prevalence to a Germany-Wide Total

The findings on solo-couple duplication and the representativeness of Study 1 data enable an approximation of the total number of persons in Germany meeting the identified swinger criteria. This figure has also been estimated.

### Results

A total of 9325 women and 13,648 men were recorded as identified swingers, of which 6,077 each were in couple profiles. This means that the ages and places of residence of 3248 solo women and of 7571 solo men were included in the data pertaining to the representative counties and the big cities. From the 24 representative counties, data of 3135 identified swinger women, with 917 solo women, and of 5256 identified swinger men, with 3038 solo men, were harvested. Unless otherwise specified, the following data will be based on the data collected from the representative counties.

#### Age of Identified Swingers in the Sample

**Fig. 1:**
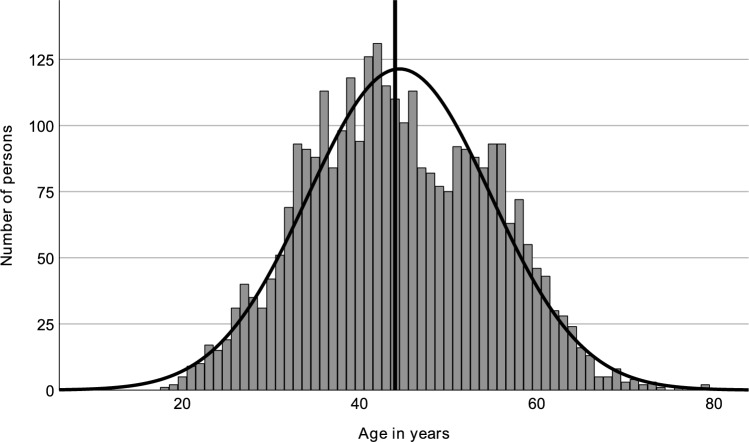
Age distribution of women with verified Joyclub profiles (including a picture) who self-declare as swingers, from 24 German counties selected for their sociodemographic representativeness. The vertical line represents the median age (44.0 years)

**Fig. 2:**
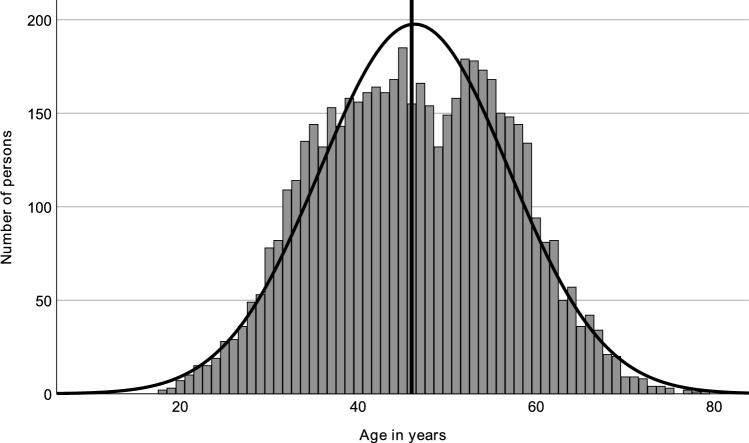
Age distribution of men with verified Joyclub profiles (including a picture) who self-declare as swingers, from 24 German counties selected for their sociodemographic representativeness. The vertical line represents the median age (46.0 years)

For identified swinger women, the median age was found to be 44 years (95% CI [43, 44]), and for men, the median was 46 years (CI [46, 47]), as visualized in the accompanying histograms, which illustrate the age distribution of identified swinger women and men, respectively; see Fig. 1 regarding women and Fig. 2 regarding men.

Kurtosis for the age of women was − 0.518, and skewness was 0.98. For men, these values were − 0.599 and 0.002, respectively. They suggest a relatively symmetrical distribution around the mean, as shown by the bell-shaped curves overlaid on the histograms. The kurtosis for the age difference within couples was 2.182, paired with a skewness of 0.493, pointing to a heavier tail distribution with a slight tendency toward larger age differences where the man is older.

**Fig. 3:**
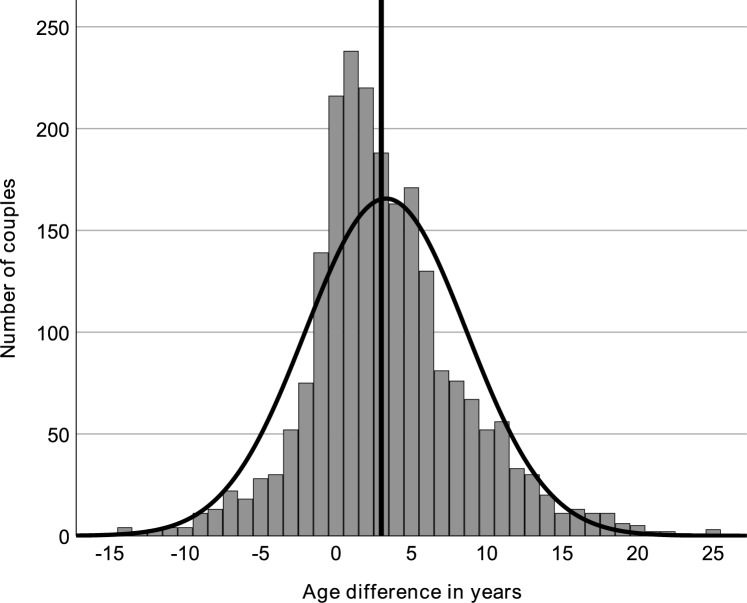
Age difference in couples with verified Joyclub profiles (including a picture) who self-declaring as swingers, from 24 representative German counties selected for their sociodemographic representativeness. The vertical line represents the median age difference (3.0 years)

Focusing on the age difference across couples, see Fig. 3, the 5th percentile indicated an age difference of -5 years (95% CI [− 5, − 4]), where negative values signify that the woman in the couple is older. Conversely, at the 95th percentile, the age difference was + 13 years (CI [+ 12, + 13]), where positive values indicate that the man in the couple is older. These percentile values show the range of age differences observed and variability in the age differences among couples within the study sample.

The median estimates from the distribution show moderate precision, with standard errors ranging from 0.01 to 0.50 across age percentiles, derived from 10,000 bootstrap samples. This level of precision affirms the reliability of the age characteristics in the sampled population. In the bootstrap exercise, weighted averages were calculated by normalizing weights to preserve the actual sample size, aligning the sum of weights with the number of observations. This method upholds the sample’s representativeness.

#### County-Related Assessment of Ages

For women, the range of median ages in the individual counties included in the sample was from 41.0 years (*n* = 812, *SD* = 9.86; Leipzig) to 51.0 years (*n* = 24, *SD* = 12.38; Lüchow-Dannenberg). For men, the values ranged from 43.0 years (*n* = 233, *SD* = 9.24; Halle [Saale]) to 53.0 years (*n* = 31, *SD* = 11.13; Lüchow-Dannenberg).

The median age difference among couples was two years in 9 counties and three years in 12 counties. In one county, an age difference of one year was found (*n* = 24, *SD* = 7.42; Sömmerda); in another county, the median age difference was four years (*n* = 87, *SD* = 5.54; Hochtaunuskreis). The highest median age difference found in any county was five years (*n* = 28, *SD* = 3.56; Starnberg). The detailed aggregated county-related age data are contained in Table S2 in the Supplementary Material.

#### Correlations of Swinger Density in Clusters

The sociodemographic clusters, identified through the analysis described in the Methods section, are presented in Table S3, with detailed features shown in Table S4, both in the Supplementary Material. Clusters 1 and 2 contain rural settings and mid-sized cities, with cluster 1 showing a higher average disposable income and a higher proportion of residents with a migration background than cluster 2. Cluster 3 comprises cities with comparatively low wealth, and the two counties in cluster 4 are semiurban and show a high average income.

**Fig. 4:**
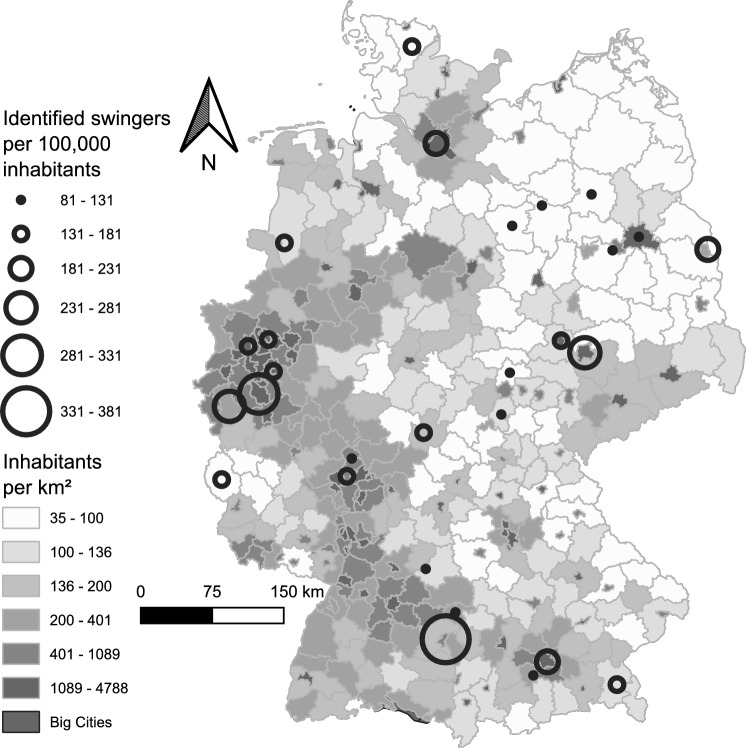
Number of identified swingers (verified Joyclub profiles with a picture, self-declaring as swingers) per 100,000 inhabitants across German counties. Circle sizes indicate the ratio of identified swingers; shades of gray represent county population density. County boundary data © GeoBasis-DE/BKG (2023) (data modified). Population density source: Statistisches Bundesamt (2022). Licenses for both datasets: dl-de/by-2-0 (FITKO, n.d.). A location key is provided in Supplementary Material Figure S3

**Fig. 5:**
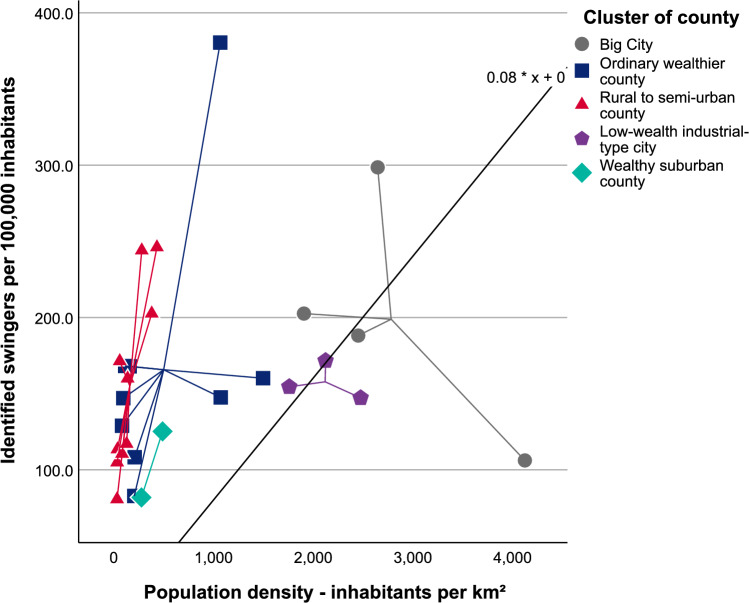
Number of persons maintaining a verified Joyclub profile with a picture, self-declaring as swingers (identified swingers), per 100,000 inhabitants in relation to the population density (inhabitants per km^2^) in 24 representative counties selected for their sociodemographic representativeness of Germany, and the cities of Berlin, Cologne, Hamburg, and Munich

The clusters distinctly represented the typologies of various counties in Germany, but the numbers of identified swingers within these clusters, normalized against 100,000 inhabitants, showed variation. No clear pattern or regularity emerged in this context. The numbers fluctuated within the clusters and did not follow a geographical alignment, as also clearly visible in Figs. 4 and 5.

In the ordinary wealthier counties, the swinger density ranged from 82.9 in Ostalbkreis to 380.5 in Ulm. In the rural to semiurban areas, the lowest density was 80.7 in Prignitz, with the highest density in Leipzig (city and county) at 246.2, closely followed by Düren at 244.1. Among the low-wealth cities, Gelsenkirchen, the German county with the lowest available per capita income, registered the lowest density at 147.2, whereas Duisburg, with the second lowest per capita income in the country, recorded a density of 171.6. In the wealthy county subset, Starnberg had a density of 81.9 and Hochtaunuskreis identified 125.3 swingers per 100,000 inhabitants. The figures for major cities also varied significantly, ranging from 106.2 swingers per 100,000 inhabitants in Berlin to 298.6 in Cologne, the latter being the second highest value observed. The number of identified solo women swingers per 100,000 inhabitants also varied and ranged from 2.3 in Altmarkkreis Salzwedel to 36.7 in Cologne. The density of solo men varied wider and ranged from 24.8 in Lüchow-Dannenberg County to 151.2 in Ulm. In Ostalbkreis County, 58.4% of all identified swinger women were solo women, whereas in Altmarkkreis Salzwedel, the corresponding share amounted to only 7.0%, with the lowest value detected.

Detailed information, including gender-specific data and distinctions between all swingers and solos, is available in Table S5 in the Supplementary Material.

#### Correlations Within Swinger and Sociodemographic Data

As detailed in Table S6 in the Supplementary Material, significant correlations were found within the sociodemographic county data. These include relationships between population density and migration background rate, between tertiary and MINT employment ratios, and between migration background rate and average income. Knowing these correlations is essential for interpreting correlations between swinger data these sociodemographic variables. No correlation was found with the number of divorces per 100,000 inhabitants.

The analysis of correlations between swinger densities, age data, and sociodemographic data yielded significant correlations, as shown in Table 1. Again, no significant correlations were found with the divorce rate. The complete correlation matrix is contained in Table S7 in the Supplementary Material.

**Table 1:** Correlations of swinger-related data and sociodemographic county data in the representative set

Value	Average available income in the county in Euro		Quota of persons employed in the tertiary sector^a^		Quota of persons employed in the MINT^b^ sector^a^		Population density^c^		Proportion of inhabitants with a migration background in %	
	*r*_s_(23)	*p*	*r*_s_(23)	*p*	*r*_s_(23)	*p*	*r*_s_(23)	*p*	*r*_s_(23)	*p*
Median age of men^d^	.46 [.04, .78]	.025	.11 [− .34, .54]	.597	.05 [− .38, .45]	.833	− .20 [− .61, .23]	.354	.13 [− .30, .58]	.556
Median age difference in couples^d, e^	.35 [− .07, .69]	.093	.41^ g^ [− .13, .74]	.045	.41^ g^ [− .03, .72]	.045	.35 [− .07, .69]	.093	.39 [− .06, .69]	.057
Swinger density^d, f^	− .14 [− .54, .32]	.511	.17 [− .26, .57]	.416	.16 [− .30, .57]	.445	.46 [.09, .72]	.023	.33 [− .10, .70]	.113
Density of solo swinger women^d, f^	− .17 [− .56, .33]	.426	.33 [− .12, .71]	.116	.27 [− .17, .66]	.200	.65 [.33, .80]	< .001	.39 [− .07, .73]	.063
Density of solo swinger men^d, f^	− .18 [− .57, .30]	.391	.02 [− .36, .43]	.920	.29 [− .19, .69]	.169	.60 [.24, .82]	.002	.44 [.04, .75]	.029
Density of swinger men^d, f^	− .16 [− .55, .31]	.463	.14 [− .29, .53]	.517	.15 [− .32, .60]	.485	.46 [.10, .72]	.025	.31 [− .10, .66]	.144

*r*_s_ values represent Spearman’s rho correlations. Only correlations between age and density variables with sociodemographic variables are shown. Values in square brackets indicate the 95% confidence intervals for each correlation. Bias-corrected and accelerated confidence intervals were calculated using bootstrapping with 1000 iterations

^a^In relation to all employees, in percent

^b^The acronym “MINT” relates to sectors related to mathematics, information technology, natural sciences, and technology (as defined in German statistical classifications)

^c^Inhabitants per km^2^

^d^The figure relates to swingers identified by a verified Joyclub profile with a picture, self-declaring as swingers

^e^Positive values indicate that the man is older

^f^Rates are normalized by 100,000 inhabitants in the county of residence

^g^Although the correlation is statistically significant at *p* < .05, the bootstrapped 95% confidence interval includes zero, indicating that the correlation may not be robust. This suggests that any observed correlation should be interpreted with caution

The findings indicate interdependencies among swinger demographics and certain sociodemographic factors. Generally, population density correlate with swinger density rates, and age data correlate with income figures and the factors correlated to it, involving the migration quota and, with the caveat that bootstrapping results require careful interpretation, the occupation structure. The moderate positive correlation between swinger men density and the proportion of inhabitants with a migration quota forms an exception in this regard.

#### Subgroup Analysis

In the counties of the representative dataset, the *density*—defined as the number of swingers per 100,000 inhabitants—of individuals expressing interest in BDSM reached 37.4. The same dataset revealed a density of 22.1 for swingers who declared an open relationship as their relationship status and 4.5 for those identifying with polyamory. These subgroups exhibited a higher prevalence in major cities compared to other counties. For instance, Berlin recorded a density of 52.5 for BDSM swingers, 34.2 for open relationship swingers, and 8.6 for polyamorous swingers. Cologne displayed the highest density, with 78.1 BDSM swingers. Additional details can be found in Table S13 in the Supplementary Material.

Significant correlations of the subgroup data, collected in August 2024, with the main dataset from 2023 were found regarding median ages, identified persons normalized against the respective county population, and variations of such densities among counties and between subgroups. The number of BDSM swingers per 100,000 inhabitants and the quota of persons employed in the MINT sector in percent correlate with *r*_s_(23) = 0.59, *p* = 0.003, 95%CI[0.28, 0.81]. In addition, some correlations found within the structural data from the main dataset were also identified in the subgroup sets. More detailed results are reported in Tables S7 to S12 in the Supplementary Material.

#### Use of Duplicate Profiles

The analysis revealed an estimated number of 1,734 solo-couple duplications (95% CI[1561, 1908]) in counties including big cities, potentially affecting 610 solo women (CI[526, 700)] and 1124 solo men profiles (CI[994, 1261]). This represents 16.0% (CI[14.4, 17.6]) of the total 10,819 solo profiles in the dataset including the big cities. When excluding big cities, the estimated solo-couple duplications drop to 748 individuals (CI[673, 820]), affecting 248 solo women (CI[215, 283]) and 501 solo men profiles (CI[435, 571]), representing 18.9% (CI[17.0, 20.7]) of the 3,955 solo profiles in the dataset without big cities.

Moderate to strong positive Spearman correlations were observed between these duplications and the population size (including big cities: *r*_s_(27) = 0.92, *p* < 0.001, 95% CI[0.83, 0.97]; excluding big cities: *r*_s_(23) = 0.43, *p* = 0.023, CI[0.04, 0.71]), the population density (including big cities: *r*_s_(27) = 0.50, *p* = 0.007, CI[0.12, 0.77]; excluding big cities: *r*_s_(23) = 0.62, *p* < 0.001, CI[0.28, 0.83]), and the swinger density values (including big cities: *r*_s_(27) = 0.51, *p* = 0.006, CI[0.15, 0.77]; excluding big cities: *r*_s_(23) = 0.59, *p* = 0.001, CI[0.25, 0.81]), with all 95% CI values being based on 1,000 bootstrap samples.

Spearman analyses of the data adjusted by the duplications did not reveal other correlations than the non-adjusted data. The correlation of non-adjusted data with population density remained consistent after adjustment, with the result for the adjusted data being situated within the confidence interval of that based on unadjusted data (see part B.7 of the Supplementary Material for details).

In the representative dataset, the likelihood of same-type duplicate profiles across all categories is minimal, with estimated duplications affecting between 0.9% and 1.1% of profiles. These low percentages suggest that the prevalence of same-type duplicate profiles is not significant enough to introduce bias or inaccuracies in the analysis. The reliability of the predictive model was further validated by including the larger cities, where the model predicted overlaps with near-perfect accuracy. Therefore, the data can be considered highly reliable in this respect, and the observed differences between expected and actual duplications are well within the acceptable range.

#### Germany-Wide Total Prevalence of Identified Swingers

Extrapolating values from the representative dataset and factoring in estimated solo-couple duplications, 128,557 individuals (95% CI[127,361, 129,836)] with 48,567 women (CI[47,960, 49,105]) and 79,990 men (CI[78,811, 81,101]), all aged between 18–74, are assumed to meet the identified swinger criteria in Germany (self-declared swingers using verified Joyclub profiles with a profile picture). This corresponds to 0.21% of the German population aged 18–74. For women, the value amounts to 0.16%, and for men to 0.25%. Details of this calculation are reported in part C of the Supplementary Material.

### Discussion

The median ages of identified swinger men (46 years) and women (44 years) in Germany slightly exceed those reported in the UK by Haywood (2022), in Dutch STI clinics by Dukers-Muijrers et al. (2010), and those found by O’Byrne and Watts (2011) for visitors of a Canadian swinger club. A detailed comparative analysis of swinger demographics across these countries is outside the scope of this study.

The higher median age of men almost reflects the median age difference in couples, which is three years. This finding contradicts a possible stereotype of midlife men predominantly seeking much younger partners in swinging contexts. The correlation of men’s median age and the median age difference in couples with the wealth of their residential counties, whereas such correlation could not be found for women, might suggest that women participate in swinging, and are accompanied by their typically older partners.

In contrast to earlier suggestions to concentrate on couples in swinger research (Jenks, 1998; Vaillancourt, 2006), a considerable number of solo profiles were observed among identified swingers. This prevalence of solo profiles, particularly among men, indicates that participation in swinging activities extends beyond partnered contexts. In Germany, the prevalence of solo women profiles does not justify the label “unicorns” for unattached swinger women, a term Haywood (2022) used for the UK and Bergstrand and Sinski (2010) applied to the USA context. Nevertheless, the relatively lower representation of women in the dataset remains unexplained by the current data. In particular, the observation that the number of solo swinger women strongly correlated with population density, whereas the corresponding value for solo men was weaker, warrants further investigation to understand the motivations underlying these dynamics.

The findings contradict the portrayal by Friedman et al. (2017) of swinging as an activity predominantly associated with individuals in less affluent circumstances. The presence of swingers in wealthier counties challenges the notion that swingers are exclusively from impoverished or marginalized backgrounds. Furthermore, the lack of significant correlations between swinger density and factors such as divorce rates, employment in MINT occupations, or the proportion of residents with a migration background—except for a correlation with the latter in the case of solo men—further questions assertions by Jenks (1998). Contrary to Jenks’s view that swingers are typically not members of minority groups and generally come from wealthier or more educated backgrounds, this study’s findings suggest a more complex and diverse demographic. These results align with the earlier research by Rubin et al. (2014) and Levine et al. (2018), who defied assumptions about correlations between CNM prevalence and ethnicity, education, or income, while Balzarini et al. (2019) found that the latter two factors were relevant with regard to polyamorists.

Rates of tertiary sector and MINT employment correlate with larger age differences in couples, but they do not impact swinger density. This macro-level observation does not reflect the micro-level based findings of Balzarini et al. (2019) for polyamorists in the USA, who were reported to work significantly more often in information technology professions.

On a macroscopic level, some regional similarities in swinger density suggest the influence of very local cultural factors. For example, the similar swinger densities observed in Cologne and adjacent Düren County, and the relative homogeneity of age and swinger density data in Leipzig and neighboring Halle (Saale), support this idea of regional influence. Any precise prediction of swinger density based on sociodemographic factors, if at all possible, would thus prove to be challenging.

In essence, the results do not confirm that the relationship between socioeconomic factors and swinger demographics aligns with the traditional parameters used in earlier studies. Cross-cultural comparisons or those between different types of CNM practices might be challenging without a viable theoretical foundation, which yet has to be developed, and all comparisons require careful consideration of the level of analysis on which the results were generated.

Some correlations found in the BDSM and open relationship swinger subgroups, particularly regarding the county income, suggest that swinger demographics are influenced by latent factors that could not be covered in this study due to its design-related limitations. Future research should explore individual motivations and the cultural influences across regions to better understand the interaction between related factors.

The intersections among swingers and BDSM, open relationship, and polyamory subgroups in Germany require careful interpretation. Providing related data is optional for profile users. Therefore, strategic choices in identity presentation rather than comprehensive descriptions of individuals’ sexual practices are likely represented. The seemingly lower prevalence of open relationship and polyamory identifiers among swingers requires particularly careful interpretation. As noted by Haupert et al. (2017) and Cardoso et al. (2021), terms like open relationship and polyamory carry different connotations across cultural contexts. Schmidt (2023) identified a narrower definition of the German term Polyamorie compared to the English term polyamory, which may influence how individuals categorize their relationship. Rubel and Burleigh (2020) highlight the varied understandings of polyamory in the USA, with only 38.6% of participants at all showing some basic understanding of the term. Therefore, different interpretations may complicate demographic analysis based on self-identification, in particular across different languages.

Specifically, the particularly low prevalence of polyamory among swingers may simply reflect differences in terminology. Venn’s (2015, pp. 266–267) concept of “Cliquen-Swinging” refers to a pattern where swingers limit their sexual activities to a close-knit group or “clique” of trusted partners. These cliques often involve stable and repeated interactions, which may resemble “polyamory” according to some definitions and from an outside perspective, but participants may not identify with this term.

A theoretical limitation of this Study 1 concerns the prevalence of potential duplicate profiles. The possibility of same-type duplications cannot be dismissed, but the estimates show low values. Given the relatively narrow age distribution of swingers, the alignment between predicted and actual overlaps supports the robustness of the prediction model and the quality of the dataset. In contrast, the calculated frequency of solo-couple duplications is considerable. Population size and density appear to drive the corresponding user behavior. Nevertheless, after adjustment by the respective duplication estimates, the resulting correlations to demographic data remain substantially the same. This means that the profile duplications do not introduce meaningful bias into the study’s primary finding, which confirms the robustness of its results also in this regard. The results reported here therefore remain unaffected by both types of duplications.

## Study 2: Guests at Swinger Events

Study 2, in contrast to Study 1, focuses on observable behavior in the context of physical encounters by analyzing admissions to swinger events advertised on Joyclub. Swinger events represent a central format through which participants initiate contact and engage in shared experiences. The resulting data offer an independent empirical basis to assess the plausibility of patterns identified in Study 1 and to gain additional insights into preferences, travel behavior, and financial commitment as reflected in entrance fees and pricing structures.

### Method

In the second study, I therefore examined 82 swinger events that occurred on three Saturdays (September 16, September 23, and October 7, 2023) in Germany. The data harvesting techniques employed within the Joyclub portal (www.joyclub.de) to investigate these events were identical to those used for Study 1, unless stated otherwise.

The Joyclub platform offered event organizers the opportunity to advertise events for free and access to a complimentary guest management system with additional features. Joyclub profile holders were only included on the guest list upon activation by the event organizer. Event organizers could also integrate a registration option on their own platforms via a white-label solution, linking it to the Joyclub guest management system without guests noticing the connection to Joyclub. Such external registrations, and registrations manually added by organizers to the Joyclub guest management system, were displayed on the event listing page as “External” and specified by gender (men, women, couples), but not age information. Organizers had incentives to include external registrations, such as increased event visibility on the Joyclub platform and the ability to use statistics functions for participant management (Joyclub, n.d.-b). Events were listed more prominently when discounts to Joyclub Premium members were granted (Joyclub, n.d.-c). By designing the platform to be a central hub for event registration and management, Joyclub thus minimized the likelihood of admissions occurring outside its purview. Some organizers mandated Joyclub registration to assess potential attendees based on their profiles.

Criteria for the inclusion of events in this study were stringent: events had to permit sexual activity, have a minimum of 95 registered guests, and be recommended or quality-checked by Joyclub. To ensure a 95% confidence interval, additional filtering was applied: for broader analysis considering all events with available age data, events where external guest registrations comprised 20% or more (*n* = 8) were excluded. This filtration process ensured the desired confidence level, because the availability of age data for over 95% of the admitted guests, excluding companions of accompanied solos, was achieved. In total, 76 events were therefore included in the subsequent analysis, termed *adjusted total selection*. To ensure a 95% confidence interval for analyses where ages of persons admitted to single events were relevant, a smaller set of 53 events was defined, termed *strict selection*. Here, events were omitted where more than 5% of the total number of attendees were registered externally.

Leaning on the typology of Venn (2015), I categorized events based on the organizers’ descriptions in Joyclub. Category 0 represented standard swinger events, and Category 1 (“Food/dance-focused events”) encompassed events emphasizing dance facilities, upscale ambiance, dress codes, elaborate food, and expensive drinks. Mere customary dress codes or seasonal themes such as "Oktoberfest" alone did not warrant inclusion in Category 1, because dancing, food buffets, and various beverages were typical features of swinger events, usually included in admission fees, and therefore inadequate for category differentiation. Corresponding event advertising, such as DJ names or food references, was similarly insufficient. Category 2 (“Sex-focused events”) included events with specially organized and moderated sex games. Examples included simulated red-light district events where guests earned play money through various activities, as well as mock auctions of blindfolded men or women under partner supervision. Some events catered to specific fetishes, such as skin color, curvy bodies, or BDSM. Category 3 combined elements from the first two special categories. A detailed description of the events, as they were presented on Joyclub, and the categorizations are contained in Table S15 in the Supplementary Material.

After each event’s conclusion, I collected anonymous profile data, namely the ages and counties of residence, of individuals identifiable on Joyclub as admitted to attendance. Sources were the event pages listed in the data package for this study (Maor, 2025b) and in Table S15 in the Supplementary Material. The profiles were harvested separately according to the categories reflected on the event listing page, which were registered couples, individual men, and individual women.

In addition, I established the category of accompanied solos. Accompanied solos are individuals who, when registering for an event on Joyclub, indicate their intention to attend as a couple, despite using a solo profile for registration. They are categorized as couples on the event listing page, and the only discernible difference to couples registering using a couple profile is that only one age is provided, being the age of the solo profile owner. The gender of the solo profile owner could not be determined without accessing the respective personal profile. Due to privacy considerations and the commitment to data protection, this was not done. Instead, I introduced the additional gender category, accompanied solo, to encompass this group of visitors.

The data analysis included descriptive statistics on the age data of admitted visitors, information about the entrance price paid, and the distance between the displayed place of residence and the event venue, all in general and related to different event types. In addition, these data were separately assessed with respect to events classed as BDSM events on the basis of the event description.

### Results

**Fig. 6:**
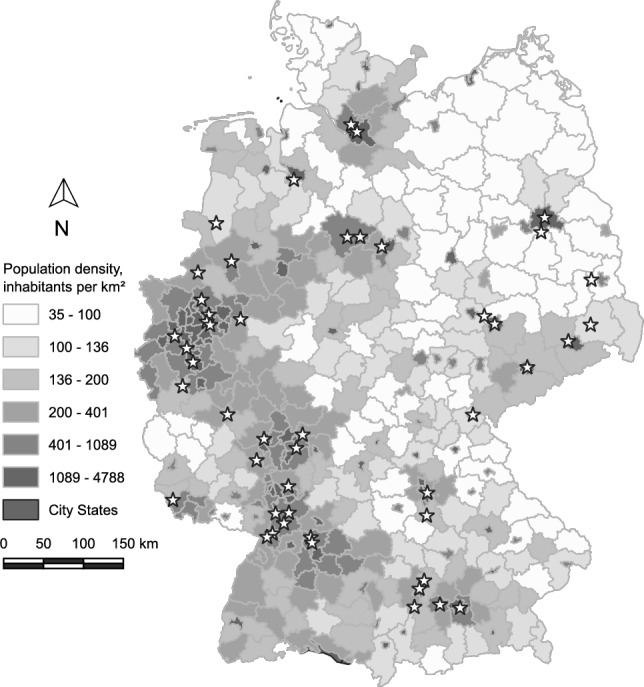
Locations of event venues in Germany referenced in the study. Stars indicate event venues; shades of gray represent county population density. State and county boundary data © GeoBasis-DE / BKG (2023) (data modified). Population density source: Statistisches Bundesamt (2022). Licenses for both datasets: dl-de/by-2-0 (FITKO, n.d.). A location key is provided in Supplementary Material Figure S4

The adjusted total selection sample included 16,159 admitted visitors, including 725 external registrants and 1426 solo attendees’ companions, with known age data for 14,008 individuals. Of the 76 swinger events, 52 were in general category 0 (“Ordinary sex event”), two were in category 1 (“Food/dance-focused event”), 21 were in category 2 (“Sex-focused events”), and one was in category 3. Admissions to the recorded events ranged from 95 to 522 (*Mdn* = 180.0, *M* = 213.6, *SEM* = 11.9). As illustrated in Fig. 6, the distribution of venues spanned across German territory, predominantly clustering in regions of higher population density, with a notable absence of events in the sparsely populated northeastern regions. The strict selection encompassed 10,784 visitors, including external registrants, and 10,089 visitors excluding accompanied solos’ companions. Age data were available for 9584 visitors in this set.

#### Age of Guests Admitted to Events

The median age of guests in the adjusted total selection was 42.5 years for women (*n* = 5356, *SD* = 9.7), 44.0 years for men (*n* = 7226, *SD* = 10.3), and 43.0 years for accompanied solos (*n* = 1426, *SD* = 10.0). In the events belonging to the strict selection, the median age of women was 42.5 years (*n* = 3635, *SD* = 9.5), that of men was 43.0 years (*n* = 4984, *SD* = 10.2), and that of accompanied solos was 43.0 years (*n* = 965, *SD* = 9.9). Therefore, apart from the number of cases, a difference in the median age of men of 1.0, and differences in the standard deviation of up to 0.3, the two selection methods did not lead to any differences.

In the adjusted total selection, kurtosis for the age of women was − 0.510, − 0.614 for the age of men, and − 0.421 for the age of accompanied solos. Skewness amounted to 0.044 for the age of women, 0.096 for the age of men, and 0.161 for accompanied solos. The kurtosis amounted to 2.725 for the age difference within couples, paired with a skewness of 0.759, pointing to a heavier tail distribution with a slight tendency toward larger age differences where the man is older.

Percentiles were calculated of the age of women and of men admitted to swinger events on the basis of the adjusted total selection, as well as of the age difference in couples in that selection, each on the basis of weighted averages, thus recognizing bias which could be caused by the reported kurtosis and skewness.

**Fig. 7:**
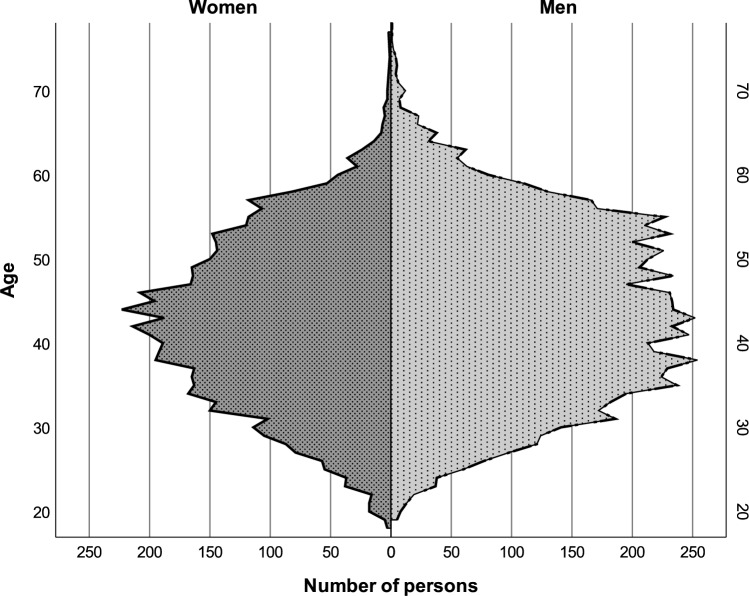
Age distribution of attendees with known age and gender admitted to events based on the adjusted total selection (*N* = 12,582; women: *n* = 5456; men: *n* = 7226). The adjusted total selection includes swinger events listed on Joyclub that occurred on three Saturdays (September 16, 23, and October 7, 2023) in Germany, where sexual activity was permitted, with at least 95 registered guests, recommended or quality-checked by Joyclub, and less than 20% external guest registrations

The results are represented in Table 2 and visualized in Fig. 7.

**Table 2:** Persons admitted to swinger events: Percentiles of ages and age differences in couples

Age value	Years	95% CI^a^	
		LL	UL
*Age of women*			
25th percentile	35	35	36
75th percentile	50	49	50
*Age of men*			
25th percentile	36	36	36
75th percentile	52	52	52
*Age difference in couples*^*b*^			
25th percentile	–5	–5	–4
75th percentile	14	13	15

CI = confidence interval; LL = lower limit; UL = upper limit

^a^10,000 bootstrap samples have shown standard errors between 0.00 and 0.50 across the percentiles, and Tukey’s hinges calculations led to the same results

^b^Positive values indicate that the man is older

#### Age-Related Distribution of Event Admissions Among Solo and Coupled Attendees

**Fig. 8:**
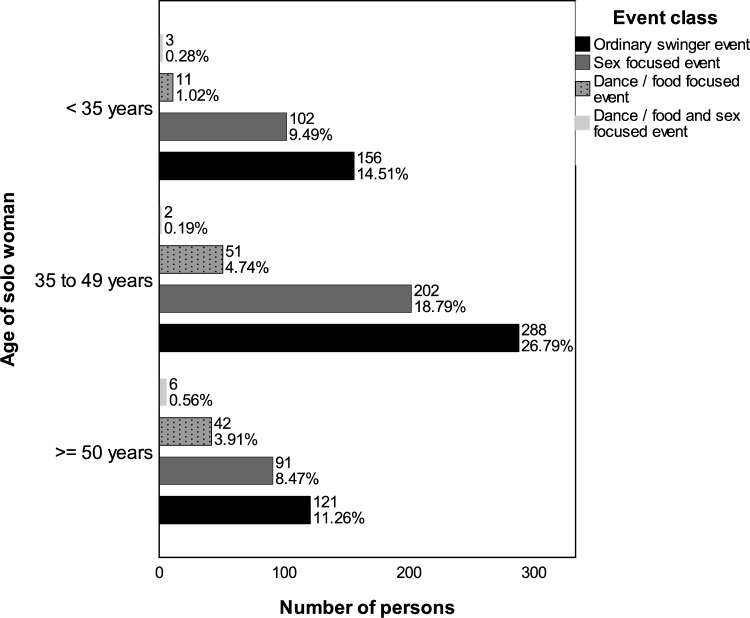
Admissions of solo women across different age groups and event categories based on the adjusted total selection (*n* = 1075). Numbers indicate the count of individuals; percentages reflect their proportion relative to all persons depicted. The adjusted total selection is described in the caption of Fig. 7

Figure 8 presents the types of events to which solo women across different age quartiles were admitted. For solo women under 35 years and those in the middle age quartile, a preference for ordinary swinger and sex-focused events is suggested by their admissions. In contrast, the admissions of women in the upper age quartile (50 years and older) lean more toward food and dance-oriented events.

**Fig. 9:**
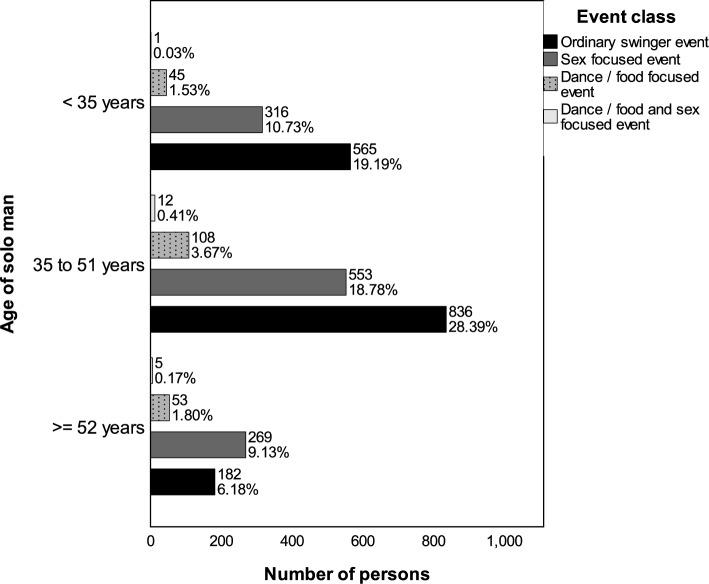
Admissions of solo men across different age groups and event categories based on the adjusted total selection (*n* = 2945). Numbers indicate the count of individuals; percentages reflect their proportion relative to all persons depicted. The adjusted total selection is described in the caption of Fig. 7

Figure 9 shows the admission trends for solo men. In the lower (under 35 years) and middle age quartiles, their admissions mirror those of solo women, predominantly to ordinary swinger and sex-focused events. For men in the upper quartile (52 years and above), a significant portion of more than half of their admissions relates to these event types, indicating different event prevalences compared to similarly aged solo women.

**Fig. 10:**
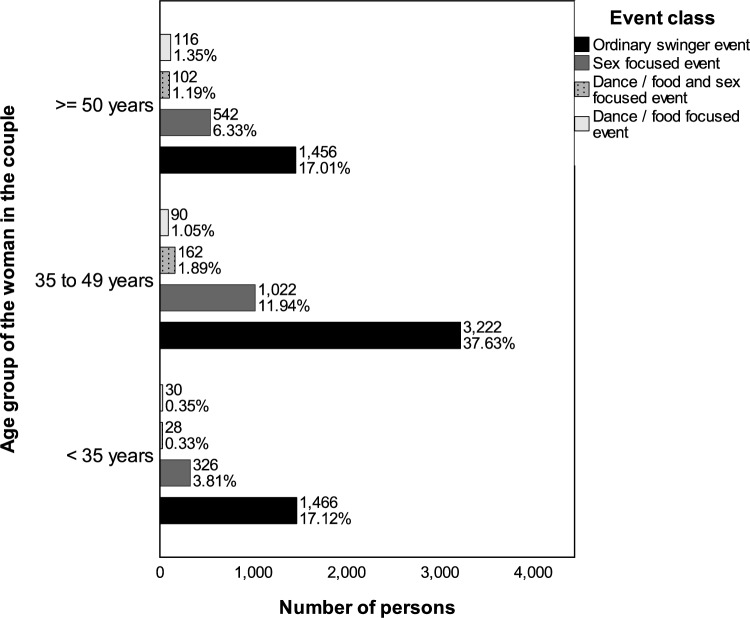
Admissions of persons in couples across different age groups—determined by the woman’s age—and event categories based on the adjusted total selection (*n* = 8562). Numbers indicate the count of individuals; percentages reflect their proportion relative to all persons depicted. The adjusted total selection is described in the caption of Fig. 7

Figure 10 depicts the admission patterns within couples across various age groups. Consistently, admissions for couples, irrespective of age, are mostly to ordinary swinger and sex-focused events. While there is a slight increase in admissions to food and dance-focused events and combined events with increasing age, these never exceed a small share of all events.

In 19 of the 53 events of the strict selection, the median age of the women was higher than the median age of the men. An event on October 7, 2023, in a venue called Quicky Rhein-Neckar, in Weinheim, Baden-Württemberg, reached by far the highest value with a median age difference of 9.0 (300 men, 108 women, 34 accompanied solos, *M* = 6.3, *SD* = 0.78). According to the description of that party, the event was specifically targeted to women who like sex with younger men. A reduced admission price of €2.00 per year of age was provided for men up to 40 years of age (the entry fee for older men was €120.00). The median age of men at that event was 35.5 years and that of women 41.8 years. The median age difference for registered couples at this event was 2.0 years, and the men were older (*n* = 65, *SD* = 5.4). Ranking second among events where at least 95% of eligible participants were registered through Joyclub and not externally, were two events held at porn cinemas equipped with play facilities. These events, which took place on October 7, 2023, showed a median age difference of 4.0 years, with women being older, among registered participants (EL BRASI Sex Film Club in Bochum: 106 men, 27 women, 6 accompanied solos, *M* = 4.1, *SD* = –2.34; XtraJOY in Augsburg: 65 men, 24 women, 4 accompanied solos, *M* = 4.1, *SD* = –2.86).

The median age of admitted participants, when considering strict selection, was lower for events admitting only couples and solo women (*n* = 14), at 40.8 years, compared with 44.0 years for events without gender-based admission restrictions (*n* = 38) and for the one event to which only couples were admitted (*Mdn* = 45.0). Events that had an age restriction of a maximum of 45 years (*n* = 5) had a significantly lower median age for women (*Mdn* = 34.0) and for men (*Mdn* = 36.0), with a very narrow range (from 31 to 35 years for women and from 34 to 38 years for men), and a median of 94.12% of admitted attendees were couples (ranging from 66.67% to 97.47%).

Detailed event data are contained in the data package for this study (Maor, 2025b).

#### Price Sensitivity of Admitted Visitors

Considering all events for which data were gathered, excluding those not charging an entrance fee for couples, the average payment for couples, inclusive of external admissions and accompanied solos, was calculated to be €102.89 assuming full price payment. Factoring in potential Joyclub Premium discounts, the minimum average payment could be as low as €94.11. This scenario presumes that all eligible admitted couples avail themselves of the discount, implying that all couples admitted through Joyclub are Premium members. External admissions were assumed to pay the full price, as Premium discounts necessitate Joyclub registration. Utilizing the same methodology, the average payment for solo men ranged from €83.14, under maximal utilization of Joyclub Premium discounts, to €89.90 without any discounts. For solo women, the figures were €30.16 with discounts and €34.37 without discounts. Detailed calculations are provided in the data package for this study (Maor, 2025b).

A bivariate Spearman correlation analysis performed on the events in the adjusted total selection where solo women were admitted (*n* = 75) showed no significant correlation between the proportion of solo women at events and the admission price applicable to them (*p* = 0.152). At events where solo men were admitted (*n* = 55), the proportion of solo men in the visitors of the respective events correlated strongly and negatively with the admission price applied to themselves (*r*_s_(54) =  − 0.356, *p* = 0.008, 95% CI[− 0.604, − 0.081]). Bootstrap biases were invariably close to zero, indicating low systematic bias. The sample sizes met the minimum requirement to achieve a statistical power of ≥ 80%.

Further two-tailed Spearman correlation analyses performed on the strict as well as the total adjusted selection, based on admission cases, showed no significant correlation between the entrance price for couples and the age of admitted women in couples (*p* = 0.561), the age of admitted men in couples (*p* = 0.948), and the age of accompanied solos (*p* = 0.360; values reported on the basis of the total adjusted selection).

#### Catchment Area of Events

**Fig. 11:**
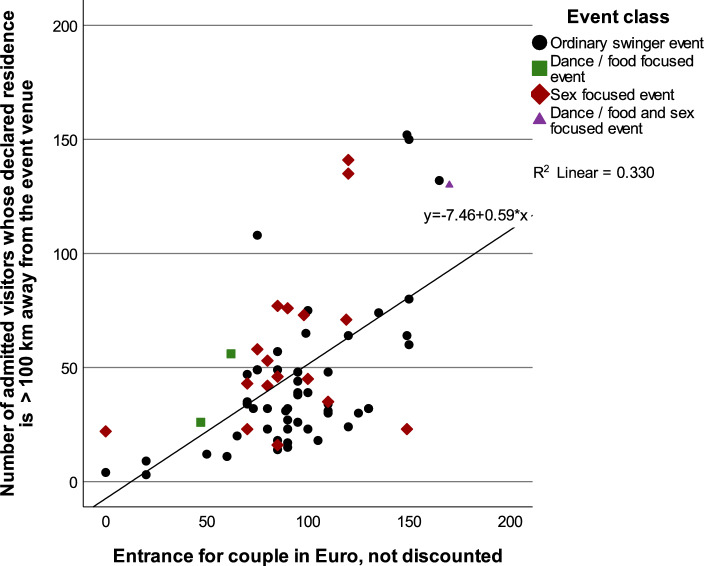
Share of long-distance visitors (admitted attendees residing more than 100 km from the event venue) in relation to the undiscounted admission price for couples, based on the adjusted total selection of events. The adjusted total selection is described in the caption of Fig. 7

The event category with the highest proportion of participants registered via Joyclub, whose stated place of residence is located more than 100 km from the event venue (*long-distance visitors*), is category 3 (combination of Food/dance-focused and Sex-focused event), with 37.1% (130 of 351 participants). This is followed by Sex-focused events with 22.4% (979 of 4,363 participants) and Ordinary swinger events with 21.9% (2,203 of 10,060 participants). Food- and dance-focused events had by far the lowest percentage at 12.4% (82 of 660 participants). The proportion of long-distance visitors registered for BDSM-focused events amounted to 21.4% (477 of 2,200 participants). The travel commitment for BDSM events was similar to that for ordinary sex events, with 21.7% traveling more than 100 km (compared to 22.43% for Ordinary swinger events), suggesting comparable levels of dedication. As visualized in Fig. 11, the share of long-distance visitors correlates with the undiscounted entrance price for couples with *r*_s_(70) = 0.518, *p* < 0.001, 95%CI[0.29, 0.69]).

For accompanied couples, both participants were assigned to the place of residence of the registered person.

### Discussion

The median ages of 42.5 years for women and 44.0 years for men, observed in the swinger events, align closely with the median ages of 44.0 years for women and 46.0 years for men identified in the first substudy. This consistency with the results of Study 1 suggests a relatively stable age demographic in these independently harvested datasets. The slightly younger median age in events, compared to the overall swinger population, could indicate a specific attraction or accessibility of these events to younger participants. When comparing these findings with international studies, such as Haywood (2022) in the UK, similarities in age demographics emerge, pointing toward a potentially universal age trend in swinger communities, independent of cultural differences.

A notable limitation arises from the unknown gender of accompanied solos, as well as the age of their respective companions. In the adjusted total selection, these individuals constitute 17.7% of the total admitted visitors. Given that the median age of accompanied solos lies between the median ages of admitted men and women, and in the absence of any indication to the contrary, it appears unlikely that their inclusion would significantly impact the overall results. Nevertheless, to preserve a formal 95% confidence level in individual observations, accompanied solos were excluded from the analysis.

#### Preferences for Event Types

The data reveal a clear preference for sex-focused events, which might reflect the core interests of the swinger community. Nevertheless, the presence of events that emphasize dance and food suggests a social aspect that goes beyond mere sexual encounters, in particular for the older members of the community; it must be emphasized that the complete set of events only included parties where on-site sex was permitted. The variety in events confirmed the descriptions provided by Venn (2015), which formed the basis of the categorization in this study. This diversity highlights the desire for experiences that cater to both social and sexual needs. The relative popularity of events with specific themes or activities could be explored further to understand nuances in sexual preferences and possibilities to integrate them into forms of playful social behavior, similar to the contributions by Grasmo and Stenross (2022) and Paasonen (2018) in other contexts.

#### Pricing Strategies and Their Impact on Solo Women’s Admissions in Swinger Events

Price sensitivity within the community is generally minimal, except among solo men. This trend may be due to their substantially higher entrance fees, which, on a weighted average, are more than double those of solo women and nearly equivalent to the admission cost for couples. This raises a speculative inquiry: could the assumed strategy of lower pricing to attract women, purportedly employed by some organizers, prove effective, or might a more desired gender distribution be better achieved through innovative preadmission strategies rather than gender-based pricing?

In contrast to the “unicorn” label assigned to unattached women in swinger communities in the UK (Haywood, 2022) and the USA (Bergstrand & Sinski, 2010, p. 62), solo women constitute a substantial proportion of admitted guests in German swinger clubs. This participation pattern is divergent from the gender compositions observed in other regions.

#### Events Organized Around the Desires of Swinger Women

At 19 different events, the observed median age of admitted women was higher than that of admitted men. In particular at an event that offered reduced entrance fees to younger men, there was a substantial alignment with women’s preferences for younger men as sexual partners. Specifically, at “Beach Club St. Tropez,” 62 women and 171 men were admitted to an event uniquely designed to cater to women’s expressed sexual desires. Here, women detailed their preferences on a paper form before being blindfolded, creating an environment where the attending men were tasked with fulfilling these expressed desires without direct visual interaction by the woman. Similarly, “Steinenhaus” admitted 118 women and 185 men to an event focused on women’s interest in men with varied skin pigmentation, demonstrating a specific response to women’s aesthetic preferences.

These events highlight aspects of swinger events where women’s desires and choices are explicitly catered to. This approach contrasts with the notion, as discussed by Vaillancourt (2006, pp. 107–108) and described as controversial by Jenks (1998), that male desires are the primary driving force behind couples’ participation in the swinger lifestyle.

For comprehensive descriptions of all events, please refer to Table S15 in the Supplementary Material.

#### Regional Variation and Travel Patterns

The study reveals that certain event categories attract participants from farther distances, particularly those combining food/dance and sexual activities, as well as sex-focused and ordinary swinger events. This finding could be indicative of either the scarcity of such events locally or a higher perceived value in these unique experiences. In contrast, a study regarding the place of origin of users of cultural offers in southern Lower Saxony found that less than 5% of attendees travel a longer distance of approximately 50 km or more (Keuchel & Graff, 2011), whereas even the lowest rates of persons traveling more than 100 km, which were found for swinger events, were considerably higher. The willingness of participants to travel significant distances for swinger events therefore suggests a dedicated and active community seeking diverse experiences, beyond what is common for social events in general.

## General Discussion

This study provides an in-depth analysis of the swinger community in Germany, focusing on several key aspects, including demographic composition, cultural factors, and gender inclusivity. The findings offer new perspectives on CNM and challenge certain presumptions about CNM derived from research on communities, particularly in North America, where a large portion of existing research has been conducted.

### Low Swinging Prevalence

The relatively low prevalence of swinging (0.21% of the population being identified swingers, i.e., self-declared swingers using a verified Joyclub profile with a photo) and generally CNM practices in Germany (0.6% of the population according to UKE, 2020) contrasts with considerably higher rates reported for North America. Despite cultural differences, monogamous relationships remain the norm in Germany, and CNM activities, including swinging, are practiced by a small minority.

Cultural differences to other world regions, particularly Germany’s permissiveness in sexual contexts, seem to contradict this discrepancy at first glance. Particularly in its eastern regions, Germany is one of the most secular areas in the world (Wolf, 2008). In consequence, non-marital cohabitation is considered acceptable and even functionally indistinguishable from marriage (Klärner & Knabe, 2017). A vast majority, 79% of West Germans and 87% of East Germans, find sex before marriage “not at all wrong” (Widmer et al., 1998, p. 351). Although a minority of only 36.2% of the respondents to the GeSiD study considered promiscuity (sex with multiple partners) acceptable (Ludwig et al., 2023), 53.8% accepted sex without love, contrasting with the findings of Grunt-Meijer and Campbell (2015) for a mixed British-Polish sample. These values appear rather low, but a comparison by Klein and Brunner (2018) between values from the Germany, the UK, the USA, and Australia shows that Germany is by far the most permissive of these countries. These attitudes may reduce the influence of religious or moral objections to CNM.

Applying the theory of reactance—which suggests that individuals are motivated to engage in prohibited behaviors when they perceive their freedoms are restricted (Brehm, 1966; Moyer-Gusé & Nabi, 2010)—the lower stigma associated with CNM in Germany may lead to less perceived restriction. Therefore, there may be less motivation to engage in CNM as a form of rebellion against societal norms. Consequently, CNM practices might not attract individuals seeking to challenge mainstream values, potentially contributing to lower participation rates and indicating that such practices remain a personal matter.

Supporting this idea, a commercial study reports that rather factors such as time constraints, personal fears, and relationship concerns influence individuals’ decisions to not engage in CNM (PE Digital, 2023). Therefore, in a society with fewer moral or religious barriers, social and psychological factors—including personal risk aversion—appear to play the significant roles in decisions against CNM arrangements. The secular and permissive nature of German society may reduce external barriers but appears not to influence such personal considerations. This hypothesis could be explored in future research.

### Age and Socioeconomic Factors

A central finding of this study is that the German swinger community is predominantly middle-aged, with median ages of identified swingers exceeding 40 years. The alignment of median ages between two independent data sources in this study supports the robustness of the methodology used. The findings contradict common assumptions that swingers are predominantly younger, consistent with other research from Europe, such as by Cerwenka et al. (2020), Haywood (2022), and O’Byrne and Watts (2011), who report similar age patterns. The results could possibly be explained by a suggestion of Klein et al. (2018), who assume that younger respondents may place greater emphasis on monogamous relationships due to their involvement in shorter, sequential partnerships, which triggers more criticism of behaviors perceived as threats to partner commitment and therefore results in lower CNM participation.

Socioeconomic factors play a role in shaping the demographic structure of the German swinger community. The correlation between the prevalence of swingers, particularly solo swingers, and population density aligns with the common understanding that non-mainstream behaviors are more prevalent in urban environments than in rural ones. Correlations between median age and income levels indicate that wealthier counties have slightly older male swingers. The corresponding positive correlation of age differences within couples with income levels suggests that affluence influences gender roles in couples. Areas with a higher proportion of technology-related jobs show a higher rate of BDSM-affiliated swingers, without this finding allowing conclusions on individual behavior patterns. The identified correlations cannot fully account for the regional differences, because unknown factors—likely linked to local cultural aspects—exert a stronger influence, illustrated by the reported similar swinger densities in nearby areas.

### BDSM, Open Relationships, and Polyamory Subgroups

The quantitative evidence of overlaps between swingers and BDSM practitioners in the German context suggests that BDSM practices are integral to the swinger community. The higher prevalence in some urban areas, similar to what is observed among swingers in general, is even more pronounced within the subgroups. The fact that BDSM-focused events attract a similar proportion of long-distance travelers as ordinary swinger events—more than typical cultural events—suggests that factors beyond event density contribute to the prevalence in major cities. This pattern is also observed in open relationship and polyamory subgroups. The willingness to travel longer distances dispels the notion that anonymity or greater permissiveness in large cities are the decisive factors. Instead, the results suggest that specific local social or cultural mentalities drive these patterns in certain cities. Cologne, with its already high density of swingers, stands out with especially pronounced values among subgroups.

The intersections among swingers, open relationship, and polyamory subgroups in Germany are shaped by varying understandings of these terms, suggesting that strategic identity presentation and cultural differences in terminology complicate demographic analysis based on self-identification. Further qualitative research could explore the underlying reasons for specific subgroup patterns.

### Limited Gender Balance

Events that explicitly target women who are interested in younger male partners or otherwise specifically explicitly focus on fetishes of the visiting women (see Table S15 in the Supplementary Material for details) challenge the stereotype that swinging is male-centric. The admissions of solo women to events highlight a degree of participation not always found in swinger communities elsewhere, such as in the USA or the UK, where solo women are seen as rare participants, which leads to a “unicorn” myth prevalent in Anglo-American contexts (Bergstrand & Sinski, 2010; Haywood, 2022).

Nonetheless, gender balance is limited within the societal sexual context in Germany. Although, according to the commercial ElitePartner study 2024 (PE Digital, 2024), a large majority of couples consider their partnership as equal, the ElitePartner study 2023 (PE Digital, 2023) reports a significant gap in openness to CNM, with 23% of men versus 11% of women indicating readiness to accept it in their relationships. This discrepancy reflects broader patterns found in international research (e.g., Levine et al., 2018), which were also observed in this study, as summarized in Table 3. Men are more likely than women to participate in CNM activities, both online and offline, including swinging and BDSM events. Efforts are taken to create environments that are attractive for women, in particular by women-focused party programs, but solo men attendance at events still far outweighs that of solo women. Men typically pay significantly higher entrance fees at events, although the solo women’s participation rates were found not to be sensitive to prices, different to those of solo men.

**Table 3:** Participation values with regard to women and men

Item	Women	Men	Ratio
*Identified swingers (total dataset)*^a^			
Not adjusted	9325	13,648	1:1.46
Duplication adjusted^b^	8715	12,524	1:1.44
*Identified solo swingers (total dataset)*^*a*^			
Not adjusted	3248	7571	1:2.33
Duplication adjusted^b^	2638	6447	1:2.44
Identified BDSM swingers^a^	671	1180	1:1.79
Identified Open Relationship swingers^a^	392	698	1:1.78
Identified Polyamory swingers^a^	94	129	1:1.37
*Number of visitors at events*^*c*^			
Total visitors	5356	7556	1:1.41
Solo visitors	1075	2945	1:2.74
Number of visitors at BDSM events^d^	942	1258	1:1.34
Average price for solo visitors of events^c, e^	34.47	89.90	1:2.60

^a^The figures relate to the number of swingers identified by a verified Joyclub profile with a picture, self-declaring as swingers. The total dataset includes 24 counties in Germany, the sociodemographic data of which were found representative for Germany, plus Berlin, Cologne, Hamburg, and Munich

^b^This adjustment factors in that individuals may maintain both a solo profile and a couple profile, which is allowed under Joyclub’s rules, affecting 610 solo women and 1,124 solo men profiles

^c^The figures relate to the number of visitors to events of the adjusted total selection of whom the gender is known. This selection comprises 76 events with 16,159 admitted visitors, including 725 external registrants and 1,426 solo attendees’ companions. Included were swinger events listed on Joyclub that occurred on three Saturdays (September 16, September 23, and October 7, 2023) in Germany, where sexual activity was permitted, that had a minimum of 95 registered guests, that were recommended or quality-checked by Joyclub, and where external guest registrations did not comprise 20% or more

^d^The events numbered 42, 49, 56, 59, 63, 66, and 84 in Table S15 of the Supplementary Material are considered BDSM events

^e^The figures relate to the average prices paid in Euro without the application of a Joyclub Premium member discount

### Swinger Women, Sexual Agency, and Cultural Support

One possible explanation for the higher independent participation of women in swinger events in Germany compared to the figures reported from other regions could relate to the concept of sexual agency. Sexual agency is the ability to communicate and act on one’s sexual desires and preferences, but does not include the availability of necessary resources (Bay-Cheng, 2019), supportive reactions from others (cf. Fetterolf & Sanchez, 2015), or that actions will align with the agent’s best interests (cf. Vanwesenbeeck et al., 2021). For sexual agency to be beneficial, it therefore has to be flanked by education to enable informed decisions, as well as a supportive and protective environment. In Germany, sexual agency is embedded in social norms and educational practices that promote autonomy and personal choice regarding sexuality.

Comprehensive sex education in Germany emphasizes consent, equality, and respect, starting from a young age (Scharmanski & Hessling, 2022). German adolescents are exposed to a relatively open and supportive approach to sexuality. The legal age of consent in Germany is 14 years. By law, contraceptives are available for free at the point of purchase up to the age of 20 years. In 2011, 67.2% of the girls aged 12–18 made use if this option (Ziller et al., 2013). In 2018–2019, more than 45% of persons in younger age groups (up to 35) reported having had their first sexual intercourse before the age of 16 (UKE, 2020, p. 15). A 1998 survey revealed that 50% of East Germans and 42% of West Germans found sex before age 16 “only sometimes wrong” or “not wrong at all,” in contrast to an average of 21% in the 24 surveyed countries (Widmer et al., 1998, p. 351). This suggests a societal framework that also factually encourages early sexual agency. According to Klein et al. (2018), the formative years of adolescence are crucial for developing lasting social and sexual behaviors.

The broader societal norms regarding sexual permissibility may also facilitate larger women participation rates in swinger events than in other countries, but this remains a hypothesis that warrants further empirical investigation.

### The Data Are Reliable

Given the atypical design of this study, it is important to briefly discuss the reliability of the data, which has already been addressed in specific contexts throughout this work. Sociological research often relies on self-reported data, and the potential biases associated with this method are typically accepted as standard limitations in the field. A key advantage of this study is that the data were not collected specifically for research purposes, which reduces the typical biases that arise in survey-based studies. Hancock et al. (2007) found that only 1.5% of users misrepresented their age in online dating profiles, and the data used in this study were based only on profiles verified by Joyclub’s case-by-case system. The legal framework in Germany also prohibits the creation of fake profiles by platform operators, providing an additional layer of data reliability.

To verify the veracity of the datasets of profile-based Study 1 and the admission-based Study 2, in particular the consistency of demographic patterns across independent datasets, a comparative analysis of age distributions from both studies was conducted. The analysis involved visual inspection of density plots and calculation of overlap coefficients, Earth Mover’s Distance (Grauman & Darrell, 2004), and Jensen–Shannon Divergence to quantify the degree of similarity between the two distributions.

The comparison revealed substantial similarities in the age distributions of participants, as shown in Figs. S1 and S2 in the Supplementary Material. Overlap coefficients were calculated at 0.6234 for women and 0.7449 for men, indicating a high degree of demographic consistency between the two studies. The Earth Mover’s Distance and Jensen–Shannon Divergence values, which were minimal, further confirmed the similarity between the datasets, despite the slightly younger median age observed in Study 2. Further details are provided in parts B.3 to B.5 of the Supplementary Material.

The independently collected data from both studies therefore show strong structural consistency, even when compared to subgroup data gathered nearly a year later. The scale and nature of the collected data align closely with findings from other studies and show strong correlations with official sociodemographic figures. Statistical analysis indicates that duplicate profiles do not meaningfully distort the results. Although uncertainties remain, as they do with any dataset, the overall quality of these data surpasses the standards of many comparable studies. Further details on this are provided in Part B of the Supplementary Material.

## Conclusion

This research provides a comprehensive empirical analysis of the swinger community in Germany, employing data harvesting techniques to study this often hard-to-reach population. The method offers advantages, such as reducing survey biases and providing a broad dataset from Joyclub, a dominant online swinger platform ranked 66th among Germany’s top websites. Nevertheless, the approach has limitations: it relies on data from this single platform, potentially limiting representation of the entire swinger community, and the fact that findings are based on self-declared data from a specific segment at a particular time. The study focuses on event admissions without confirming attendance, excluding smaller, private events. Additionally, there is uncertainty around the unknown gender of accompanied solos and the age of their companions. Moreover, the original purpose of the creation of the data limits the ability to address more focused questions about motivations and emotional experiences.

To date, there remains a significant gap in the existing research, as the interaction between demographic factors, gender relations and event preferences in the swinger community has not yet been conclusively described in theoretical terms. This study can provide an empirical basis for corresponding advances in knowledge. Longitudinal designs and mixed-method approaches, incorporating qualitative data, could further explore issues such as personal fears and anxieties around relationship models, local cultural mentalities, and the reasons behind lower participation of women in swinging despite efforts to include them.

The findings have practical implications for several stakeholders. For researchers, they underscore the importance of considering local contexts and terminology when studying sexual behaviors and relationships. Event organizers and platform operators in the swinger scene may find the insights on travel patterns, subgroup interests, and pricing strategies useful for refining their offerings. For sexual relationship counselors, the data on women’s participation provides insights into gender dynamics, suggesting that reluctance to engage in swinging is not necessarily due to external pressures like fear of being shamed or unbalanced relationships.

This study’s strength lies in its empirical approach, offering a data-driven understanding of Germany's swinger community. It contributes to a more nuanced understanding of diverse sexual practices and highlights the complexity of human sexual and relational behaviors. Future research could build on these findings, potentially through qualitative studies examining individual motivations and experiences. The consistency between independent datasets and alignment with other research support the robustness of the results.

## Supplementary Information

Below is the link to the electronic supplementary material.

Supplementary file1 (DOC 2064 KB)

## Data Availability

Data, a data management plan, a detailed description of the data and methods used, links to the data sources, which include government data concerning the demographic and economic structure of geographic units, and a detailed legal and ethical statement are available openly on Zenodo (10.5281/zenodo.15271927 and 10.5281/zenodo.15224744). Due to legal restrictions stemming from copyright-like EU sui generis database protection, as detailed in the data package, the research corpus is available only upon request to the author, specifically for the purpose of verifying the quality of scientific research. This restriction also applies to aggregated county-level data collected in August 2024 related to the profiles of swingers who meet additional criteria, such as a stated BDSM inclination or specific relationship status, which may exhibit low occupancy figures. To mitigate the real or perceived risk of disclosure—should the information from table fields be linked to other unique data—the tables containing these aggregated values are not included in the open data packages.

## Code Availability

Software scripts are available openly on Zenodo (10.5281/zenodo.15271927 and 10.5281/zenodo.15224744).

## Declarations

### Human and Animals Rights

Considering the specific mandates of German ethical committees, the study was conducted in a manner that did not necessitate formal ethical clearance. Such clearance is not required for non-interventional studies that are based on retrieving information that lies in the public domain, that may not identify any single person, and that does not allow de-anonymization, as it is the case in this study. Individual profiles were not accessed. The operators of Joyclub have confirmed in writing that the use of the data, as conducted in this study, complies with their terms and conditions. Further details are set out in the Legal and Ethical Assessment section in part G of the Supplementary Material.
