# Supplementary material for: Swingers in Germany: Sociodemography and Event Preferences Assessed from Harvested Web Data
Source: Arch Sex Behav. 2025 Aug 1;54(7):2401–27. doi: 10.1007/s10508-025-03198-z (PMC12457489; doi:10.1007/s10508-025-03198-z)
Supplement: Supplementary file 1 — Supplementary file1 (DOC 2064 KB) [file 10508_2025_3198_MOESM1_ESM.doc]

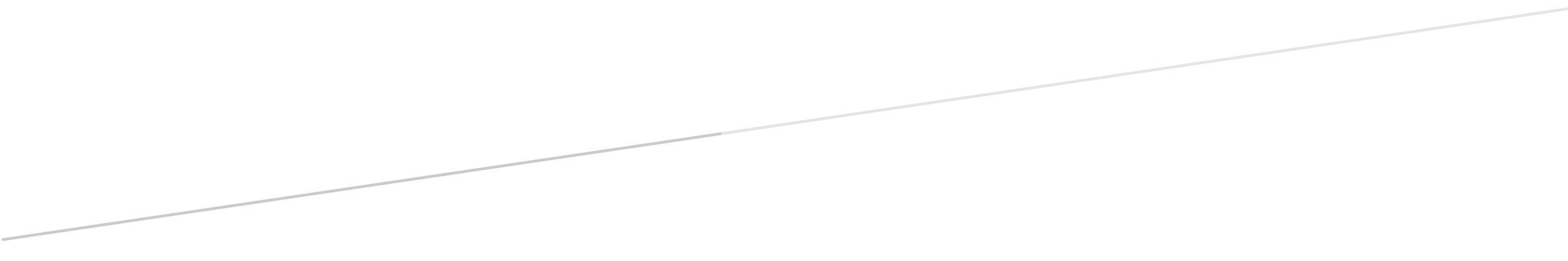


Swingers in Germany

Supplementary Material

Oliver Maor

Abstract

This is supplementary material to the study

Swingers in Germany:
Sociodemography and Event Preferences Assessed From Harvested Web Data

The study provides a comprehensive examination of the German swinger community, which is a subset of persons practicing consensual non-monogamy, using data anonymously harvested from a prominent online platform. The research aims to elucidate the demographics, social backgrounds, and behavioral patterns of swingers, thereby addressing gaps in existing literature. Two interconnected studies were conducted.

**Swingers in Germany:
Sociodemography and Event Preferences Assessed From Harvested Web Data**

**Supplementary Material**

**Year of publication:** 2025

Table of Contents

[A. Tables Related to Study 1 4](#__RefHeading___Toc196248636)

[Table S1 – Component Matrix of Principal Component Analysis for German Counties 4](#__RefHeading___Toc196248637)

[Table S2 – County Age Data 5](#__RefHeading___Toc196248638)

[Table S3 – Sociodemography of Clusters 9](#__RefHeading___Toc196248639)

[Table S4 – County Demographics 10](#__RefHeading___Toc196248640)

[Table S5 – Swinger Density 13](#__RefHeading___Toc196248641)

[Table S6 – Statistically Significant Correlations of Sociodemographic Variables in Counties 16](#__RefHeading___Toc196248642)

[Table S7 – Complete Correlation Table – Sociodemography and Swinger Variables 17](#__RefHeading___Toc196248643)

[Part A 17](#__RefHeading___Toc196248644)

[Part B 24](#__RefHeading___Toc196248645)

[Table S8: Subgroup Sizes and Age Data in Subgroups in the Representative Counties 27](#__RefHeading___Toc196248646)

[Table S9: Correlation Table – General Representative County Set and Identified BDSM Swinger Subset 29](#__RefHeading___Toc196248647)

[Table S10: Correlation Table – General Representative County Set and Identified Open Relationship Swinger Subset 34](#__RefHeading___Toc196248648)

[Table S11: Correlation Table – General Representative County Set, Identified BDSM Swinger, and Identified Open Relationship Swinger Subsets 39](#__RefHeading___Toc196248649)

[Table S12: Identified Swingers in Subgroups (Big Cities and Comparison with the Representative Counties) 42](#__RefHeading___Toc196248650)

[Table S13: Identified Swingers in Subgroups per 100,000 Inhabitants (Big Cities and Comparison with the Representative Counties) 43](#__RefHeading___Toc196248651)

[Table S14: Number of Inhabitants of the Big Cities and the Representative Counties 44](#__RefHeading___Toc196248652)

[B. Assessment of the Veracity of the Data in Study 1 45](#__RefHeading___Toc196248653)

[1. Robust Verification Process of the Data Source 45](#__RefHeading___Toc196248654)

[2. Legal and Regulatory Context 46](#__RefHeading___Toc196248655)

[3. Visual Age Data Triangulation with data from Study 2 47](#__RefHeading___Toc196248656)

[Figure S1: Age of Women Distribution Comparison 47](#__RefHeading___Toc196248657)

[Figure S2: Age of Men Distribution Comparison 48](#__RefHeading___Toc196248658)

[4. Statistical Data Triangulation with data from Study 2 48](#__RefHeading___Toc196248659)

[5. General and Region-Based Correlation Analyses with Subgroups: Age and Gender 49](#__RefHeading___Toc196248660)

[6. Analysis of Potential Profile Duplication – Couples and Solos with Duplicate Profiles of the Same Type 52](#__RefHeading___Toc196248661)

[7. Analysis of Potential Profile Duplication – Solo Profile Owners in Couple Profiles 55](#__RefHeading___Toc196248662)

[8. Methodological Considerations 61](#__RefHeading___Toc196248663)

[References Related to Part B 61](#__RefHeading___Toc196248664)

[C. Estimate of the Number of „Identified Swingers“ in Germany 64](#__RefHeading___Toc196248665)

[1. Analysis of Joyclub Profiles 64](#__RefHeading___Toc196248666)

[Joyclub Profiles of “Identified Swingers” 64](#__RefHeading___Toc196248667)

[2. Estimated Duplicates in Solo and Couple Profiles 64](#__RefHeading___Toc196248668)

[Adjusted Numbers of “Identified Swingers” 64](#__RefHeading___Toc196248669)

[3. Estimating the Prevalence of “Identified Swingers” 65](#__RefHeading___Toc196248670)

[Population Coverage of the Datasets 65](#__RefHeading___Toc196248671)

[Extrapolation to Total German Population (ages 18–75) 65](#__RefHeading___Toc196248672)

[Percentage of “Identified Swingers” in German Population (ages 18–74): 65](#__RefHeading___Toc196248673)

[D. Events and Classification 66](#__RefHeading___Toc196248674)

[Table S15: List of Events 66](#__RefHeading___Toc196248675)

[References Related to the Footnotes in Table S14 122](#__RefHeading___Toc196248676)

[References Related to the Events Data Sources (Table S14) 124](#__RefHeading___Toc196248677)

[E. Figure Related to Study 1 133](#__RefHeading___Toc196248678)

[Figure S3: Location Key – Swinger Density 133](#__RefHeading___Toc196248679)

[Key to Figure S3 133](#__RefHeading___Toc196248680)

[Lines 133](#__RefHeading___Toc196248681)

[Table S16: Key List 134](#__RefHeading___Toc196248682)

[License Related to Figure S3 135](#__RefHeading___Toc196248683)

[References Related to the Data Source and License 135](#__RefHeading___Toc196248684)

[F. Figure Related to Study 2 136](#__RefHeading___Toc196248685)

[Figure S4: Location Key – Venue Locations 136](#__RefHeading___Toc196248686)

[Key to Figure S4 137](#__RefHeading___Toc196248687)

[Lines and Stars 137](#__RefHeading___Toc196248688)

[Table S17: Key List 137](#__RefHeading___Toc196248689)

[License to Figure S4 138](#__RefHeading___Toc196248690)

[References Related to the Data Source and License 138](#__RefHeading___Toc196248691)

[G. Legal and Ethical Assessment 139](#__RefHeading___Toc196248692)

[Legality 139](#__RefHeading___Toc196248693)

[Database Protection Laws 139](#__RefHeading___Toc196248694)

[Data Protection Laws 140](#__RefHeading___Toc196248695)

[Issue of the Notification of Data Subjects 143](#__RefHeading___Toc196248696)

[Ethical Considerations beyond legal questions 143](#__RefHeading___Toc196248697)

[Possibility of Group-Based Discrimination 143](#__RefHeading___Toc196248698)

[Legality of Swinging in Germany 144](#__RefHeading___Toc196248699)

[No Danger of Job Loss 145](#__RefHeading___Toc196248700)

[No Disadvantages in Family Law 145](#__RefHeading___Toc196248701)

[No Negative Press Coverage 145](#__RefHeading___Toc196248702)

[Clubs not Operating in Secrecy 145](#__RefHeading___Toc196248703)

[Joyclub is not a Clandestine Niche Website 145](#__RefHeading___Toc196248704)

[Conclusion 146](#__RefHeading___Toc196248705)

[References Related to the Legal and Ethical Statement 146](#__RefHeading___Toc196248706)

[H. References Related to Sources of Sociodemographic County Data 149](#__RefHeading___Toc196248707)

[Overview 149](#__RefHeading___Toc196248708)

[References Related to Data Sources 149](#__RefHeading___Toc196248709)

## A. Tables Related to Study 1

## Table S1 – Component Matrix of Principal Component Analysis for German Counties

*Basis: 400 counties in Germany.*

| Variable | Component | | |
| --- | --- | --- | --- |
|  | 1 | 2 | 3 |
| Average income in Euro per capita | .092 | .767 | −.304 |
| Population density; inhabitants per km² | .800 | −.138 | −.022 |
| Total population in 100,000 | .456 | .158 | .226 |
| Population quota age 30 to 34 | .913 | −.104 | −.269 |
| Population quota age 35 to 39 | .805 | −.001 | .500 |
| Population quota age 40 to 44 | .457 | .462 | .716 |
| Population quota age 45 to 49 | .255 | .884 | .085 |
| Population quota age 50 to 54 | −.587 | .705 | −.149 |
| Population quota age 55 to 59 | −.877 | .351 | .050 |
| Population quota age 60 to 64 | −.907 | −.081 | .330 |
| Population quota age 65 to 75 | −.823 | −.334 | .374 |

*Note*. Extraction method: Principal Component Analysis. Three components were extracted, collectively explaining 80.57% of the total variance. Components with eigenvalues exceeding 1.0 were retained (Kaiser criterion). No rotation was applied.

## Table S2 – County Age Data

Age Data Related to Counties in the Representative Sample

| Official name of county | Age of woman | Age of man | Age difference in couple^a^ |
| --- | --- | --- | --- |
| Altmarkkreis Salzwedel |  |  |  |
| *Mdn* | 46.0 | 48.0 | 3.0 |
| *M* | 45.0 | 47.4 | 2.7 |
| *SD* | 9.37 | 7.84 | 5.28 |
| *n* | 28 | 58 | 26 |
| Duisburg, kreisfreie Stadt |  |  |  |
| *Mdn* | 44.0 | 47.0 | 2.0 |
| *M* | 44.0 | 46.4 | 3.6 |
| *SD* | 10.61 | 10.88 | 5.87 |
| *n* | 313 | 549 | 208 |
| Düren, Kreis |  |  |  |
| *Mdn* | 48.0 | 49.0 | 2.0 |
| *M* | 47.2 | 47.8 | 3.0 |
| *SD* | 10.01 | 11.25 | 5.80 |
| *n* | 268 | 393 | 201 |
| Eifelkreis Bitburg-Prüm |  |  |  |
| *Mdn* | 45.5 | 47.0 | 2.0 |
| *M* | 45.9 | 46.7 | 3.2 |
| *SD* | 8.94 | 9.67 | 6.47 |
| *n* | 64 | 109 | 51 |
| Frankfurt (Oder), kreisfreie Stadt |  |  |  |
| *Mdn* | 42.0 | 46.5 | 3.0 |
| *M* | 43.5 | 45.5 | 3.5 |
| *SD* | 9.91 | 9.61 | 4.36 |
| *n* | 52 | 66 | 32 |
| Fulda, Landkreis |  |  |  |
| *Mdn* | 43.0 | 46.0 | 2.0 |
| *M* | 43.6 | 46.3 | 3.1 |
| *SD* | 11.16 | 10.55 | 4.84 |
| *n* | 141 | 241 | 113 |
| Gelsenkirchen, kreisfreie Stadt |  |  |  |
| *Mdn* | 44.5 | 46.0 | 3.0 |
| *M* | 44.7 | 46.6 | 3.5 |
| *SD* | 10.71 | 10.67 | 5.25 |
| *n* | 134 | 249 | 85 |
| Grafschaft Bentheim, Landkreis |  |  |  |
| *Mdn* | 47.0 | 48.0 | 3.0 |
| *M* | 46.8 | 47.0 | 3.2 |
| *SD* | 9.34 | 11.26 | 4.81 |
| *n* | 87 | 139 | 71 |
| Halle (Saale), kreisfreie Stadt |  |  |  |
| *Mdn* | 42.0 | 43.0 | 3.0 |
| *M* | 41.5 | 43.8 | 3.2 |
| *SD* | 9.38 | 9.24 | 5.30 |
| *n* | 141 | 233 | 97 |
| Heidenheim, Landkreis |  |  |  |
| *Mdn* | 45.0 | 46.0 | 3.0 |
| *M* | 44.9 | 46.0 | 3.8 |
| *SD* | 10.12 | 10.63 | 5.59 |
| *n* | 50 | 94 | 36 |
| Hochtaunuskreis |  |  |  |
| *Mdn* | 44.0 | 49.0 | 4.0 |
| *M* | 44.6 | 48.3 | 4.2 |
| *SD* | 10.14 | 10.61 | 5.54 |
| *n* | 113 | 187 | 87 |
| Ilm-Kreis |  |  |  |
| *Mdn* | 44.5 | 45.0 | 2.0 |
| *M* | 44.5 | 45.4 | 1.8 |
| *SD* | 10.37 | 10.48 | 3.28 |
| *n* | 42 | 83 | 32 |
| Leipzig, Stadt and Landkreis |  |  |  |
| *Mdn* | 41.0 | 44.0 | 3.0 |
| *M* | 42.0 | 44.1 | 3.5 |
| *SD* | 9.86 | 10.02 | 5.52 |
| *n* | 812 | 1270 | 525 |
| Lüchow-Dannenberg, Landkreis |  |  |  |
| *Mdn* | 51.0 | 53.0 | 2.0 |
| *M* | 48.3 | 49.7 | 2.6 |
| *SD* | 12.38 | 11.13 | 4.25 |
| *n* | 24 | 31 | 19 |
| Main-Taunus-Kreis |  |  |  |
| *Mdn* | 50.0 | 51.0 | 3.0 |
| *M* | 48.2 | 49.6 | 3.0 |
| *SD* | 10.82 | 11.26 | 5.79 |
| *n* | 149 | 204 | 103 |
| Ostalbkreis, Landkreis |  |  |  |
| *Mdn* | 45.5 | 47.0 | 3.0 |
| *M* | 44.2 | 46.1 | 2.5 |
| *SD* | 10.28 | 10.48 | 4.21 |
| *n* | 60 | 201 | 25 |
| Potsdam-Mittelmark, Landkreis |  |  |  |
| *Mdn* | 47.0 | 50.0 | 2.0 |
| *M* | 48.2 | 49.7 | 2.2 |
| *SD* | 9.57 | 9.66 | 4.19 |
| *n* | 112 | 175 | 83 |
| Prignitz, Landkreis |  |  |  |
| *Mdn* | 44.0 | 46.0 | 2.0 |
| *M* | 46.6 | 47.6 | 2.1 |
| *SD* | 10.76 | 10.49 | 2.32 |
| *n* | 22 | 39 | 17 |
| Remscheid, kreisfreie Stadt |  |  |  |
| *Mdn* | 49.0 | 50.5 | 2.0 |
| *M* | 47.4 | 48.2 | 2.9 |
| *SD* | 10.10 | 12.03 | 4.88 |
| *n* | 67 | 112 | 48 |
| Schleswig-Flensburg, Landkreis |  |  |  |
| *Mdn* | 46.0 | 48.0 | 3.0 |
| *M* | 46.9 | 48.1 | 3.6 |
| *SD* | 10.34 | 10.64 | 4.59 |
| *n* | 123 | 180 | 91 |
| Sömmerda, Kreis |  |  |  |
| *Mdn* | 46.0 | 47.0 | 1.0 |
| *M* | 46.8 | 48.3 | 1.6 |
| *SD* | 9.70 | 9.85 | 7.42 |
| *n* | 28 | 49 | 24 |
| Starnberg, Landkreis |  |  |  |
| *Mdn* | 44.0 | 49.0 | 5.0 |
| *M* | 45.1 | 48.6 | 5.1 |
| *SD* | 9.88 | 10.88 | 3.56 |
| *n* | 33 | 79 | 28 |
| Traunstein, Landkreis |  |  |  |
| *Mdn* | 46.0 | 50.0 | 3.0 |
| *M* | 45.4 | 48.6 | 4.2 |
| *SD* | 9.54 | 10.83 | 5.13 |
| *n* | 110 | 194 | 87 |
| Ulm, Stadtkreis |  |  |  |
| *Mdn* | 44.0 | 44.0 | 3.0 |
| *M* | 44.4 | 45.3 | 3.3 |
| *SD* | 10.06 | 10.58 | 5.25 |
| *n* | 162 | 321 | 129 |
| ^a^Missing if not a couple. | | | |

## Table S3 – Sociodemography of Clusters

| *Sociodemographic Data of Clusters* | | | | | | |
| --- | --- | --- | --- | --- | --- | --- |
| Cluster | Population of cluster | Population density; inhabitants per km² | Average disposable income in Euro | Proportion of population with a migration background in % | Ratio of tertiary occupations | Ratio of MINT occupations |
| Ordinary wealthier county | 1,762,805 | 426.22 | 24,789 | 28.94 | 58.41 | 4.43 |
| Rural to semi-urban | 1,799,365 | 289.19 | 21,224 | 17.11 | 59.26 | 3.52 |
| Low-wealth industrial-type city | 1,004,420 | 2,130.43 | 18,386 | 33.70 | 62.18 | 3.80 |
| Wealthy suburban county | 376,235 | 387.83 | 34,722 | 37.23 | 71.81 | 6.57 |

## Table S4 – County Demographics

County Structural Data

| Official name of county | Absolute number of population of County | Population Density - inhabitants per km² | Average available income of county in Euro | Proportion of inhabitants with a migration background in % | Quota of persons employed in the tertiary sector in % | Quota of persons employed in the MINT sector in % | Divorces per 100,000 inhabitants in 2021 |
| --- | --- | --- | --- | --- | --- | --- | --- |
| **Cluster 1. Ordinary wealthier county** | | | | | | | |
| Fulda, Landkreis | 227,456 | 162 | 23,782 | 24.88 | 55.83 | 2.50 | 141.57 |
| Heidenheim, Landkreis | 132,958 | 212 | 23,718 | 36.42 | 47.37 | 2.28 | 158.48 |
| Main-Taunus-Kreis | 239,276 | 1,075 | 25,404 | 40.80 | 74.59 | 9.99 | 93.23 |
| Ostalbkreis, Landkreis | 315,009 | 208 | 25,425 | 36.42 | 52.50 | 3.58 | 155.18 |
| Potsdam-Mittelmark, Landkreis | 222,570 | 85 | 24,705 | 6.54 | 55.26 | 2.89 | 151.41 |
| Remscheid, kreisfreie Stadt | 111,770 | 1,500 | 23,768 | 40.00 | 53.21 | 2.36 | 165.17 |
| Schleswig-Flensburg, Landkreis | 206,038 | 98 | 23,506 | 11.67 | 61.49 | 1.31 | 218.41 |
| Traunstein, Landkreis | 180,779 | 116 | 25,373 | 30.99 | 54.98 | 3.95 | 149.35 |
| Ulm, Stadtkreis | 126,949 | 1,070 | 27,268 | 42.10 | 62.23 | 6.78 | 105.49 |
| **Cluster 2. Rural to semi-urban** | | | | | | | |
| Altmarkkreis Salzwedel | 81,986 | 36 | 21,702 | 4.87 | 52.70 | 1.88 | 137.04 |
| Düren, Kreis | 270,833 | 283 | 22,265 | 22.95 | 54.85 | 5.28 | 206.40 |
| Eifelkreis Bitburg-Prüm | 100,959 | 62 | 22,525 | 21.64 | 55.34 | 1.60 | 189.11 |
| Frankfurt (Oder), kreisfreie Stadt | 58,230 | 383 | 20,028 | 22.80 | 68.10 | 1.94 | 137.39 |
| Grafschaft Bentheim, Landkreis | 141,269 | 141 | 21,419 | 27.61 | 53.20 | 1.82 | 167.06 |
| Ilm-Kreis | 106,776 | 131 | 20,346 | 7.75 | 48.66 | 3.59 | 162.02 |
| Leipzig Stadt and Landkreis | 845,620 | 435 | 20,870 | 16.80 | 52.70 | 1.88 | 137.04 |
| Lüchow-Dannenberg, Landkreis | 48,472 | 39 | 22,032 | 16.20 | 59.30 | 1.72 | 140.31 |
| Prignitz, Landkreis | 75,574 | 35 | 21,130 | 5.30 | 56.96 | 1.63 | 115.72 |
| Sömmerda, Kreis | 69,646 | 85 | 20,525 | 7.75 | 44.72 | 1.65 | 155.07 |
| **Cluster 3. Low-wealth industrial-type city** | | | | | | | |
| Duisburg, kreisfreie Stadt | 502,211 | 2,127 | 18,362 | 40.37 | 57.39 | 3.48 | 182.79 |
| Gelsenkirchen, kreisfreie Stadt | 260,126 | 2,479 | 17,635 | 39.92 | 61.90 | 4.27 | 185.93 |
| Halle (Saale), kreisfreie Stadt | 242,083 | 1,763 | 19,244 | 13.19 | 71.01 | 3.97 | 192.50 |
| **Cluster 4. Wealthy suburban county** | | | | | | | |
| Hochtaunuskreis | 239,488 | 492 | 33,600 | 40.80 | 75.96 | 6.57 | 187.48 |
| Starnberg, Landkreis | 136,747 | 280 | 36,686 | 30.99 | 64.21 | 6.58 | 151.31 |

## Table S5 – Swinger Density

*Density of Identified Swingers in Counties and Clusters*

| County | Total | Women | |  | Men | |
| --- | --- | --- | --- | --- | --- | --- |
|  |  | All | Solo |  | All | Solo |
| **City with more than one million inhabitants** | | | | | | |
| Berlin | 106.2 | 48.8 | 21.3 |  | 57.4 | 29.8 |
| Hamburg | 188.1 | 80.1 | 31.9 |  | 108.0 | 59.8 |
| Köln, kreisfreie Stadt | 298.6 | 113.6 | 36.7 |  | 185.0 | 108.1 |
| München, Landeshauptstadt and Landkreis | 202.6 | 85.6 | 28.0 |  | 117.0 | 59.4 |
| *Cluster* | *168.4* | *71.5* | *26.9* |  | *96.9* | *52.3* |
| **Cluster 1. Ordinary wealthier county** | | | | | | |
| Fulda, Landkreis | 167.9 | 62.0 | 12.3 |  | 106.0 | 56.3 |
| Heidenheim, Landkreis | 108.3 | 37.6 | 10.5 |  | 70.7 | 43.6 |
| Main-Taunus-Kreis | 147.5 | 62.3 | 19.2 |  | 85.3 | 42.2 |
| Ostalbkreis, Landkreis | 82.9 | 19.0 | 11.1 |  | 63.8 | 55.9 |
| Potsdam-Mittelmark, Landkreis | 128.9 | 50.3 | 13.0 |  | 78.6 | 41.3 |
| Remscheid, kreisfreie Stadt | 160.2 | 59.9 | 17.0 |  | 100.2 | 57.3 |
| Schleswig-Flensburg, Landkreis | 147.1 | 59.7 | 15.5 |  | 87.4 | 43.2 |
| Traunstein, Landkreis | 168.2 | 60.8 | 12.7 |  | 107.3 | 59.2 |
| Ulm, Stadtkreis | 380.5 | 127.6 | 26.0 |  | 252.9 | 151.2 |
| *Cluster* | *152.9* | *55.3* | *14.7* |  | *97.7* | *57.1* |
| **Cluster 2. Rural to semi-urban** | | | | | | |
| Altmarkkreis Salzwedel | 104.9 | 34.2 | 2.4 |  | 70.7 | 39.0 |
| Düren, Kreis | 244.1 | 99.0 | 24.7 |  | 145.1 | 70.9 |
| Eifelkreis Bitburg-Prüm | 171.4 | 63.4 | 12.9 |  | 108.0 | 57.4 |
| Frankfurt (Oder), kreisfreie Stadt | 202.6 | 89.3 | 34.4 |  | 113.3 | 58.4 |
| Grafschaft Bentheim, Landkreis | 160.0 | 61.6 | 11.3 |  | 98.4 | 48.1 |
| Ilm-Kreis | 117.1 | 39.3 | 9.4 |  | 77.7 | 47.8 |
| Leipzig Stadt and Landkreis | 246.2 | 96.0 | 33.9 |  | 150.2 | 88.1 |
| Lüchow-Dannenberg, Landkreis | 113.5 | 49.5 | 10.3 |  | 64.0 | 24.8 |
| Prignitz, Landkreis | 80.7 | 29.1 | 6.6 |  | 51.6 | 29.1 |
| Sömmerda, Kreis | 110.6 | 40.2 | 5.7 |  | 70.4 | 35.9 |
| *Cluster* | *203.6* | *79.3* | *23.8* |  | *124.3* | *68.9* |
| **Cluster 3. Low-wealth industrial-type city** | | | | | | |
| Duisburg, kreisfreie Stadt | 171.6 | 62.3 | 20.9 |  | 109.3 | 67.9 |
| Gelsenkirchen, kreisfreie Stadt | 147.2 | 51.5 | 18.8 |  | 95.7 | 63.0 |
| Halle (Saale), kreisfreie Stadt | 154.5 | 58.2 | 18.2 |  | 96.2 | 56.2 |
| *Cluster* | *161.2* | *58.5* | *19.7* |  | *102.7* | *63.8* |
| **Cluster 4. Wealthy suburban county** | | | | | | |
| Hochtaunuskreis | 125.3 | 47.2 | 10.9 |  | 78.1 | 41.8 |
| Starnberg, Landkreis | 81.9 | 24.1 | 3.7 |  | 57.8 | 37.3 |
| *Cluster* | *109.5* | *38.8* | *8.2* |  | *70.7* | *40.1* |
| *Note*. All values reflect identified swingers per 100,000 inhabitants. | | | | | | |

## Table S6 – Statistically Significant Correlations of Sociodemographic Variables in Counties

| *Correlations of Sociodemographic County Data* | | | | |
| --- | --- | --- | --- | --- |
| Correlated variables | *r*_s_(23) | *p* | 95% CI | |
|  |  |  | LL | UL |
| **Population Density** | | | | |
| Migration background rate | .73 | < .001 | .42 | .89 |
| Tertiary employment ratio^a^ | .41^b^ | .048 | −0.01 | .66 |
| MINT employment ratio | .66 | < .001 | .39 | .80 |
| **Migration background rate** | | | | |
| Average income^a^ | .41^b^ | .045 | −.06 | .76 |
| MINT employment ratio | .62 | .001 | .29 | .83 |
| **Tertiary employment ratio** | | | | |
| MINT employment ratio^a^ | .41 | .044 | −.09 | .72 |
| *Note*. CI = confidence interval. Confidence intervals are bias-corrected and accelerated.  ^a^ Based on data from 384 to 398 counties in Germany, varying according to data availability, Spearman correlation analyses reveal significant correlations (*p* < .001) for the respective pairings.  ^b^Although the correlation is statistically significant at *p* < .05, the bootstrapped 95% confidence interval includes zero, indicating that the correlation may not be robust. This suggests that any observed correlation should be interpreted with caution. | | | | |

## Table S7 – Complete Correlation Table – Sociodemography and Swinger Variables

*Correlations* ***–*** *Age and Density Swinger Data and Sociodemographic County Variables*

### Part A

| Value | | | Median age of female identified swingers | Median age of male identified swingers | Median age difference of couples – positive if male is older | Identified swingers per 100,000 inhabitants | Female solo swinger density per 100,000 inhabitants | Female swinger density per 100,000 inhabitants | Male solo swinger density per 100,000 inhabitants |
| --- | --- | --- | --- | --- | --- | --- | --- | --- | --- |
| **Median age of male identified swingers** | | | | | | | | | |
| *r*_s_(23) | | | .78^**^ | – |  |  |  |  |  |
| *p* | | | <.001 |  |  |  |  |  |  |
| Bootstrap^c^ | Bias | | −0.009 |  |  |  |  |  |  |
|  | *SE* | | 0.120 |  |  |  |  |  |  |
|  | BCa 95% CI | LL | .47 |  |  |  |  |  |  |
|  |  | UL | .94 |  |  |  |  |  |  |
| **Median age difference of couples – positive if male is older** | | | | | | | | | |
| *r*_s_(23) | | | −.30 | −.03 | – |  |  |  |  |
| *p* | | | .160 | .881 |  |  |  |  |  |
| Bootstrap^c^ | Bias | | 0.006 | −0.005 |  |  |  |  |  |
|  | *SE* | | 0.173 | 0.208 |  |  |  |  |  |
|  | BCa 95% CI | LL | −.60 | −.41 |  |  |  |  |  |
|  |  | UL | .07 | .39 |  |  |  |  |  |
| **Identified swingers per 100,000 inhabitants** | | | | | | | | | |
| *r*_s_(23) | | | −.18 | −.16 | −.08 | – |  |  |  |
| *p* | | | .412 | .460 | .723 |  |  |  |  |
| Bootstrap^c^ | Bias | | 0.011 | 0.007 | −0.001 |  |  |  |  |
|  | *SE* | | 0.215 | 0.199 | 0.226 |  |  |  |  |
|  | BCa 95% CI | LL | −.54 | −.51 | −.50 |  |  |  |  |
|  |  | UL | .30 | .25 | .39 |  |  |  |  |
| **Female solo swinger density per 100,000 inhabitants** | | | | | | | | | |
| *r*_s_(23) | | | −.19 | −.17 | .06 | .85^**^ | – |  |  |
| *p* | | | .380 | .414 | .771 | <.001 |  |  |  |
| Bootstrap^c^ | Bias | | 0.011 | 0.007 | 0.002 | −0.022 |  |  |  |
|  | *SE* | | 0.23 | 0.21 | 0.23 | 0.07 |  |  |  |
|  | BCa 95% CI | LL | −.61 | −.55 | −.39 | .64 |  |  |  |
|  |  | UL | .33 | .26 | .53 | .93 |  |  |  |
| **Female swinger density per 100,000 inhabitants** | | | | | | | | | |
| *r*_s_(23) | | | −.08 | −.08 | −11 | .97^**^ | .85^**^ | – |  |
| *p* | | | .708 | .696 | .618 | <.001 | <.001 |  |  |
| Bootstrap^c^ | Bias | | 0.008 | 0.005 | −0.002 | −0.011 | −0.026 |  |  |
|  | *SE* | | 0.215 | 0.201 | 0.216 | 0.028 | 0.075 |  |  |
|  | BCa 95% CI | LL | −.46 | −.46 | −.51 | .88 | .64 |  |  |
|  |  | UL | .37 | .33 | .35 | .99 | .93 |  |  |
| **Male solo swinger density per 100,000 inhabitants** | | | | | | | | | |
| *r*_s_(23) | | | −.33 | −.39 | .06 | .86^**^ | .81^**^ | .76^**^ | – |
| *p* | | | .114 | .062 | .778 | <.001 | <.001 | <.001 |  |
| Bootstrap^c^ | Bias | | 0.009 | 0.012 | −0.002 | −0.020 | −0.025 | −0.023 |  |
|  | *SE* | | 0.201 | 0.193 | 0.235 | 0.083 | 0.095 | 0.111 |  |
|  | BCa 95% CI | LL | −.66 | −.72 | −.44 | .65 | .57 | .48 |  |
|  |  | UL | .10 | .03 | .49 | .96 | .92 | .91 |  |
| **Male swinger density per 100,000 inhabitants** | | | | | | | | | |
| *r*_s_(23) | | | −.22 | −.21 | −.03 | .99^**^ | .84^**^ | .94^**^ | .89^**^ |
| *p* | | | .311 | .330 | .885 | <.001 | <.001 | <.001 | <.001 |
| Bootstrap^c^ | Bias | | 0.010 | 0.008 | 0.000 | −0.007 | −0.025 | −0.016 | −0.017 |
|  | *SE* | | 0.216 | 0.203 | 0.231 | 0.022 | 0.077 | 0.041 | 0.073 |
|  | BCa 95% CI | LL | −.59 | −.57 | −.49 | .92 | .62 | .82 | .70 |
|  |  | UL | .25 | .23 | .44 | 1.00 | .92 | .98 | .98 |
| **Population density – inhabitants per km²** | | | | | | | | | |
| *r*_s_(23) | | | −.32 | −.20 | .35 | .46^*^ | .65^**^ | .39 | .60^**^ |
| *p* | | | .124 | .354 | .093 | .023 | <.001 | .057 | .002 |
| Bootstrap^c^ | Bias | | −0.003 | −0.005 | −0.007 | −0.018 | −0.029 | −0.019 | −0.017 |
|  | *SE* | | 0.200 | 0.210 | 0.190 | 0.162 | 0.116 | 0.166 | 0.150 |
|  | BCa 95% CI | LL | −.68 | −.61 | −.07 | .09 | .33 | .03 | .24 |
|  |  | UL | .11 | .23 | .69 | .72 | .80 | .67 | .82 |
| **Average available income in Euro** | | | | | | | | | |
| *r*_s_(23) | | | .23 | .46^*^ | .35 | −.14 | −.17 | −.12 | −.18 |
| *p* | | | .283 | .025 | .093 | .511 | .426 | .582 | .391 |
| Bootstrap^c^ | Bias | | −0.012 | −0.015 | −0.011 | 0.010 | 0.019 | 0.013 | 0.014 |
|  | *SE* | | 0.209 | 0.188 | 0.208 | 0.214 | 0.228 | 0.221 | 0.220 |
|  | BCa 95% CI | LL | −.23 | .04 | −.10 | −.54 | −.56 | −.53 | −.57 |
|  |  | UL | .61 | .78 | .70 | .32 | .33 | .36 | .30 |
| **Proportion of inhabitants with a migration background in %** | | | | | | | | | |
| *r*_s_(23) | | | −.04 | .13 | .39 | .33 | .39 | .30 | .44^*^ |
| *p* | | | .863 | .556 | .057 | .113 | .063 | .148 | .029 |
| Bootstrap^c^ | Bias | | −0.014 | −0.014 | −0.016 | −0.004 | −0.009 | −0.006 | −0.006 |
|  | *SE* | | 0.206 | 0.221 | 0.190 | 0.204 | 0.204 | 0.212 | 0.185 |
|  | BCa 95% CI | LL | −.43 | −.30 | −.06 | −.10 | −.06 | −.12 | .04 |
|  |  | UL | .39 | .58 | .69 | .70 | .73 | .69 | .75 |
| **Divorces per 100,000 inhabitants in 2021** | | | | | | | | | |
| *r*_s_(23) | | | .09 | .02 | −.06 | .07 | .07 | .01 | .17 |
| *p* | | | .660 | .935 | .783 | .728 | .747 | .961 | .433 |
| Bootstrap^c^ | Bias | | −0.011 | −0.007 | 0.005 | 0.013 | 0.023 | 0.018 | 0.004 |
|  | *SE* | | 0.225 | 0.228 | 0.196 | 0.228 | 0.236 | 0.238 | 0.226 |
|  | BCa 95% CI | LL | −.35 | −.44 | −.44 | −.35 | −.34 | −.44 | −.30 |
|  |  | UL | .52 | .47 | .33 | .53 | .57 | .50 | .62 |
| **Quota of persons employed in the tertiary sector in percent** | | | | | | | | | |
| *r*_s_(23) | | | −.25 | .11 | .41^*^ | .17 | .33 | .24 | .02 |
| *p* | | | .229 | .597 | .045 | .416 | .116 | .266 | .920 |
| Bootstrap^c^ | Bias | | 0.008 | −0.008 | −0.022 | −0.001 | −0.006 | −0.009 | 0.002 |
|  | *SE* | | 0.228 | 0.228 | 0.216 | 0.207 | 0.213 | 0.207 | 0.203 |
|  | BCa 95% CI | LL | −.68 | −.34 | −.13 | −.26 | −.12 | −.20 | −.36 |
|  |  | UL | .21 | .54 | .74 | .57 | .71 | .59 | .43 |
| **Quota of persons employed in the MINT sector in percent** | | | | | | | | | |
| *r*_s_(23) | | | −.13 | .05 | .41^*^ | .16 | .27 | .11 | .29 |
| *p* | | | .553 | .833 | .045 | .445 | .200 | .613 | .169 |
| Bootstrap^c^ | Bias | | −0.007 | −0.013 | −0.012 | −0.003 | −0.003 | −0.004 | −0.002 |
|  | *SE* | | 0.204 | 0.215 | 0.188 | 0.222 | 0.212 | 0.232 | 0.221 |
|  | BCa 95% CI | LL | −.52 | −.38 | −.03 | −.30 | −.17 | −.35 | −.19 |
|  |  | UL | .26 | .45 | .72 | .57 | .66 | .55 | .69 |

### Part B

| Variable | | | Male swinger density per 100,000 inhabitants | Population density – inhabitants per km² | Average available income in Euro | Proportion of inhabitants with a migration background in % | Divorces per 100,000 inhabitants in 2021 | Quota of persons employed in the tertiary sector in percent |
| --- | --- | --- | --- | --- | --- | --- | --- | --- |
| **Population density – inhabitants per km** | | | | | | | | |
| *r*_s_(23) | | | .46^*^ | – |  |  |  |  |
| *p* | | | .025 |  |  |  |  |  |
| Bootstrap^c^ | Bias | | −0.018 |  |  |  |  |  |
|  | *SE* | | 0.160 |  |  |  |  |  |
|  | BCa 95% CI | LL | .10 |  |  |  |  |  |
|  |  | UL | .72 |  |  |  |  |  |
| **Average available income in Euro** | | | | | | | | |
| *r*_s_(23) | | | −.16 | −.07 | – |  |  |  |
| *p* | | | .463 | .737 |  |  |  |  |
| Bootstrap^c^ | Bias | | 0.011 | 0.005 |  |  |  |  |
|  | *SE* | | 0.215 | 0.244 |  |  |  |  |
|  | BCa 95% CI | LL | −.55 | −.50 |  |  |  |  |
|  |  | UL | .31 | .44 |  |  |  |  |
| **Proportion of inhabitants with a migration background in %** | | | | | | | | |
| *r*_s_(23) | | | .31 | .73^**^ | .41^*^ | – |  |  |
| *p* | | | .144 | <.001 | .045 |  |  |  |
| Bootstrap^c^ | Bias | | −0.006 | −0.026 | −0.016 |  |  |  |
|  | *SE* | | 0.197 | 0.127 | 0.212 |  |  |  |
|  | BCa 95% CI | LL | −.10 | .42 | −.06 |  |  |  |
|  |  | UL | .66 | .89 | .76 |  |  |  |
| **Divorces per 100,000 inhabitants in 2021** | | | | | | | | |
| *r*_s_(23) | | | .11 | .19 | −.20 | .04 | – |  |
| *p* | | | .619 | .365 | .342 | .853 |  |  |
| Bootstrap^c^ | Bias | | 0.009 | 0.006 | 0.011 | 0.014 |  |  |
|  | *SE* | | 0.225 | 0.222 | 0.212 | 0.247 |  |  |
|  | BCa 95% CI | LL | −.34 | −.27 | −.59 | −.41 |  |  |
|  |  | UL | .56 | .63 | .25 | .54 |  |  |
| **Quota of persons employed in the tertiary sector in percent** | | | | | | | | |
| *r*_s_(23) | | | .14 | .41^*^ | .15 | .34 | −.01 | – |
| *p* | | | .517 | .048 | .470 | .104 | .945 |  |
| Bootstrap^c^ | Bias | | −0.001 | −0.025 | −0.006 | −0.022 | 0.017 |  |
|  | *SE* | | 0.212 | 0.168 | 0.247 | 0.205 | 0.219 |  |
|  | BCa 95% CI | LL | −.29 | −.01 | −.35 | −.11 | −.45 |  |
|  |  | UL | .53 | .66 | .57 | .66 | .43 |  |
| **Quota of persons employed in the MINT sector in percent** | | | | | | | | |
| *r*_s_(23) | | | .15 | .66^**^ | .37 | .62^**^ | −.04 | .41^*^ |
| *p* | | | .485 | <.001 | .073 | .001 | .850 | .044 |
| Bootstrap^c^ | Bias | | 0.000 | −0.020 | −0.022 | −0.023 | 0.011 | −0.026 |
|  | *SE* | | 0.227 | 0.108 | 0.209 | 0.137 | 0.272 | 0.201 |
|  | BCa 95% CI | LL | −.32 | .39 | −.11 | .29 | −.51 | −.09 |
|  |  | UL | .60 | .80 | .70 | .83 | .55 | .72 |
| *Note.* Bca = bias-corrected and accelerated; CI = confidence interval; LL = lower limit; UL = upper limit. | | | | | | | | |
| c. Bootstrap results are based on 1,000 bootstrap samples. | | | | | | | | |

## Table S8: Subgroup Sizes and Age Data in Subgroups in the Representative Counties

*Age data of identified swingers (definition: see main study) in whose solo or couple Joyclub profile
the respective item “BDSM”, “Open Relationship”, or “Polyamory” was set to the affirmative.*

| Type of interest or relationship of swinger(s) | | Value | Bootstrap^a^ | | | |
| --- | --- | --- | --- | --- | --- | --- |
|  |  |  | Bias | *SE* | BCa 95% CI | |
|  |  |  |  |  | LL | UL |
| **Age of Woman** | | | | | | |
| BDSM | |  |  |  |  |  |
| *n* | | 671 |  |  |  |  |
| *Mdn* (years) | | 40.0 | 0.10 | 0.55 | 40.0 | 40.0 |
| *SD* | | 9.64 | −0.015 | 0.261 | 9.13 | 10.10 |
| Skewness | | 0.269 | 0.000 | 0.090 | 0.109 | 0.454 |
| Kurtosis | | −0.085 | −0.008 | 0.262 | -0.451 | 0.393 |
| Open Relationship | |  |  |  |  |  |
| *n* | | 392 |  |  |  |  |
| *Mdn (years)* | | 43.0 | −0.17 | 0.75 | 43.0 | 43.0 |
| *SD* | | 10.79 | −0.004 | 0.340 | 10.09 | 11.45 |
| Skewness | | 0.173 | −0.005 | 0.084 | 0.015 | 0.321 |
| Kurtosis | | −0.472 | −0.004 | 0.140 | −0.712 | −0.212 |
| Polyamory | |  |  |  |  |  |
| *n* | | 94 |  |  |  |  |
| *Mdn* (years) | | 41.0 | −0.49 | 1.01 | 40.0 | 41.0 |
| *SD* | | 9.17 | −0.087 | 0.601 | 8.07 | 10.07 |
| Skewness | | 0.365 | −0.008 | 0.148 | 0.062 | 0.622 |
| Kurtosis | | −0.293 | 0.010 | 0.287 | −0.741 | 0.303 |
| **Age of man** | | | | | | |
| BDSM | |  |  |  |  |  |
| *n* | | 1180 |  |  |  |  |
| *Mdn (years)* | | 42.0 | −0.25 | 0.47 | 41.0 | 43.0 |
| *SD* | | 10.57 | 0.007 | 0.188 | 10.17 | 10.97 |
| Skewness | | 0.173 | 0.000 | 0.054 | 0.064 | 0.283 |
| Kurtosis | | −0.551 | −0.001 | .106 | −0.718 | −0.360 |
| Open Relationship | |  |  |  |  |  |
| *n* | | 698 |  |  |  |  |
| *Mdn (years)* | | 45.0 | 0.45 | 0.58 | 44.5^b^ | 47.0 |
| *SD* | | 10.59 | −0.001 | 0.244 | 10.09^b^ | 11.05 |
| Skewness | | 0.148 | 0.000 | 0.068 | 0.013^b^ | 0.286 |
| Kurtosis | | −0.589 | 0.003 | 0.099 | −0.764^b^ | −0.371 |
| Polyamory |  |  |  |  |  |  |
| *n* | | 129 |  |  |  |  |
| *Mdn (years)* | | 42.0 | 0.15 | 1.07 | 40.0^b^ | 45.0 |
| *SD* | | 10.64 | −0.050 | 0.676 | 9.21^b^ | 11.85 |
| Skewness | | 0.804 | −0.007 | 0.180 | 0.455^b^ | 1.163 |
| Kurtosis | | 0.215 | −0.010 | 0.535 | −0.635^b^ | 1.437 |
| **Age difference in couple** | | | | | | |
| BDSM | |  |  |  |  |  |
| *n* | | 341 |  |  |  |  |
| *Mdn (years)* | | 3.0 | 0.15 | 0.45 | 3.0 | 3.0 |
| *SD* | | 6.10 | −0.044 | 0.371 | 5.44 | 6.71 |
| Skewness | | 0.939 | −0.038 | 0.276 | 0.418 | 1.345 |
| Kurtosis | | 3.076 | −0.190 | 0.892 | 1.428 | 4.296 |
| Open Relationship | |  |  |  |  |  |
| *n* | | 270 |  |  |  |  |
| *Mdn (years)* | | 3.0 | −0.11 | 0.38 | 2.0 | 3.0 |
| *SD* | | 5.88 | −0.018 | .395 | 5.16 | 6.59 |
| Skewness | | 0.778 | −0.042 | .364 | 0.025 | 1.376 |
| Kurtosis | | 3.435 | −0.198 | 1.086 | 1.394 | 4.920 |
| Polyamory |  |  |  |  |  |  |
| *n* | | 46 |  |  |  |  |
| *Mdn (years)* | | 2.0 | .39 | 1.03 | 1.0 | 4.0 |
| *SD* | | 5.74 | −0.095 | 0.679 | 4.47 | 6.81 |
| Skewness | | −0.615 | 0.108 | 0.383 | −1.236 | 0.354 |
| Kurtosis | | 0.770 | −0.309 | 0.936 | −0.808 | 1.749 |
| *Note.* Bca = bias-corrected and accelerated; CI = confidence interval; LL = lower limit; UL = upper limit. | | | | | | |
| a. Bootstrap results are based on 1,000 bootstrap samples. | | | | | | |
| b. Some results could not be computed from jackknife samples, so this confidence interval is computed by the percentile method rather than the BCa method. | | | | | | |

## Table S9: Correlation Table – General Representative County Set and Identified BDSM Swinger Subset

*Correlations (General Representative County Set and BDSM Subset for Representative Counties)*

| Value | | | Quota of persons employed in the tertiary sector in % | Quota of persons employed in the MINT sector in % | Population density – inhabitants per km² | Average available income in Euro | Median age of identified swinger women | Median age of BDSM women | Median age of identified swinger men | Median age of BDSM men | Median age difference of couples; positive if man is older |
| --- | --- | --- | --- | --- | --- | --- | --- | --- | --- | --- | --- |
| **Quota of persons employed in the MINT sector in %** | | | | | | | | | | | |
| *r*_s_(23) | | | .41^*^ | – |  |  |  |  |  |  |  |
| *p* | | | .044 |  |  |  |  |  |  |  |  |
| Bootstrap^c^ | Bias | | −0.005 |  |  |  |  |  |  |  |  |
|  | *SE* | | 0.192 |  |  |  |  |  |  |  |  |
|  | BCa 95% CI | LL | .02 |  |  |  |  |  |  |  |  |
|  |  | UL | .71 |  |  |  |  |  |  |  |  |
| **Population density – inhabitants per km²** | | | | | | | | | | | |
| *r*_s_(23) | | | .41^*^ | .66^**^ | – |  |  |  |  |  |  |
| *p* | | | .048 | <.001 |  |  |  |  |  |  |  |
| Bootstrap^c^ | Bias | | −0.017 | −0.029 |  |  |  |  |  |  |  |
|  | *SE* | | 0.162 | 0.114 |  |  |  |  |  |  |  |
|  | BCa 95% CI | LL | .03 | .45 |  |  |  |  |  |  |  |
|  |  | UL | .64 | .78 |  |  |  |  |  |  |  |
| **Average available income in Euro** | | | | | | | | | | | |
| *r*_s_(23) | | | .15 | .37 | −.07 | – |  |  |  |  |  |
| *p* | | | .470 | .073 | .737 |  |  |  |  |  |  |
| Bootstrap^c^ | Bias | | 0.011 | −0.013 | 0.001 |  |  |  |  |  |  |
|  | *SE* | | 0.251 | 0.216 | 0.247 |  |  |  |  |  |  |
|  | BCa 95% CI | LL | −.30 | −.13 | −.47 |  |  |  |  |  |  |
|  |  | UL | .65 | .70 | .39 |  |  |  |  |  |  |
| **Median age of identified swinger women** | | | | | | | | | | | |
| *r*_s_(23) | | | −.25 | −.13 | −.32 | .23 | – |  |  |  |  |
| *p* | | | .229 | .553 | .124 | .283 |  |  |  |  |  |
| Bootstrap^c^ | Bias | | 0.002 | −0.003 | 0.013 | −0.021 |  |  |  |  |  |
|  | *SE* | | 0.227 | 0.212 | 0.204 | 0.208 |  |  |  |  |  |
|  | BCa 95% CI | LL | −.65 | −.50 | −.65 | −.24 |  |  |  |  |  |
|  |  | UL | .20 | .24 | .09 | .56 |  |  |  |  |  |
| **Median age of BDSM women** | | | | | | | | | | | |
| *r*_s_(23) | | | .17 | −.18 | −.22 | .24 | .68^**^ | – |  |  |  |
| *p* | | | .423 | .391 | .309 | .252 | <.001 |  |  |  |  |
| Bootstrap^c^ | Bias | | −0.009 | 0.003 | 0.006 | −0.015 | −0.019 |  |  |  |  |
|  | *SE* | | 0.202 | 0.245 | 0.232 | 0.169 | 0.104 |  |  |  |  |
|  | BCa 95% CI | LL | −.25 | −.61 | −.64 | −.07 | .48 |  |  |  |  |
|  |  | UL | .53 | .29 | .27 | .51 | .80 |  |  |  |  |
| **Median age of identified swinger men** | | | | | | | | | | | |
| *r*_s_(23) | | | .11 | .05 | −.20 | .46^*^ | .78^**^ | .67^**^ | – |  |  |
| *p* | | | .597 | .833 | .354 | .025 | <.001 | <.001 |  |  |  |
| Bootstrap^c^ | Bias | | −0.011 | −0.008 | 0.008 | −0.015 | −0.015 | −0.028 |  |  |  |
|  | *SE* | | 0.226 | 0.225 | 0.216 | 0.184 | 0.114 | 0.113 |  |  |  |
|  | BCa 95% CI | LL | −.31 | −.39 | −.57 | .07 | .51 | .45 |  |  |  |
|  |  | UL | .48 | .47 | .24 | .75 | .93 | .80 |  |  |  |
| **Median age of BDSM men** | | | | | | | | | | | |
| *r*_s_(23) | | | .16 | .17 | −.20 | .48^*^ | .54^**^ | .58^**^ | .76^**^ | – |  |
| *p* | | | .457 | .437 | .354 | .018 | .007 | .003 | <.001 |  |  |
| Bootstrap^c^ | Bias | | 0.001 | −0.011 | 0.000 | −0.015 | −0.028 | −0.020 | −0.023 |  |  |
|  | *SE* | | 0.207 | 0.206 | 0.231 | 0.150 | 0.156 | 0.169 | 0.086 |  |  |
|  | BCa 95% CI | LL | −.25 | −.24 | −.63 | .18 | .19 | .24 | .60 |  |  |
|  |  | UL | .54 | .54 | .26 | .71 | .74 | .82 | .84 |  |  |
| **Median age difference of couples - positive if male is older** | | | | | | | | | | | |
| *r*_s_(23) | | | .41^*^ | .41^*^ | .35 | .35 | −.30 | .06 | −.03 | .05 | – |
| *p* | | | .045 | .045 | .093 | .093 | .160 | .795 | .881 | .816 |  |
| Bootstrap^c^ | Bias | | −0.005 | −0.015 | −0.024 | −0.007 | 0.008 | 0.004 | −0.002 | 0.006 |  |
|  | *SE* | | 0.193 | 0.182 | 0.183 | 0.218 | 0.178 | 0.196 | 0.206 | 0.214 |  |
|  | BCa 95% CI | LL | −.03 | .03 | −.02 | −.11 | −.60 | −.32 | −.43 | −.40 |  |
|  |  | UL | .72 | .69 | .61 | .72 | .10 | .42 | .38 | .49 |  |
| **Median age difference in BDSM couple; positive if man is older** | | | | | | | | | | | |
| *r*_s_(23) | | | −.21 | .13 | .02 | .23 | −.13 | −.29 | −.22 | .07 | .00 |
| *p* | | | .331 | .543 | .935 | .279 | .552 | .173 | .298 | .729 | .998 |
| Bootstrap^c^ | Bias | | 0.005 | −0.011 | −0.003 | −0.008 | 0.001 | 0.007 | 0.012 | −0.010 | −0.014 |
|  | *SE* | | 0.214 | 0.210 | 0.204 | 0.209 | 0.217 | 0.199 | 0.213 | 0.257 | 0.245 |
|  | BCa 95% CI | LL | −.59 | −.27 | −.37 | −.17 | −.58 | −.63 | −.63 | −.48 | −.50 |
|  |  | UL | .25 | .48 | .41 | .62 | .34 | .18 | .34 | .55 | .42 |
| Note. BCa = bias-corrected and accelerated; CI = confidence interval; LL = lower limit; UL = upper limit. | | | | | | | | | | | |
| *. Correlation is significant at the .05 level (2-tailed). | | | | | | | | | | | |
| **. Correlation is significant at the .01 level (2-tailed). | | | | | | | | | | | |
| c. Bootstrap results are based on 1,000 bootstrap samples. | | | | | | | | | | | |

## Table S10: Correlation Table – General Representative County Set and Identified Open Relationship Swinger Subset

*Correlations (General Representative County Set and Open Relationship Swinger Subset for Representative Counties)*

***Note:*** *This table is based on the data from 23 counties (in other tables: 24) because a data point is missing: in Lüchow-Dannenberg county, no profile of any Open Relationship swinger woman was identified.*

| Value | | | Quota of persons employed in the tertiary sector in % | Quota of persons employed in the MINT sector in % | Population density – inhabitants per km² | Average available income in Euro | Median age of identified swinger women | Median age of Open Relationship women | Median age of identified swinger men | Median age of Open Relationship swinger men | Median age difference of couples; positive if male is older |
| --- | --- | --- | --- | --- | --- | --- | --- | --- | --- | --- | --- |
| **Quota of persons employed in the MINT sector in %** | | | | | | | | | | | |
| *r*_s_(22) | | | .46^*^ | – |  |  |  |  |  |  |  |
| *p* | | | .026 |  |  |  |  |  |  |  |  |
| Bootstrap^c^ | Bias | | −0.019 |  |  |  |  |  |  |  |  |
|  | *SE* | | 0.187 |  |  |  |  |  |  |  |  |
|  | BCa 95% CI | LL | .07 |  |  |  |  |  |  |  |  |
|  |  | UL | .75 |  |  |  |  |  |  |  |  |
| **Population density – inhabitants per km²** | | | | | | | | | | | |
| *r*_s_(22) | | | .45^*^ | .62^**^ | – |  |  |  |  |  |  |
| *p* | | | .033 | .001 |  |  |  |  |  |  |  |
| Bootstrap^c^ | Bias | | −0.022 | −0.031 |  |  |  |  |  |  |  |
|  | *SE* | | 0.151 | 0.126 |  |  |  |  |  |  |  |
|  | BCa 95% CI | LL | .13 | .34 |  |  |  |  |  |  |  |
|  |  | UL | .65 | .77 |  |  |  |  |  |  |  |
| **Average available income in Euro** | | | | | | | | | | | |
| *r*_s_(22) | | | .18 | .38 | −.11 | – |  |  |  |  |  |
| *p* | | | .404 | .075 | .617 |  |  |  |  |  |  |
| Bootstrap^c^ | Bias | | −0.005 | −0.016 | −0.005 |  |  |  |  |  |  |
|  | *SE* | | 0.248 | 0.220 | 0.251 |  |  |  |  |  |  |
|  | BCa 95% CI | LL | −.27 | −.11 | −.59 |  |  |  |  |  |  |
|  |  | UL | .61 | .73 | .40 |  |  |  |  |  |  |
| **Median age of identified swinger women** | | | | | | | | | | | |
| *r*_s_(22) | | | −.24 | −.19 | −.41 | .19 | – |  |  |  |  |
| *p* | | | .277 | .397 | .054 | .393 |  |  |  |  |  |
| Bootstrap^c^ | Bias | | −0.005 | 0.000 | 0.006 | −0.013 |  |  |  |  |  |
|  | *SE* | | 0.226 | 0.223 | 0.201 | 0.227 |  |  |  |  |  |
|  | BCa 95% CI | LL | −.63 | −.59 | −.74 | −.28 |  |  |  |  |  |
|  |  | UL | .17 | .24 | .00 | .56 |  |  |  |  |  |
| **Median age of Open Relationship women** | | | | | | | | | | | |
| *r*_s_(22) | | | −.05 | .29 | −.21 | .41 | .56^**^ | – |  |  |  |
| *p* | | | .832 | .176 | .330 | .055 | .006 |  |  |  |  |
| Bootstrap^c^ | Bias | | 0.002 | −0.017 | 0.001 | −0.019 | −0.014 |  |  |  |  |
|  | *SE* | | 0.206 | 0.191 | 0.190 | 0.182 | 0.173 |  |  |  |  |
|  | BCa 95% CI | LL | −.47 | −.08 | −.53 | .02 | .15 |  |  |  |  |
|  |  | UL | .40 | .61 | .14 | .69 | .82 |  |  |  |  |
| **Median age of identified swinger men** | | | | | | | | | | | |
| *r*_s_(22) | | | .13 | .01 | −.28 | .43^*^ | .78^**^ | .52^*^ | – |  |  |
| *p* | | | .540 | .973 | .201 | .039 | <.001 | .012 |  |  |  |
| Bootstrap^c^ | Bias | | −0.009 | −0.007 | 0.001 | −0.010 | −0.016 | −0.008 |  |  |  |
|  | *SE* | | 0.226 | 0.226 | 0.214 | 0.188 | 0.118 | 0.162 |  |  |  |
|  | BCa 95% CI | LL | −.35 | −.43 | −.61 | .02 | .48 | .09 |  |  |  |
|  |  | UL | .49 | .38 | .11 | .74 | .93 | .80 |  |  |  |
| **Median age of Open Relationship swinger men** | | | | | | | | | | | |
| *r*_s_(22) | | | −.03 | .00 | −.14 | .37 | .48^*^ | .24 | .44^*^ | – |  |
| *p* | | | .884 | .987 | .538 | .078 | .021 | .262 | .037 |  |  |
| Bootstrap^c^ | Bias | | −0.012 | −0.012 | −0.006 | −0.028 | −0.011 | −0.011 | −0.016 |  |  |
|  | *SE* | | 0.224 | 0.232 | 0.217 | 0.187 | 0.197 | 0.236 | 0.194 |  |  |
|  | BCa 95% CI | LL | −.42 | −.40 | −.51 | −.03 | .02 | −.28 | −.01 |  |  |
|  |  | UL | .36 | .41 | .28 | .63 | .79 | .68 | .74 |  |  |
| **Median age difference of couples - positive if male is older** | | | | | | | | | | | |
| *r*_s_(22) | | | .43^*^ | .38 | .30 | .34 | −.35 | −.05 | −.07 | −.23 | – |
| *p* | | | .040 | .072 | .165 | .108 | .102 | .838 | .753 | .287 |  |
| Bootstrap^c^ | Bias | | −0.012 | −0.006 | −0.009 | −0.014 | 0.004 | 0.002 | −0.001 | −0.003 |  |
|  | *SE* | | 0.194 | 0.186 | 0.198 | 0.211 | 0.168 | 0.214 | 0.200 | 0.199 |  |
|  | BCa 95% CI | LL | .01 | .00 | −.15 | −.11 | −.61 | −.43 | −.41 | −.60 |  |
|  |  | UL | .74 | .68 | .65 | .67 | −.03 | .36 | .27 | .15 |  |
| **Median age difference in Open Relationship couple; positive if man is older** | | | | | | | | | | | |
| *r*_s_(22) | | | .03 | −.17 | .01 | .26 | .08 | −.18 | .09 | .37 | .06 |
| *p* | | | .905 | .447 | .955 | .224 | .705 | .409 | .684 | .078 | .798 |
| Bootstrap^c^ | Bias | | 0.003 | 0.013 | 0.003 | −0.007 | 0.000 | 0.013 | 0.001 | −0.013 | −0.001 |
|  | *SE* | | 0.212 | 0.234 | 0.223 | 0.215 | 0.213 | 0.222 | 0.212 | 0.218 | 0.237 |
|  | BCa 95% CI | LL | −.37 | −.58 | −.44 | −.21 | −.39 | −.59 | −.34 | −.08 | −.41 |
|  |  | UL | .42 | .32 | .46 | .64 | .50 | .32 | .48 | .72 | .49 |
| Note. BCa = bias-corrected and accelerated; CI = confidence interval; LL = lower limit; UL = upper limit. | | | | | | | | | | | |
| *. Correlation is significant at the .05 level (2-tailed). | | | | | | | | | | | |
| **. Correlation is significant at the .01 level (2-tailed). | | | | | | | | | | | |
| c. Bootstrap results are based on 1,000 bootstrap samples. | | | | | | | | | | | |

## Table S11: Correlation Table – General Representative County Set, Identified BDSM Swinger, and Identified Open Relationship Swinger Subsets

Correlations

| Variable | | | Quota of persons employed in the MINT sector in % | Quota of persons employed in the tertiary sector in % | Population density – inhabitants per km² | Average available income in Euro | Identified swingers per 100,000 inhabitants | BDSM swingers per 100,000 inhabitants |
| --- | --- | --- | --- | --- | --- | --- | --- | --- |
| **Quota of persons employed in the tertiary sector in %** | | | | | | | | |
| *r*_s_(23) | | | .41^*^ | – |  |  |  |  |
| *p* | | | .044 |  |  |  |  |  |
| Bootstrap^c^ | Bias | | −0.01 |  |  |  |  |  |
|  | *SE* | | 0.19 |  |  |  |  |  |
|  | BCa 95% CI | LL | −.08 |  |  |  |  |  |
|  |  | UL | .76 |  |  |  |  |  |
| **Population density – inhabitants per km²** | | | | | | | | |
| *r*_s_(23) | | | .66^**^ | .41^*^ | – |  |  |  |
| *p* | | | <.001 | .048 |  |  |  |  |
| Bootstrap^c^ | Bias | | −0.02 | −0.01 |  |  |  |  |
|  | *SE* | | 0.11 | 0.16 |  |  |  |  |
|  | BCa 95% CI | LL | .43 | .03 |  |  |  |  |
|  |  | UL | .79 | .66 |  |  |  |  |
| **Average available income in Euro** | | | | | | | | |
| *r*_s_(23) | | | .37 | .15 | −.07 | – |  |  |
| *p* | | | .073 | .470 | .737 |  |  |  |
| Bootstrap^c^ | Bias | | −0.01 | 0.01 | 0.01 |  |  |  |
|  | *SE* | | 0.21 | 0.24 | 0.24 |  |  |  |
|  | BCa 95% CI | LL | −.05 | −.33 | −.49 |  |  |  |
|  |  | UL | .70 | .64 | .41 |  |  |  |
| **Identified swingers per 100,000 inhabitants** | | | | | | | | |
| *r*_s_(23) | | | .16 | .17 | .46^*^ | −.14 | – |  |
| *p* | | | .445 | .416 | .023 | .511 |  |  |
| Bootstrap^c^ | Bias | | −0.011 | 0.002 | −0.024 | 0.016 |  |  |
|  | *SE* | | 0.221 | 0.204 | 0.160 | 0.216 |  |  |
|  | BCa 95% CI | LL | −.27 | −.23 | .14 | −.56 |  |  |
|  |  | UL | .56 | .57 | .68 | .32 |  |  |
| **BDSM swingers per 100,000 inhabitants** | | | | | | | | |
| *r*_s_(23) | | | .17 | .33 | .31 | .22 | .43^*^ | – |
| *p* | | | .433 | .110 | .144 | .312 | .037 |  |
| Bootstrap^c^ | Bias | | 0.00 | −0.01 | −0.02 | 0.00 | −0.01 |  |
|  | *SE* | | 0.23 | 0.18 | 0.21 | 0.18 | 0.20 |  |
|  | BCa 95% CI | LL | −.28 | −.05 | −.09 | −.14 | −.05 |  |
|  |  | UL | .61 | .65 | .62 | .57 | .78 |  |
| **Open Relationship swingers per 100,000 inhabitants** | | | | | | | | |
| *r*_s_(23) | | | .59^**^ | .22 | .63^**^ | .12 | .46^*^ | .65^**^ |
| *p* | | | .003 | .304 | .001 | .576 | .023 | <.001 |
| Bootstrap^c^ | Bias | | −0.02 | 0.01 | −0.02 | 0.00 | −0.02 | −0.02 |
|  | *SE* | | 0.14 | 0.19 | 0.16 | 0.23 | 0.15 | 0.15 |
|  | BCa 95% CI | LL | .28 | −.20 | .28 | .30 | −.04 | .30 |
|  |  | UL | .81 | .62 | .86 | .86 | .81 | .86 |
| *Note*. Bca = bias-corrected and accelerated; ; CI = confidence interval; LL = lower limit; UL = upper limit. | | | | | | | | |
| *. Correlation is significant at the .05 level (2-tailed). | | | | | | | | |
| **. Correlation is significant at the .01 level (2-tailed). | | | | | | | | |
| c. Bootstrap results are based on 1,000 bootstrap samples. . | | | | | | | | |

## Table S12: Identified Swingers in Subgroups (Big Cities and Comparison with the Representative Counties)

Number of persons

| City | Open Relationship | | |  | Open Relationship | | |  | Polyamory | | |
| --- | --- | --- | --- | --- | --- | --- | --- | --- | --- | --- | --- |
|  | Total | Solo women | Solo men |  | Total | Solo women | Solo men |  | Total | Solo women | Solo men |
| Berlin | 2,028 | 318 | 1,038 |  | 1,323 | 143 | 580 |  | 331 | 57 | 140 |
| Hamburg | 1201 | 191 | 620 |  | 724 | 83 | 323 |  | 161 | 32 | 63 |
| Cologne | 838 | 108 | 460 |  | 499 | 55 | 208 |  | 93 | 22 | 35 |
| Munich^a^ | 1415 | 192 | 799 |  | 789 | 86 | 317 |  | 155 | 22 | 61 |
| Total | 5,482 | 809 | 2,917 |  | 3,335 | 367 | 1,428 |  | 740 | 133 | 299 |
| *Representative counties* | 1,851 | 330 | 839 |  | 1,090 | 122 | 428 |  | 223 | 48 | 83 |

a. “Munich” relates to the combined city and *Landkreis* (surrounding county).

## Table S13: Identified Swingers in Subgroups per 100,000 Inhabitants (Big Cities and Comparison with the Representative Counties)

Number of persons per 100,000 inhabitants

| City | Open Relationship | | |  | Open Relationship | | |  | Polyamory | | |
| --- | --- | --- | --- | --- | --- | --- | --- | --- | --- | --- | --- |
|  | Total | Solo women | Solo men |  | Total | Solo women | Solo men |  | Total | Solo women | Solo men |
| Berlin | 52.5 | 8.2 | 26.8 |  | 34.2 | 3.7 | 15.0 |  | 8.6 | 1.5 | 3.6 |
| Hamburg | 64.8 | 10.3 | 33.4 |  | 39.1 | 4.5 | 17.4 |  | 8.7 | 1.7 | 3.4 |
| Cologne | 78.1 | 10.1 | 42.9 |  | 46.5 | 5.1 | 19.4 |  | 8.7 | 2.1 | 3.3 |
| Munich^a^ | 75.7 | 10.3 | 42.8 |  | 42.2 | 4.6 | 17.0 |  | 8.3 | 1.2 | 3.3 |
| Total | 63.3 | 9.3 | 33.7 |  | 38.5 | 4.2 | 16.5 |  | 8.5 | 1.5 | 3.5 |
| *Representative counties* | 37.4 | 6.7 | 17.0 |  | 22.1 | 2.5 | 8.7 |  | 4.5 | 1.0 | 1.7 |

a. “Munich” relates to the combined city and *Landkreis* (surrounding county).

## Table S14: Number of Inhabitants of the Big Cities and the Representative Counties

Number of inhabitants

| City | Absolute number of inhabitants |
| --- | --- |
| Berlin | 3,866,385 |
| Hamburg | 1,853,935 |
| Cologne | 1,073,096 |
| Munich^a^ | 1,868,381 |
| Total | 8,661,797 |
| *Representative counties* | 4,942,825 |

*Note*. Data source: Statistisches Bundesamt (2022b). See part G for the full reference.

a. “Munich” relates to the combined city and *Landkreis* (surrounding county).

# B. Assessment of the Veracity of the Data in Study 1

## 1. Robust Verification Process of the Data Source

- ***Exclusive Use of Verified Profiles*:** For this study, only profiles that successfully completed either the video verification process or the on-site verification were utilized. These methods form the core of Joyclub’s user authentication system, ensuring a high level of profile validity. The authenticity verification is a crucial component for confirming the identity and the consistency between virtual and real identities. Notably, the authenticity seal awarded after successful verification serves as a trust-building element within the community and offers protection against fake profiles (Joyclub, n.d.-d).
- ***Community Watchdog Function*:** In addition to formal verification processes, the community itself functions as an informal “watchdog,” as described on the Joyclub website. Users are encouraged to report suspicious profiles, adding a continuous, community-driven layer of verification to the platform’s formal processes. This community surveillance plays a critical role in maintaining the authenticity and reliability of profiles by continuously identifying and reporting potential fakes (Joyclub, n.d.-b).
- ***Video Verification Process*:** The video verification process involves a video call with a Joyclub representative, during which users present official identification documents. This process, known in Germany as “Videoverifikation,” serves a dual purpose: it confirms the user’s identity, ensuring the profile represents a real person, and it verifies the user’s age to comply with legal requirements for accessing adult content (“FSK18-Check”). The platform ensures that these verification processes comply with German legal requirements, particularly regarding age verification for adult content. It is notable that the platform explicitly prohibits profiles related to commercial sexual services, even though prostitution is legal in Germany, maintaining a focus on consensual, non-commercial interactions (Joyclub, n.d.-c).
- ***On-Site Verification Process*:** Joyclub’s on-site verification process, available at certain events, offers an additional option for users to verify their profiles. This in-person verification method complements the online video verification process. It is a rigorous procedure conducted by selected clubs, event organizers, and group leaders. Joyclub provides these verifiers with detailed instructions to ensure consistency and thoroughness in the verification process (Joyclub, n.d.-a).
- ***Key Aspects of the On-Site Process*:** Key aspects of the verification process, as outlined in Joyclub's instructions (Joyclub, n.d.-a), include:

1. ***Identity Verification Based on Government-Issued ID*:** Verifiers are required to check official identification documents (such as a national ID card, passport, or driver’s license) against the user’s profile information.
2. ***Age Verification*:** Special attention is paid to confirming the user’s age by carefully comparing the age stated in the official document with the age provided in the user’s profile. Verifiers are explicitly instructed to investigate any inconsistencies in age data.
3. ***Profile Type Confirmation*:** For couple profiles, both individuals must be present and verified simultaneously to ensure that the couple profile genuinely represents two people who voluntarily commit to maintaining a joint profile.
4. ***Comprehensive Check*:** Verifiers are required to confirm that the persons present match the profile description and images.
5. ***Discretion and Privacy*:** The process is designed to be discreet, typically conducted at the entrance of events, respecting users' privacy while maintaining rigorous standards.
6. ***Immediate Digital Recording*:** Successful verifications are immediately recorded in the Joyclub system, updating the user’s profile status in real-time.
7. ***Ongoing Training and Support*:** Joyclub provides ongoing guidance and support to verifiers, ensuring they remain up-to-date with the latest verification requirements and techniques.

- ***Conclusion*:** The structured and detailed on-site and video verification processes, based on government-issued identification documents, create a robust system for ensuring the authenticity of user profiles. This significantly reduces the likelihood of fake or misrepresented profiles being included in the analysis. Notably, the emphasis on verifying participants’ identity and age introduces a higher standard of authenticity than is typically found in social science research, where such verification is uncommon. This heightened verification rigor significantly enhances the reliability of the demographic data analyzed in this study, offering a stricter standard of data integrity compared to the field norm.

**2. Legal and Regulatory Context**

- ***Illegality of the Use of Fake Profiles Under German Law***: The operation of dating platforms in Germany is subject to strict legal regulations. Whereas the existence of a law does not guarantee compliance, the risk of detection of fake profiles by users, competitors, or consumer agencies is high.
- ***Even Terms and Conditions Allowing Fake Profiles are Illegal***: The Berlin Regional Court issued an injunction against a defendant online portal in January 2022, ruling that the use of fake profiles constitutes fraud and unfair competition, regardless of any terms and conditions that might suggest otherwise. A fine of up to EUR 250,000 or imprisonment for up to six months, to be enforced against the legal representatives of the defendant portal, was ordered for each individual case of violation of the injunction (Landgericht Berlin, 2022). Similarly, the Flensburg Regional Court ruled in October 2022 that any attempt by a platform to justify the use of fake profiles through their terms and conditions would not shield them from legal action. The court issued an injunction, accompanied by a similar penalty order, not only against the use of such profiles but also against the respective terms and conditions. The court emphasized that transparency and honesty are particularly crucial for platforms serving the dating market (Landgericht Flensburg, 2022). In this context, it is important to note that Joyclub does not employ such terms and conditions.
- ***Site Model; Risks of Detection and for the Reputation***: Sites that maintain fake profiles typically operate on a different revenue model that charges users per message or interaction (Verbraucherzentrale Bayern e.V., 2017), which is not the case with Joyclub. The significant risk of detection and reputational damage, particularly for a long-established platform like Joyclub, supports the credibility of their claim that the platform does not contain fake profiles. This has been clearly established in recent court rulings.
- ***Conclusion***: The stringent legal environment in Germany, combined with recent court rulings, underscores the importance of transparency and honesty in dating platforms. Joyclub’s operational model, which differs from those platforms that utilize fake profiles, along with the significant legal and reputational risks associated with the use of fake profiles, strongly supports the credibility of the profile data used in this study. The platform’s commitment to genuine user verification is further validated by the absence of terms and conditions that allow for fake profiles, ensuring a high standard of data authenticity.

**3. Visual Age Data Triangulation with data from Study 2**

- Visualizations of the age distributions suggest a high similarity between the age groups in Study 1 and Study 2. In Study 2, data were filtered to exclude events where participants had age restrictions and to remove records with incomplete demographic data, ensuring a more accurate comparison of age distributions.

**Figure S1: Age of Women Distribution Comparison**

*Comparison of age distribution among women in the two studies. The graphic illustrates the differences in age demographics between Study 1 and Study 2, highlighting the distribution.*


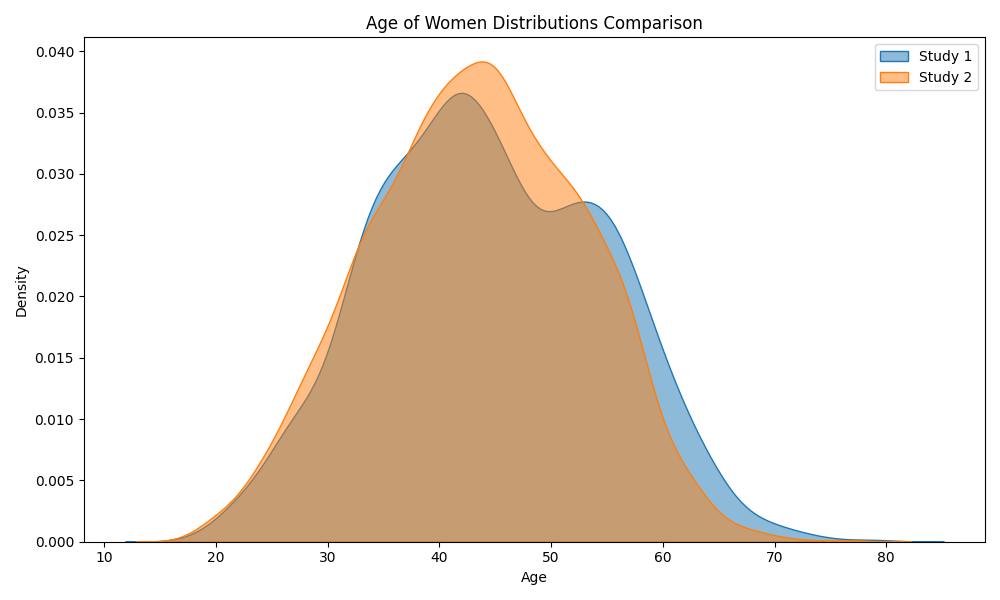


- Both distributions are unimodal and roughly bell-shaped.
- Study 2 (orange) has a slightly lower median age, with its peak shifted slightly to the left compared to Study 1 (blue).
- There is substantial overlap between the two distributions, indicating similar age patterns.
- Study 1 shows a slightly higher proportion of women in their 50s and early 60s.

**Figure S2: Age of Men Distribution Comparison**

*Comparison of age distribution among men in the two studies. The graphic illustrates the differences in age demographics between Study 1 and Study 2, highlighting the distribution.*


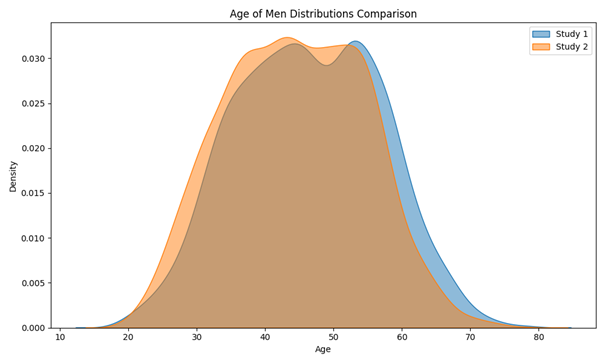


- Both distributions are unimodal and roughly bell-shaped.
- The overlap between Study 1 and Study 2 is even more pronounced for men than for women.
- Study 2 (orange) shows a slightly higher proportion of younger men (30s to early 40s) compared to Study 1.
- Study 1 (blue) shows a slightly higher proportion of men in their 50s and early 60s.

**4. Statistical Data Triangulation with data from Study 2**

A comparison of the age distributions and demographic patterns between Study 1 (profile-based) and Study 2 (event-based) yielded the following results:

- ***High Overlap Coefficients***: The overlap coefficients were 0.6234 for women and 0.7449 for men, indicating a high degree of consistency between these independently collected datasets. Although standard statistical tests indicated significant differences due to the large sample sizes, the practical significance of these differences is minimal, as evidenced by the high overlap coefficients and visual similarity of the distributions.
- ***Distribution Similarity Tests***: The low values for both Earth Mover's Distance (Graumann & Darrell, 2004) and Jensen-Shannon Divergence indicate a high degree of similarity between the age distributions in Study 1 and Study 2 for both genders:
  - Earth Mover's Distance—women: 0.0286
  - Earth Mover's Distance—men: 0.0290
  - Jensen-Shannon Divergence—women: 0.1142
  - Jensen-Shannon Divergence—men: 0.1113
- ***Note on the Large Sample Size Problem***: The relationship between sample size and statistical significance is well-established in statistical literature. As sample size increases, statistical tests become more sensitive to small differences. Therefore, large samples can lead to statistical significance even when practical differences are small (Kaplan et al., 2014; Lin et al., 2013; Sullivan & Feinn, 2012). As expected for this study, the tests typically applied for comparing samples (Kolmogorov-Smirnov test, Cramer-von Mises Test, Mann-Whitney U Test, and Two-Sample T-Test) indicated statistically significant differences between the age distributions in Study 1 and Study 2 for both genders. These statistical differences likely reflect minimal practical differences given the high overlap and distributional similarities observed.
- ***Explanation of Remaining Differences***: The observed differences may be attributed to genuine variations in the demographics of the overall population of identified swingers (Study 1) versus those who were admitted to events (Study 2), rather than data inconsistencies. For example, the slightly younger median age for admitted event attendees could reflect a real phenomenon in event participation within the swinger community.

The Python script used for the generation of Figures S1 and S2, as well as for performing the statistical comparisons, is contained in the data package accompanying the main study.

**5. General and Region-Based Correlation Analyses with Subgroups: Age and Gender**

- ***Correlation Of Study 1 with Subgroup Data***: Correlation analyses between the main dataset for Study 1 and a subset generated in August 2024, capturing the population of self-declared Open Relationship-practicing identified swingers maintaining a Joyclub profile (see Tables S8 and S9 for details), revealed significant positive correlations across multiple dimensions, particularly in age distributions and regional patterns, this including swinger density.
- ***Temporal Stability***: The consistency of data patterns across different time points of data harvesting (2023 for the main study and 2024 for the subgroup analyses) suggests stable demographic and behavioral patterns within the swinger community, further supporting the reliability of the data.

***In detail:***

- **Correlation of Swinger Density Values:** Regarding the representative dataset for Study 1, Table S11 highlights a correlation between the county-level values of Open Relationship swingers per 100,000 inhabitants, harvested in 2024, and the values of identified swingers per 100,000 inhabitants from the general dataset for Study 1, which is derived from the year 2023 (*r*_s_(22) = .46, *p* = .023[[1]](#footnote-2), 95% CI[−.04, .81]). Additionally, a correlation can be observed between the density values for Open Relationship swingers, also calculated from 2024 data, and the swinger density values from 2023 (*r*_s_(22) = .43[[2]](#footnote-3), *p* = .037, 95% CI[−.05, .78]). The density values of the two subsets show a strong correlation (*r*_s_(22) = .65, *p* < .001, 95% CI[.30, .86]), indicating consistent regional involvement patterns across different subsets studied at various points in time.
- **Correlation of Localized Age Data**: As shown in Table S9, the median ages of swingers, when assessed by county and by gender, in the representative counties set of Study 1 (harvested in 2023) strongly correlate with the corresponding data in the data set related to identified Open Relationship swingers (harvested in 2024): the correlation for the median age for women amounts to *r*_s_(22) = .68 (*p* < .001, 95% CI[.48, .80]) and that regarding men to *r*_s_(22) = .76 (*p* < .001, 95% CI[.60, .84)]. Similar correlations of the representative counties set of Study 1 with the corresponding data in the data set related to identified Open Relationship swingers (harvested in 2024) are shown in Table S10: With respect to the median age women in the single counties, the correlation amounts to *r*_s_(22) = .56 (*p* = .006, 95% CI[.15, .82]); with regard to men, the value is *r*_s_(22) = .44[[3]](#footnote-4) (*p* = .037, CI[−.01, .74]). This shows that regional patterns in the data persist across years and subsets.
- **Age Distribution:**
  - *Study 1 (2023):*

Median age for women: 44 years, for men: 46 years

- - *2024 Subgroups of Swingers (Median ages):*

Open Relationship: Women: 40 years, Men: 42 years.

Open Relationships: Women: 43 years, Men: 45 years.

Poly: Women: 41 years, Men: 42 years.

For the subsample size, standard deviations and confidence interval data, please refer to Table S8.

- **Age Differences in Couples:**
  - *Study 1 (2023)*:

Median age difference of 3 years (men older)

- - *2024 Subgroups*:

Open Relationship: Median age difference of 3 years.

Open Relationships: Median age difference of 3 years.

Poly: Median age difference of 2 years.

The consistency in age differences between partners across different years and subgroups is remarkable. This replication of the 3-year median age difference (with men being older) provides strong support for the reliability of the findings regarding couple dynamics in the swinger community.For the subsample size, standard deviations and confidence interval data, please refer to Table S8.

- **Statistical Consistency—Standard Deviation and Kurtosis**: The similarity in statistical measures like standard deviation and kurtosis across the 2023 and 2024 datasets for age distributions (see Table S8) strengthens the validity of the data. Notably, the Open Relationship and Open Relationship swinger subgroups exhibit stable age distribution patterns, with consistently observed negative kurtosis values indicating a flatter distribution than normal, maintained across both years. The data quality for these subgroups appears robust, with no missing data and narrow confidence intervals in bootstrapped estimates, supporting the reliability of the findings. Low bias and standard error further support the data’s validity.
  In contrast, the Polyamorous swingers subgroup, with a smaller sample size (*n* = 46), shows some variability. While skewness and kurtosis for women’s ages align with other subgroups, the positive skewness in men’s ages and age differences, along with slightly positive kurtosis, suggest that the variability may stem from the smaller sample size. The wider confidence intervals in this subgroup's measures indicate lower precision, suggesting that while the observed patterns are informative, they should be interpreted with caution.
- **Gender Ratio**: The predominance of solo men profiles observed in the 2023 data is consistently replicated across all 2024 subgroups (Open Relationship: 1180 vs 671, Open Relationships: 698 vs 392, Poly: 129 vs 94). This persistent pattern across different years and swinger subcultures strengthens the validity of the gender distribution findings.
- **Socioeconomic Correlations**: The positive correlation between men’s median age and average county income observed in 2023 (*r*_s_(22) = .46, *p* = .025, 95% CI[.07, .75]), with a relevant effect size, is not only replicated but strengthened in the 2024 data for swingers who state a Open Relationship inclination on their profile (*r*_s_(22) = .48, p = .018, 95% CI[.18, .71]). This reinforces the findings on the relationship between swinger demographics and socioeconomic factors. NB: This correlation could not be found in the data for the Open Relationship subgroup.
- **Conclusion—Demographic Diversity and Consistent Patterns**: Both the 2023 and 2024 data challenge stereotypes about swingers, showing a diverse community that spans various age groups, relationship styles, and socioeconomic backgrounds. The consistent patterns across different subgroups, notably the Open Relationship subgroup, further support the robustness of these findings. The data demonstrate a consistent pattern: Counties with higher median incomes show a higher proportion of inhabitants with a migration background. This correlates with a higher median age of swinger men and a larger age difference in swinger couples, although the median age of swinger women remains unaffected by county affluence. Conversely, lower income and associated socioeconomic parameters correlate to a lower median age of swinger men. This consistency is evident in swinger data collected at different times, with nearly a year between data collection points, and across various subgroups. The replication of key patterns—such as swinger density, age distributions, gender ratios, couple age differences, and socioeconomic correlations—in most of the independently collected datasets significantly reduces the likelihood that these patterns are artifacts of data collection methods or sample biases. Moreover, the slight variations observed between the main 2023 dataset and the 2024 subgroups (e.g., slightly younger median ages in some subgroups) align with expected real-world data, reflecting natural variations within the swinger community.Other inconsistencies may be owed to the size of the respective data subsets; the largest subset, being the Open Relationship swinger subset, confirms all core structural findings of Study 1. The resulting combination of stable core patterns and nuanced variations across years and subgroups strongly supports the veracity of the data. It suggests that the methodology applied captures genuine demographic characteristics of the swinger community rather than artificial or manipulated data patterns.

**6. Analysis of Potential Profile Duplication – Couples and Solos with Duplicate Profiles of the Same Type**

- ***Introduction***: An analysis was performed to investigate the prevalence of duplicate profiles within the Study 1 data by comparing the user profile data in the representative county set with the independently harvested event admission records from Study 2. The primary objective was to estimate the likelihood and extent of profile duplication across different user categories: solo women, solo men, and couples.
- ***Application of a Python Script***: The analysis employs a custom Python script (contained in the data package to the study), designed to process the two distinct corpus datasets: one containing the user profile information for Study 1, and another comprising event admission data for Study 2. The following detailed explanation outlines what the script calculated, the reasoning behind the calculations, the methodology employed, and the interpretation of the results, and their impact on the results.
- ***Data Sources***: The script begins by loading two datasets:
  - ***Study 1*** (Residents Data): This dataset includes 6,173 user profiles, categorized into 917 solo women, 3,038 solo men, and 2,218 couples. It serves as the primary source of profile information.
  - ***Study 2*** (Admissions Data): This dataset contains event aadmission records, which are considered more reliable due to the improbability of individuals attending multiple events simultaneously.
- ***Script Overview***: The script’s primary purpose is to calculate the expected duplicates of profiles based on the observed data in Study 2, with additional adjustments related to the different distributions of profile types in the two datasets, and then apply these adjustments to the profiles in Study 1. This adjustment process is crucial. Without considering the inherent community-related structure in profile characteristics—such as age—a direct comparison of overlaps in Study 1 could lead to erroneous conclusions about the prevalence of duplicate profiles. To avoid self-referencing, the adjustments were solely conducted on the basis of the independently harvested Study 2 data.
- ***Methods****:* This analysis used a custom Python script to process two datasets. The first dataset (Study 1) contained user profiles (n = 6,173), categorized into 917 solo women, 3,038 solo men, and 2,218 couples. The second dataset (Study 2) contained event admission data, which was considered more reliable due to the improbability of individuals attending multiple events simultaneously. The objective of the script was to predict the expected number of duplicate profiles in Study 1, based on the profile distributions in Study 2.
  - The process involved several computational steps. First, profiles in Study 1 were divided by type (solo women, solo men, and couples). The profiles from Study 2 were then randomly sampled to match the size and structure of the Study 1 profiles for each county. This random sampling preserved the number of profiles but replaced the profile characteristics (age and gender) based on Study 2 data, ensuring that no self-referencing occurred.
  - The "use-once" rule was applied within each county, allowing profiles from Study 2 to be reused across different counties but never within the same county and iteration. This adjustment ensures localized comparison and avoids artificially inflating the number of duplicates. Given the 10,000 iterations of the sampling process, nearly all profiles from Study 2 are highly likely to be used at least once, ensuring that the simulation fully leverages the available data.
  - The next step involved detecting duplicates in both the observed Study 1 data and the simulated dataset (where profiles from Study 2 filled the structure of Study 1). For solo women and solo men, only their respective ages were used in the matching process. For couples, both ages were matched simultaneously. Duplicates were defined as any profiles of the same type within the same county that shared identical age characteristics, with multiple occurrences of the same profile counted as duplications.
  - Finally, the differences between the observed and simulated duplicates were computed. The script used bootstrap resampling (10,000 iterations) to calculate the confidence intervals for the estimates of real duplicates. The real duplicates were calculated as the number of observed duplicates exceeding the number expected based on Study 2 data.
- ***Results*:**
  - For **solo women**, the estimated real duplicates were approximately **9.6 (95%CI[4.1, 16.1])**, affecting about **1.1%** of the profiles. The Wilcoxon signed-rank test did not indicate a statistically significant difference between observed and simulated duplicates (*p* = **.160**).
  - For **solo men**, the estimated real duplicates were approximately **33.2 (95%CI[20.1, 47.3])**, affecting about **1.1%** of the profiles. The Wilcoxon signed-rank test showed a statistically significant difference between observed and simulated duplicates (*p* = **.027**).
  - For **couples**, the estimated real duplicates were approximately **19.2 (95%CI[4.4, 40.3])**, affecting about **0.9%** of the profiles. The Wilcoxon signed-rank test did not indicate a statistically significant difference (*p* = **.060**).
- ***Test of the Statistical Model***: For testing its accuracy and the overall quality of the data, the model was applied to a dataset with data from the four big cities in Germany added to the representative dataset. The model predictions for overlaps (i.e., number of profiles of the same type with the same age or ages in the same city), which were solely informed by Study 2 data and the structure (numbers of profiles per type and county) of Study 1, closely matched the actual number of overlaps observed:
  - *Berlin: Solo Women*: The model predicted **45.34** simulated duplicates, with **51 observed duplicates**, resulting in an observed discrepancy of **5.66 overlaps**. *Solo Men*: The model predicted **48.85** simulated duplicates, with **47 observed overlaps**, showing **near-perfect alignment**. *Couples*: The model predicted **256.81** simulated overlaps, with **256 observed overlaps**, representing **exceptional accuracy** for couples in Berlin.
  - *Munich: Solo Women*: Predicted duplicates were **43.49**, with **43 observed overlaps**, showing **perfect prediction**. *Solo Men*: The model predicted **48.58**, with **46 observed overlaps**, showing a **slight discrepancy**, but the model still performs well overall. *Couples*: The model predicted **259.65** overlaps, with **257 observed overlaps**, showing **strong alignment**.
  - *Hamburg: Solo Women*: Predicted duplicates were **44.08**, with **44 observed overlaps**, showing **perfect alignment**. ***Solo Men***: Predicted overlaps were **48.59**, with **50 observed overlaps**, showing a **minor discrepancy**. *Couples*: Predicted overlaps were **216.63**, with **229 observed overlaps**, showing a slightly larger discrepancy for couples.
  - *Cologne: Solo Women:* Predicted overlaps were **41.91**, with **41 observed overlaps**, showing **perfect alignment**. *Solo Men:* The model predicted **48.87**, with **51 observed overlaps**, showing a **small discrepancy**. *Couples:* Predicted overlaps were **199.16**, with **200 observed overlaps**, showing **near-perfect alignment**.
- ***Conclusion***: The results indicate a low prevalence of duplicate profiles in Study 1, with overlap rates just over 1% for solo women and men, and under 1% for couples. Real duplications cannot be entirely dismissed, particularly for solo men where statistical significance was found, but they appear limited across all categories. The observed discrepancies between predicted and actual overlaps likely result from random variation inherent in the sampling process and structural differences between the two datasets. The almost perfect predictions that the model, informed by Study 2 data, made for the Study 1 data regarding the big cities further reinforce the reliability of the data and the model itself. The data from Study 1 can be considered reliable for drawing conclusions about the population under study, with minimal impact from duplicate profiles.

**7. Analysis of Potential Profile Duplication – Solo Profile Owners in Couple Profiles**

- ***Theoretical Framework*:** The potential for multiple profiles in online swinger communities aligns with established theories of online identity management (Bullingham & Vasconcelos, 2013) and the concept of context-dependent identity exhibition (Hogan, 2010). In the swinger community, maintaining separate solo and couple profiles could be a strategic choice to navigate different social scenarios or relationship configurations. This approach is supported by Zhao et al.’s (2008) research on nonymous online environments, which posits that online identities are integral parts of individuals’ overall identity production, particularly relevant in contexts where online interactions are inherently connected to offline encounters.
- ***Methods*:** To quantify the maximum potential for duplicate profiles within the online swinger community, a Python script was developed to analyze age and location data across all counties. The script focuses on detecting duplicates between solo and couple profiles that are localized within specific counties. Given the county-based localization of the profiles, the script applies both demographic and geographical adjustments to enhance the accuracy of its predictions. Below is a detailed description of the script and its key functions:

1. **Data Preparation:**
   - The script begins by loading datasets from Study 1, which contain profiles from various counties. These profiles are categorized by gender and profile type (solo or couple). The profiles are bound to specific counties, meaning the detection of matches (potential indicators of duplicate profiles) is also restricted within these county boundaries.
   - Profiles are then aggregated based on matching ages, which serves as the primary criterion for detecting potential matches.
2. **Matching Process:**
   - A *match* is defined as an occurrence where a solo profile matches the age of a person in a couple profile with the same gender and is located in the same county. Once a solo profile is used for detecting a match, it cannot match another couple profile again. Similarly, a couple profile that has been used to detect a match with a solo profile cannot match another solo profile of the same gender, although it can still match a solo profile of the opposite gender.
   - Importantly, *matches* are not the same as *overlaps* or *duplicates*. Matches serve as a preliminary step in identifying where potential overlaps (multiple profiles by the same person) might occur. *Overlaps* are matches which serve as a basis for futher adjustments and calculations, whereas *duplicates* are the results of the calculations, likely representing real cases where persons maintain a solo profile and participate in a couple profile.
3. **Demographic and Geographical Adjustments:**
   - *Random Profile Generation and Matching:*
     - Initially, the script generates a total random distribution of profiles across the structure of Study 1 counties, without using Study 2 data. The generated profiles mimic the structure of Study 1 counties in terms of size and gender distribution. This step involves assessing matches over 1,000 iterations, which provides a baseline “natural” distribution of matches that might occur purely by chance.
   - *Demographic Adjustment Using Study 2 Data:*
     - In a subsequent step, the Study 1 counties are filled with profiles generated using data from Study 2. This process also involves 1,000 iterations, and matches are assessed again. The difference between these matches and those from the initial random distribution is calculated across counties, genders, and ages. These differences inform the demographic adjustment factors.
     - To simplify the data and reduce dimensions, these factors are then averaged, weighted by the share of each county in the total population size, and reduced to the dimensions of gender and age. As the Study 2 data are not localized with a view to the place of residence, this approach is systematically sound. This adjustment ensures that the matches better reflect the demographic realities of the swinger community as informed by Study 2 data.
   - *Geographical Adjustment:*
     - Beyond the demographic adjustments, the script applies geographical adjustments based on the distribution of age strata within the general population of each county, sourced from official demographic statistics. These geographical adjustments occur in the county and age dimensions but not in the gender dimension, ensuring that the overlap predictions reflect the actual population *age distribution within each county.*
   - *Prediction and Comparison:*
     - The script uses the combined demographic and geographical adjustments to predict the number of overlaps (potential multiple profiles) and compares these predictions to the actual observed overlaps in Study 1 data. The comparisons identify potential multiple profiles by evaluating where the observed overlaps exceed those predicted by chance.
     - The results are then refined and reported in a manner that highlights any significant discrepancies between predicted and observed overlaps, indicating areas where multiple profiles are more likely.
   - *Calculation of Results and Confidence Interval Values*: To assess the duplication estimates and their reliability, a bootstrap procedure with 10,000 iterations was applied. By bootstrapping the final duplication results (after all adjustments were applied), the complexity of combining confidence intervals from the multiple calculation steps is avoided. Bootstrapping estimates the overall variability in the final result, capturing the uncertainty introduced by earlier steps in a holistic way, including potential error cancellation and compensatory effects. As the bootstrap method resamples the final data to generate a distribution of possible outcomes, it provides a robust way to directly calculate the 95% confidence intervals, without needing to aggregate confidence intervals from intermediate stages. The Python script for this calculation is included in the dataset accompanying the study.

- ***Results*:** The analysis revealed an estimated number of 1,734 individuals (95% CI[1,561, 1,908]) in counties including big cities, potentially affecting 610 solo women (CI[526, 700)] and 1,124 solo men profiles (CI[994, 1,261]), who also participate in a couple profile. This represents 16.0% (95% CI[14.4, 17.6]) of the total 10,819 solo profiles in the dataset including the big cities. When excluding big cities, the estimated duplication drops to 748 individuals (95% CI[673, 820]), affecting 248 solo women (CI[215, 283]) and 501 solo men profiles (CI[435, 571]), representing 18.9% (CI[17.0, 20.7]) of the 3,955 solo profiles in the dataset without big cities.

  The similar duplication percentages in both datasets (16.0% with big cities, 18.9% without big cities) suggest that the script is robust in its ability to detect potential duplicates, regardless of the inclusion of large urban areas. This consistency in results, even when the dataset is restricted to counties without big cities, strengthens the validity of the findings and indicates that the method is not overly sensitive to the presence of large urban centers. This robustness underscores the reliability of the script in different geographic contexts.
- ***Correlation Analysis***: A comprehensive correlation analysis was conducted to examine the relationships between duplication values and various demographic factors. The analysis was performed on two datasets: one including big cities and another excluding them. Spearman’s rank correlation coefficient was used due to its robustness against non-normal distributions, and bootstrap confidence intervals were calculated to assess the reliability of the correlations.

1. **Dataset Including Big Cities (Berlin, Hamburg, Cologne, Munich)**
   - *Population Size:* A strong positive correlation was observed (*r*_s_(27) = .92,
     *p* < .001, 95% CI[.83, .97]). This indicates a very robust relationship between population size and duplicate values, with larger populations strongly associated with more duplicates.
   - *Population Density:* A moderate positive correlation was found (*r*_s_(27) = .50, *p* = .007, 95% CI[.12, .77]). This suggests that more densely populated areas tend to have more duplicates, though the relationship is not as strong as with total population size.
   - *Swinger Density:* A moderate positive correlation was observed (*r*_s_(27) = .51, *p* = .006, 95% CI[.15, .77]). This indicates that areas with a higher concentration of swingers tend to have more profile duplicates.
   - *Median Age:* No significant correlation was found for men (*p* = .657) and for women (*p* = .614).
2. **Dataset Excluding Big Cities (Representative Counties)**
   - *Population Size*: A moderate positive correlation was found (*r*_s_(22) = .43,
     *p* = .023, 95% CI[.04, .71]). While still significant, this correlation is notably weaker than in the dataset including big cities.
   - *Population Density*: A stronger positive correlation was observed
     (*r*_s_(22) = .62, *p* < .001, 95% CI[.28, .83]). This suggests that population density becomes a more important factor when big cities are excluded.
   - *Swinger Density*: A moderate to strong positive correlation was found (*r*_s_(22) = .59, *p* = .001, 95% CI[.25, .81]). This relationship remains robust even when big cities are excluded.
   - *Median Age*: No significant correlation was observed for men (*p* = .305) and for women(*p* = .677).

- ***Repeated Analysis of Correlations With Demographic Data***: In the representative dataset, the estimated duplicate data had been used to adjust the calculation of persons involved in Joyclub profiles in the county dimension by substraction of the duplicates, and to calculate an adjusted “swinger density” value (“identified swingers” per 100,000 inhabitants). Spearman analysis of correlations between the adjusted “swinger density” with the sociodemographic data had been repeated on this basis. No new correlations were detected, and the existing correlation with the population densities of the counties resulted in values similar to the values already reported for the unadjusted swinger density: *r*_s_(22) = .50, *p* = .014, 95% CI[.15, .72]) for the adjusted swinger density value versus *r*_s_(22) = .46, *p* = .023, 95% CI[.09, .72]) for the unadjusted swinger density value. The results for the adjusted values are situated within the confidence interval of the original reported values.
- ***Interpretation and Implications*:** The potential for multiple profiles should not be conflated with deceptive practices. Hancock et al.’s (2007) study on deception in online dating profiles found that users generally do not lie about basic demographic information, particularly age. This finding is especially relevant in the context of Joyclub, where accurate representation of age and location is crucial for facilitating real-world swinger interactions. The practical necessity of truthfulness in these areas suggests that users may maintain multiple profiles, but the information within these profiles is likely to be largely accurate.

  This theoretical assumption is supported in the case of Joyclub by the findings discussed earlier, particularly regarding profile verification, the community serving as a “watchdog,” and the high consistency of datasets harvested at different times using different filter criteria. The study focuses on how individuals participate in the swinger community, rather than their relationship status, acknowledging that participants may engage with the community in multiple capacities. This approach mirrors offline behavioral patterns observed in Study 2, where it was found that 8.83% of admitted guests at events were “accompanied solos”—individuals who registered for events as part of a couple using a solo profile.

  The findings suggest that population size and density are the primary drivers of profile duplicates within the online swinger community, particularly in urban areas. The significant correlations with these factors highlight that larger and more densely populated counties are more likely to have duplicate profiles. Nevertheless, when the swinger density data are adjusted by the potential duplicate values, the resulting correlations to demographic data only slightly changed, and the overall findings reported here remain unaffected. The duplication issue did not have any sizeable impact on the reported results.

A decrease in duplicate percentages for both genders when including big cities could be observed. This might indicate that users in larger urban areas are less likely to maintain both solo and couple profiles, possibly due to a larger and more diverse dating pool. The more pronounced decrease in women's duplicate percentage (from 27.0% to 18.8%) compared to men’s (from 16.5% to 14.8%) suggests that women in larger cities are particularly less likely to have duplicate profiles. In both analyses, women show a higher tendency for duplicate profiles compared to men, but this gender difference is less pronounced when big cities are included.

The overall higher duplicate percentage among solo women in non-urban areas suggests that these women may be more likely to maintain multiple profiles in these settings. This might reflect a closer-knit community where individuals are more likely to engage in different social scenarios, leading to a higher duplicate detection rate. The discrepancy between urban and non-urban areas could also indicate that the diversity and anonymity of big cities reduce the need or opportunity for maintaining multiple profiles, leading to fewer duplicates being detected.

- ***Remarks on the Statistical Methodology:***

1. **Robustness of Findings**: The use of Spearman’s rank correlation and bootstrap confidence intervals enhances the statistical soundness of the analysis. These non-parametric methods are resistant to outliers and do not assume normal distribution of the data.
2. **Consistency Across Datasets**: The analysis of both datasets (with and without big cities) provides insight into the robustness of the relationships. Population-related factors (size, density) and swinger density consistently show significant positive correlations with duplicate values, indicating these relationships are not merely artifacts of including large urban areas.
3. **Effect of Urban Areas**: The dramatic strengthening of the population size correlation when including big cities (from *r*_s_(22) = .43 to *r*_s_(27) = .92) suggests that large urban areas have a substantial impact on profile duplicates. This finding is statistically sound and practically significant.
4. **Age Relationships**: The lack of significant correlations with median age in both datasets suggests that age is not a strong predictor of profile duplicates. The consistency of this finding across datasets adds to its reliability.
5. **Precision of Estimates**: The bootstrap confidence intervals provide a measure of the precision of our correlation estimates. The narrow intervals for population size and swinger density correlations indicate more precise estimates.
6. **Demographic Adjustment**: The use of official sources for demographic data enhances the reliability of the geographical adjustments applied in the analysis. This approach ensures that the duplicate predictions accurately reflect the population age distribution within each county, strengthening the overall validity of the results.
7. **Multiple Comparisons**: Whereas multiple correlations were conducted, the primary analysis does not rely heavily on these comparisons. The 1,000 iterations in the Monte Carlo simulation effectively minimize random effects, reducing the risk of Type I errors that could arise from multiple comparisons. This approach enhances the robustness of the findings without necessitating formal corrections for multiple comparisons.
8. **No Overfitting:** The similarity between the datasets used in Study 1 and Study 2, as demonstrated above, mitigates concerns about overfitting. The consistency of results across datasets with and without big cities (16% and 17% duplicates, respectively) further supports the generalizability of the findings, indicating that the model is not overly tailored to specific data characteristics.
9. **Outlier Handling**: The approach of handling outliers through demographic adjustment rates addresses the problem in this study. By incorporating county-level demographic data, the method inherently accounts for regional variations that might otherwise appear as outliers. This approach maintains the integrity of the data while ensuring that extreme values do not disproportionately influence the results.
10. **Confidence Intervals:** The bootstrap method employed in the correlation analysis provides robust confidence intervals for the duplicate estimates. These intervals offer insight into the precision and reliability of the findings. For instance, the narrow confidence interval for the correlation with population size (95% CI[.83, .97] in the dataset including big cities) indicates a high degree of certainty in this relationship.
11. **No Normality Assumption**: The use of Spearman’s rank correlations appropriately addresses potential concerns about non-normal distributions in the data. This non-parametric approach ensures the validity of the correlation analyses without requiring the assumption of normality, which is particularly important given the diverse nature of the demographic and behavioral data being analyzed.

- ***Limitations and Future Research***: It is important to acknowledge that this study represents a snapshot of the online swinger community at a specific point in time. Whereas this limitation is inherent to the cross-sectional nature of the data, it opens avenues for future research. Longitudinal studies could provide valuable insights into how profile duplicates patterns and community dynamics evolve over time. The moderate correlations with population density and swinger density, while statistically significant, indicate more complex relationships that may require further investigation. Future research could refine this approach by incorporating additional data points or employing more sophisticated matching algorithms. Additionally, qualitative research exploring users’ motivations for maintaining multiple profiles could provide valuable insights into this phenomenon.
- ***Conclusion***: The very strong correlation with population size (when including big cities) suggests that this value forms a key factor in understanding profile use within the community. In any setting, the overwhelming majority of solo profile users does not participate in a couple profile. The analysis of potential profile duplication in the Joyclub swinger community reveals a potential interplay between online identity management, practical considerations of facilitating real-world interactions, and the multifaceted nature of participation in the swinger lifestyle. The evidence on the lack of widespread use of several profiles of the same type by the same persons (e.g. solo profile owners maintaining a second solo profile, see sub 7 below) suggests that, as expected, participation of the same persons in profiles of two types, couple and solo, likely reflects the nuanced ways individuals navigate their involvement in the community rather than attempts at deception. This contributes to the understanding of online behavior in nonymous, sexually-oriented platforms and highlights the importance of considering both online and offline contexts in digital identity research.

**8. Methodological Considerations**

- ***General Limitation***: Whereas the inherent limitations of online data collection must be acknowledged, the approach pursued in this study aligns with established methodologies in social science research.
- ***The Use of Self-Reported Data is an Accepted Practice***: The use of self-reported data from verified online profiles is a widely accepted practice, particularly when studying populations that may be difficult to access through traditional sampling methods, in particular adherents to CNM practices (Balzarini et al., 2019; Haupert et al., 2017; Moors et al., 2017).
- ***Potentially Higher Authenticity Than in Anonymous Surveys***: Self-declaring as a swinger on a platform like Joyclub is in many ways similar to an anonymous survey. The main difference—and potentially even advantage—of my method is that users have not created their profiles specifically for a study, but for their actual participation in the swingers community. This could effectively result in more authentic data than a targeted survey.

**References Related to Part B**

Balzarini, R. N., Dharma, C., Kohut, T., Holmes, B., Campbell, L., Lehmiller, J. J., & Harman, J. J. (2019). Demographic comparison of American individuals in polyamorous and monogamous relationships*. Journal of Sex Research, 56*(6), 681–694. <https://doi.org/10.1080/00224499.2018.1474333>

Bullingham, L., & Vasconcelos, A. C. (2013). ‘The presentation of self in the online world’: Goffman and the study of online identities*. Journal of Information Science, 39*(1), 101–112. https://doi.org/10.1177/0165551512470051

Grauman, K., & Darrell, T. (2004). Fast contour matching using approximate Earth mover’s distance. In *Proceedings of the 2004 IEEE Computer Society conference on computer vision and pattern recognition, 2004* (Vol. 1). https://doi.org/10.1109/cvpr.2004.1315035

Hancock, J. T., Toma, C., & Ellison, N. (2007). The truth about lying in online dating profiles. In B. Begole, S. Payne, E. Churchill, R. St. Amant, D. Gilmore, & M. B. Rosson (Eds.), *CHI07: CHI conference on human factors in computing systems, San Jose, California, USA, April 28–May 3, 2007* (Vol. 1, pp. 449–452). Association for Computing Machinery. https://doi.org/10.1145/1240624.1240697

Haupert, M. L., Gesselman, A. N., Moors, A. C., Fisher, H. E., & Garcia, J. R. (2017). Prevalence of experiences with consensual nonmonogamous relationships: findings from two national samples of single Americans. *Journal of Sex & Marital Therapy*, *43*(5), 424–440. https://doi.org/10.1080/0092623X.2016.1178675

Hogan, B. (2010). The Presentation of self in the age of social media: Distinguishing performances and exhibitions online. *Bulletin of Science Technology & Society, 30*(6), 377–386. https://doi.org/10.1177/0270467610385893

Joyclub. (n.d.-a). *Echtheitsprüfung - Informationen für Clubs und Gruppenleiter* [Authentication - Information for clubs and group leaders]. Retrieved August 11, 2024, from https://support.joyclub.com/hc/de/articles/4411481513874-Echtheitsprüfung-Informationen-für-Clubs-und-Gruppenleiter

Joyclub. (n.d.-b). *Einzelprofil / Paarprofil ( Unterschiede, Umstellung )* [Single profile / couple profile ( differences, conversion )]. Retrieved August 11, 2024, from https://support.joyclub.com/hc/de/articles/10334747435794-Einzelprofil-Paarprofil-Unterschiede-Umstellung

Joyclub. (n.d.-c). *Möglichkeiten der Echtheitsprüfung und Altersverifizierung* [Authentication and age verification options]. Retrieved August 11, 2024, from https://support.joyclub.com/hc/de/articles/360012593880-Möglichkeiten-der-Echtheitsprüfung-und-Altersverifizierung

Joyclub (n.d.-d). *Registrierung und Profilarten / Profiltyp ändern* [Registration and profile types / Change profile type]. Retrieved August 11, 2024, from https://support.joyclub.com/hc/de/articles/360020584820-Registrierung-und-Profilarten-Profiltyp-ändern

Kaplan, R. M., Chambers, D. A., & Glasgow, R. E. (2014). Big Data and Large Sample Size: A cautionary note on the potential for bias. *Clinical and Translational Science, 7*(4), 342–346. https://doi.org/10.1111/cts.12178

Landgericht Berlin. (2022). Judgment of January 27, 2022, 16 O 62/21. https://www.vzbv.de/sites/default/files/2022-03/LG%20Berlin_27.01.2022.pdf

Landgericht Flensburg. (2022). Judgment of October 28, 2022, 8 O 29/22. https://www.vzbv.de/sites/default/files/2023-01/LG%20Flensburg_%2028.10.2022.pdf

Lin, M., Lucas, H. C., & Shmueli, G. (2013). Too big to fail: Large samples and the p-value problem. *Information Systems Research, 24*(4), 906–917. https://doi.org/10.1287/isre.2013.0480

Moors, A. C., Matsick, J. L., & Schechinger, H. A. (2017). Unique and shared relationship benefits of Consensually Non-Monogamous and monogamous relationships. *European Psychologist, 22*(1), 55–71. https://doi.org/10.1027/1016-9040/a000278

Sullivan, G. M., & Feinn, R. (2012). Using effect size—or why the p value is not enough*. Journal of Graduate Medical Education, 4*(3), 279–282. https://doi.org/10.4300%2FJGME-D-12-00156.1

Verbraucherzentrale Bayern e.V. (2017, January 15). *Online-Dating: Fake-Profile auf 187 Portalen* [Online dating: fake profiles on 187 portals]. Verbraucherzentrale Bayern. Retrieved August 18, 2024, from https://www.verbraucherzentrale-bayern.de/pressemeldungen/digitale-welt/onlinedating-fakeprofile-auf-187-portalen-22016

Zhao, S., Grasmuck, S., & Martin, J. (2008). Identity construction on Facebook: Digital empowerment in anchored relationships. *Computers in Human Behavior, 24*(5), 1816–1836. https://doi.org/10.1016/j.chb.2008.02.012

# C. Estimate of the Number of „Identified Swingers“ in Germany

## 1. Analysis of Joyclub Profiles

The study allows for an estimate of how many "identified swingers," according to the above definition, might live in Germany. This is a breakdown of the statistical analysis:

### Joyclub Profiles of “Identified Swingers”

- - *In the total dataset, including big cities*: The study recorded 22,973 persons in Joyclub profiles (9,325 women, 13,648 men) meeting the criteria of “identified swingers.” These are individuals who maintain a verified Joyclub profile, explicitly declare themselves as swingers, and provide a profile picture. This total includes both solo profiles and individuals who are part of couple profiles.
  - *In the representative dataset (excluding big cities)*: 8,391 “identified swingers” (3,135 women and 5,256 men) meeting the criteria were recorded.

## 2. Estimated Duplicates in Solo and Couple Profiles

A *duplicate* in this context refers to individuals who maintain both a solo profile and are part of a couple profile. This duplication needs to be accounted for to avoid overestimation. The method for calculating this overlap is detailed in part B.7 of this Supplementary Material. The estimates are:

- - - In counties including big cities: 1,734 individuals (95% CI[1,561, 1,908]) in counties including big cities, potentially affecting 610 solo women (CI[526, 700)] and 1,124 solo men profiles (CI[994, 1,261]), and
    - in the representative dataset (excluding big cities): 749 individuals (95% CI[673, 820]), affecting 248 solo women (CI[215, 283]) and 501 solo men profiles (CI[435, 571]).

### Adjusted Numbers of “Identified Swingers”

After deducting the estimated duplicates, the adjusted numbers of unique “identified swinger” profiles are:

- - *Total dataset*: 21,239 individuals (95% CI[21,065, 21,412)] with 8,715 women (CI[8,625, 8,799)] and 12,524 (CI[12,387, 12,654]) men.
  - *Representative dataset*: 7,642 individuals (95% CI[7,571, 7,718]) with 2,887 women (CI[2,852, 2,920]) and 4,755 men (CI[4,685, 4,821]).

These figures represent the estimated number of unique individuals with “identified swinger” profiles, accounting for potential duplications between solo and couple profiles.

## 3. Estimating the Prevalence of “Identified Swingers”

### Population Coverage of the Datasets

- - *Total dataset*: Covers counties with a population of 13,604,622 individuals, representing 16.344441% of the German population.
  - *Representative dataset*: Covers counties with a population of 4,942,825 individuals, representing 5.938246% of the German population.
  - *Total German population (for reference)*: 83,237,124.

These figures contextualize the sample sizes in relation to the entire German population.

### Extrapolation to Total German Population (ages 18–75)

Based on the proportions found in the datasets, the further calculation extrapolates the number of "identified swingers" to the entire German population aged 18–75:

- - *Based on total dataset*: 129,793 individuals (95% CI[128,729, 130,852]) with 53,278 women (CI[52,727, 53,791]) and 76,515 men (CI[75,677, 77,311]).
  - *Based on representative dataset*: 128,557 individuals (95%CI[127,361, 129,836)] with 48,567 women (CI[47,960, 49,105]) and 79,990 men (95%CI[78,811, 81,101]).

These extrapolations exclude individuals above 75 years old (18 men and 7 women in the total dataset; 5 men and 3 women in the representative dataset), that had been deducted from the results and CI values before extrapolation.

### Percentage of “Identified Swingers” in German Population (ages 18–74):

- - *German population aged 18-74: total*: 60,847,730 persons with 30,399,828 women and 30,447,902 men.
  - The extrapolated figures suggest that 0.21% of the German population aged 18-74 would meet the criteria of "identified swingers" according to both datasets (the upper value in the confidence interval of the extrapolation that is based on the total dataset would lead to a value of 0.22%); for women, the value amounts to 0.16% based on the values of the representative dataset and to 0.18% based on the total dataset, and for men to 0.25% based on the values of the representative dataset, and to 0.26% based on the values of the total dataset (the upper value in the confidence interval of the extrapolation that is based on the total dataset would lead to a value of 0.27%).
  - The estimates derived from both the total dataset (including the major cities) and the representative dataset (excluding them) show a high level of consistency, even if gender is considered. Despite regional variations in swinger density across counties, these differences seem to balance out when the larger population segments are considered. The nearly identical estimates of "identified swingers" from datasets that represent 16.34% and 5.94% of the population suggest that the overall density of swingers remains stable across different areas. This consistency underscores the reliability of the extrapolations and the robustness of the study’s methodology.

# D. Events and Classification

## Table S15: List of Events

| Number | Venue (screen name of place)  [Screen name of organizer responsible for the event] Place (municipality, state[[4]](#footnote-5)) Admission Policy,[[5]](#footnote-6) if specific | Short Description  Unless otherwise stated, all events include food and drinks without extra payment.  The brief description is written by the author of the dataset on the basis of the information provided on Joyclub by the organizers. | Classification[[6]](#footnote-7)  Citation  Entrance fee woman / man / couple[[7]](#footnote-8)  (Joyclub premium discounted[[8]](#footnote-9) price in brackets)  *for URL links and long references refer to the separate table of event-related online sources* |
| --- | --- | --- | --- |
| Date: September 19, 2023 | | | |
| 1 | Life  Gescher, NW  No restrictions. Prepayment required. | The party is named after Hamburg's traditional red-light district, St. Pauli. Guests will participate in a roleplay without predetermined roles, and each receives a set amount of play money. Although food and drinks are included in the entrance fee, guests can use their play money to obtain bonuses, such as higher-quality food. Play money cannot be exchanged for real currency; instead, guests must earn it through various playful activities. Women and men who visit the event can engage in activities like prostitution, performing in a peep show, table dancing, or attempting to profit from “illegal” gambling. Additionally, playful stations such as an employment office and a police station are set up. The description indicates that the organizers have put significant effort into designing the games, which are predominantly sexual in nature. | 3  (Life - Club für Paare, 2023c)  31 (28) / 110 (100) / 170 (160) |
| 2 | Oase  Sin City Events  Rödermark, HE  Couples and single women only | A swinger summer pool party. | 0  (Sin City Events, 2023)  49 (39) / N/A / 149 (139) |
| 3 | Steinenhaus  Hattingen, HE  Pre-registration in Joyclub or via the club homepage strictly required. Pre-registering men must have a facial picture in their Joyclub profile or submit one to the club. | The original description of this event has been significantly paraphrased to ensure clarity and sensitivity. The party is designed for women who have a preference for men with darker skin tones, as emphasized by the organizers. According to the event details, men who match this preferred appearance will greet the women at the entrance. Women also have the option to register in advance for a specific part of the event where they can engage in sexual activities with several men who meet these criteria. It is emphasized that no spectators will be allowed in the designated room, ensuring that only active participants are present. | 2  (Steinenhaus, 2023a)  25 (20) / 90 (80) / 80 (70) |
| 4 | Equinoxe  Hamburg, HH | A swinger party. Latin American music is played, and drinks from that region are offered. | 0  (Club Equinoxe, 2023a)  35 / 75 / 85[[9]](#footnote-10) |
| 5 | Beach Club St. Tropez  Affing, BY  Not directed towards unaccompanied women  Men must register anonymously (the club can see the profiles also after an anonymous registration) | Visitors to this event, called “Mare Riding,” must adhere to a strict dress code. Gentlemen are required to register in advance using Joyclub’s anonymous registration function, ensuring that the participating women do not know which gentlemen will be present. The women complete a form detailing their sexual preferences and limits. Once the women are blindfolded, the gentlemen are allowed to enter the club. The blindfolded women are then presented to the gentlemen, and, under the moderation of their partner or club staff, they engage in sexual activities with several gentlemen selected by their dominant companion. The gentlemen are required to strictly follow the previously stated preferences and boundaries, wheras a “no” has to be respected as a firm “no.” | 1  (Beach Club St. Tropez, 2023)  40 (40) / 150 (150) / 120 (120) |
| 6 | Angel of Fantasy  Untermeitingen  Couples (unclear how solo women were admitted)  Pre-registration through Joyclub mandatory | A swinger party for couples, emphasizing an orientation towards bi-sexual women. | 0  (Angel of Fantasy, 2023)  25 (20) / N/A / 110 (100) |
| 7 | Tempeloase  Großbeeren (BB)  Couples and solo women only  Age limit 45 years  Pre-registration with a profile bearing a photo (or submission of photo) encouraged | A swinger party for couples and solo women, with an age restriction to 45 years for all attendees. The organizers reserve themselves the right to grant exemptions to this rule. | 0  (Tempeloase, 2023a)  60 (50) / N/A / 110 (100) |
| 8 | Club 2020  Malsch, BW  No restrictions | A swinger party.The club celebrates its second anniversary. | 0  (Club 2020, 2023)  40 (30) / 130 (120) / 120 (100) |
| 9 | Port of Senses  Karlsruhe, BW  No dominant men.  Strict dress code. Leisure wear as well as business suits are not allowed. | The Open Relationship event caters dominant women. Participating men are blindfolded and presented to attending dominant female guests who are then encouraged to touch, interrogate, and use the men for sexual pleasure. | 2  (Port of Senses, 2023)  50 (40) / 100 (90) / 100 (90) |
| 10 | Savannah  Wiedemar, SN  No restriction. | A swinger party. The music concentrates on Schlager[[10]](#footnote-11) and Discofox[[11]](#footnote-12) genres. | 0  (Club Savannah, 2023a)  65 (55) / 135 (125) / 135 (125) |
| 11 | Pärchenclub Schiedel  Kamenz, SN  No restriction. | A swinger party. Uniforms are encouraged for men, although not required for admission. | 0  (Pärchenclubhotel Schiedel, 2023b)  30 (20) / 120 (110) / 90 (80) |
| 12 | Schloss Milkersdorf  Kolkwitz, BB  Couples only[[12]](#footnote-13)  Prepayment strongly encouraged; admission of guests without prepaid tickets only upon availability. | The party description emphasizes the exclusivity of the food, beverages, and the location itself. Even the playrooms, where sex is permitted, are mentioned only in terms of the music played there. | 1  (HotnDirtyEvents / HnD, 2023a)  N/A / N/A / 170 (170) |
| 13 | FFF Extasia  München, BY  No restrictions. | A swinger party with non-mandatory “school” dress code. | 0  (FFF-Extasia, 2023)  40 (30) / 110 (100) / 120 (110) |
| 14 | Casa No. 1  Chemnitz, SN  No restrictions. | A swinger party with an “Oktoberfest” theme, featuring corresponding catering such as beer and sausages. Bavarian outfits are encouraged but not mandatory. | 0  (Casa No.1, 2023)  40 (35) / 105 (95) / 105 (85) |
| Date: September 23, 2023 | | | |
| 15 | QATER 1  Köln, NW  No restrictions. | A Schlager[[13]](#footnote-14) party focusing on the music and partying. | 1  (Fun´n more Events, 2023)  31 (28) / 31 (28) / 62 (56) – Drinks not included |
| 16 | Steinenhaus  Hattingen, NW  No restrictions. | A swinger party focused on women with significantly above-average body weight and the men who appreciate these characteristics. The party is described as being hyperactive. The term *molly*, used in the party description, is an anglicized variation of the German word “mollig.” In German, “mollig” not only carries a positive connotation of “overweight” but can also mean “cozy” or “pleasantly warm,” depending on the context. | 2  (Steinenhaus, 2023c)  30 (25) / 100 (90) / 80 (70) |
| 17 | Feuer und Eis  Bruchsal, BW  No restrictions.  Pre-registration strictly required.  Accompanied solos are required to submit certain data or the nickname related to the Joyclub profile of the accompanying person. | A swinger party with no formal age restrictions, but with a focus on younger attendees. Individuals up to age thirty-three, or couples whose combined ages do not exceed sixty-six, are granted discounted entrance fees. | 0  (Feuer und Eis, 2023b)  25 (20) / 110 (100) / 80 /70) |
| 18 | Oase  Rödermark, HE  Couples and solo females only. | This event is part of a series celebrating the club's 30th anniversary. Participants are asked to wear stylish attire in the lower part of the club, which includes the dance floor, bar, and food area. For men, this means wearing a suit and classic shirt or long dress pants with a neat shirt, as well as closed smart shoes. Kinky outfits in PVC, leather, or latex with matching footwear are also welcome. For women, options range from a little black dress to sexy party outfits or kinky styles to lingerie. Additional suggestions for women include corsets, lace, suspender outfits, PVC, leather, latex, high heels, or sexy shoes. In the upper area, known as the experience area, which is more focused on sexual activity, the dress code does not apply. From 11 p.m., typical clubwear for swingers is also permitted in the lower area. | 0  (DIE OASE, 2023)  49 (44) / N/A / 149 (139) |
| 19 | Grace & Beauty  Wörrstadt, RP  No restrictions. | A swinger party labeled as a summer closing event. The music is techno-oriented, and it is emphasized that sex is permitted on the dance floor. The club recommends a nearby hotel that offers a shuttle service to and from the party venue. Guests are encouraged to inform the hotel that they will be attending the party, allowing the hotel to accommodate them in rooms close to one another, making it easier for guests to meet in the hotel rooms for additional sex after the party. | 0  (Grace & Beauty, 2023)  50 (40) / 125 (115) / 130 (120) |
| 20 | Beach Club St. Tropez  Wilder Süden  Affing, BY  No restrictions. Dress code (shirt and pants for men, no blue jeans) applies. | The party features two themes. The first theme is casino-related, where guests can gamble in games like poker using play money that cannot be exchanged for real currency. The second theme is inspired by the Hamburg red-light district of St. Pauli (see party number 1), where guests can offer sexual services in exchange for play money. The play areas include a couples-only section and a darkroom setting. The event will feature erotic shows, opportunities for rough sex, swinger settings with a surplus of men, and “gangbang” scenarios. | 2  40 (30) / 130 (120) / 119 (109)  (Wilder Süden, 2023) |
| 21 | Equinoxe  Hamburg, HH  No restrictions. Fetish dress code applies. | The party is focused on fetishes, although the description does not specify particular ones, leaving it up to the guests to define their own. The club encourages guests to communicate their personal fetishes to the staff via Joyclub messages well in advance of the party. This allows the club to set up a dedicated play area where guests can act out these fetishes during a limited time window. | 2  (Club Equinoxe, 2023b)  35 / 75 / 85[[14]](#footnote-15) |
| 22 | Faro 8  Marl, NW  No restrictions. | A swinger party. The organizers offer a prostitution game using play money, which cannot be exchanged for real currency. Each solo man receives ten play coins, and each couple receives five at the start of the event. Women can offer sexual services in exchange for these coins through free negotiations, auctions, on a per-minute basis, or as a living buffet. The women can either deposit the coins they earn into a play bank or use them to receive sexual services from others in exchange. | 2  (Faro 8, 2023a)  20 (15) / 90 (80) / 80 (70) |
| 23 | Villa Party  Rellingen, SH  Couples and solo women only.  Admission only on the basis of Joyclub accounts with photos. Partial prepayment required. | A swinger party. The club emphasizes the food buffet, the selection of drinks, the DJs, and the effort it takes to convert the venue into a swingers’ club for each party. | 0  (Villa Party, 2023)  50 (45) / N/A / 100 (90) |
| 24 | SunMoon  Sachsenheim, BW  No restrictions. Confirmed pre-registration required from all. Women do not pay an entrance fee to this event but are required to submit a non-anonymous registration from a Joyclub profile with photo. | The event is planned as a swinger party with an extreme surplus of men. A moderation by the organizers or games are not announced. | 0  (SunMoon, 2023b)  0 (0) / 100 (90) / 73 (65) |
| 25 | Malibu Stars  Greven, NW  No restrictions. | The party motto relates to the celebration of the fifth anniversary of the club. A DJ is announced. | 0  (Malibu Stars, 2023)  30 (25) / 90 (80) / 90 (80) |
| 26 | Cäsar’s Palace  Sprockhövel, NW  No restrictions. | The party has a 1990s theme, with the music selection reflecting that era.  The description encourages guests to have sex throughout the club, with the exception of the pool and the buffet areas. | 0  (Cäsars Palace, 2023a)  25 (20) / 110 (100) / 70 (60) |
| 27 | Tempeloase  Großbeeren, BB  No restrictions. | The theme of the party is the end of summer. The description encourages couples to seek variety, stating that the event is ideal for women who find one man far from enough. The party is not suitable for those who consider themselves “cuddly swingers.” | 0  (Tempeloase, 2023b)  10 (0) / 70 (60) / 60 (50) |
| 28 | Life  Gescher, NW  Couples and solo women. | Colored glowing bracelets are distributed at the party, allowing guests to express their preferences and streamline communication. Red bracelets indicate that the wearer initially just wants to observe without engaging in sexual activities with others. Yellow bracelets signal a willingness to swap partners without intercourse, whereas green bracelets indicate a willingness to swap partners, including intercourse. A blue bracelet signifies a bisexual inclination, and the pink bracelet is understood as a simple invitation to “use me.” Guests are free to change the colors of their bracelets at any time. No moderation of the event or special games are scheduled. | 0  (Life - Club für Paare, 2023a)  65 (55) / N/A / 130 (120) |
| 29 | Chateau Royal  Bad Honnef, NW  Couples and women only. No admission to accompanied men registering through a Joyclub solo profile. | Colored glowing bracelets are distributed at the party. Based on the colors, the guests can express their preferences to streamline their communication. The color codes have the same meaning as in party number 28.  The guests are free to change bracelet colors at any time. | 0  (Chateau Royal, 2023)  35 (30) / N/A / 105 (95) |
| 30 | Club Passion  Stuhr, NI  No restrictions. | A swingers party. Music from the 1980s to the 2000s is played. Visitors can win one of three vouchers for the club, with the value of €150, €100, or €50, respectively. | 0  (Club Passion, 2023a)  25 (22) / 90 (81) / 75 (67) |
| 31 | FFF-Extasia  [YourNitelife]  München, BY  Couples and solo women only. Maximum age of all guests 45 years.  Registration with photo, in the Joyclub profile or separately submitted to the organizers, required. | The swinger party focuses on guests in the age group between 30 and 35 years. | 0  (YourNITELIFE, 2023b)  45 (40) / N/A / 130 (120) |
| 32 | Location One  Menden, NW  Couples and solo women only. | The motto of the swinger party is based on the owner’s birthday. | 0  (Location One, 2023)  15 (10) / N/A / 89 (79) |
| 33 | Club Karee  [eyes-touch fashion&events]  Philippsburg, BW  Generally, couples and solo women only. A few solo men, whom the club says are carefully selected, are additionally admitted. | The dress code in white reflects the party theme, which focuses on the Balearic Islands of Ibiza and Mallorca. In line with the party’s theme, the food offerings include tapas and olives, and cocktails typical of the Balearics are served. The music selection also centers on music currently popular in Balearic clubs. Admission for couples begins an hour before that of solo guests, allowing couples to engage in undisturbed sex with each other at the start of the party. The organizers emphasize that the party is not anonymous but is also intended as an opportunity to get to know each other and make new acquaintances. According to the description, it is geared toward swingers who enjoy communication. | 0  (eyes touch de, 2023)  30 (20) / 100 (90) / 85 (75) |
| 34 | Villa Illusion  [SNA Event Concept]  Euskirchen, NW  No restrictions. | The motto "Alf Layla waLayla" refers to Thousand and One Nights, of which it is the Arabic language version. The décor and show interludes refer to the Orient, and hookahs and mint tea are also served. | 0  (SNA Event Concept, 2023)  30 (20) / 85 (75) / 95 (85) |
| 35 | Secret Lions  Lingen, NI  No restrictions. | The party is characterized by the use of a foam cannon that covers the dance floor in the club's conservatory with foam. Guests are encouraged to wear minimal clothing and show a lot of skin. Street clothes are strictly prohibited. Swimwear is not mentioned among the clothing suggestions. Guests are reminded that they are not allowed on the play areas while covered in foam. | 0  (Secret Lions, 2023)  33 (28) / 95 (85) / 95 (85) |
| 36 | Club Savannah  Wiedemar, SN  Couples and solo women only. According to the organizers, five selected solo men will be admitted. | A swinger party with an “Oktoberfest” motto. In line with the theme, Bavarian finger food and beer will be served. Wearing traditional Bavarian clothing is not obligatory. | 0  (Club Savannah, 2023b)  60 (50) / 120 (110) / 120 (110) |
| 37 | Angel of Love  [Hotwife Events]  Roth, BY  Generally only couples and solo men. Pre-registration and admission through Joyclub required. No admission for solo women whose profile does not reveal that they are in a permanent relationship. No admission for couples who are just swinger friends. Men are required to grant the organizers access to a facial picture in the Joyclub profile. Persons who had ever cancelled a registration to a prior event of the organizers are only admitted upon prepayment, which is non-refundable in case of cancellation of the guest. | This party is directed at couples who practice wife-sharing and cuckolding.  One section of the club consists of two aisles.  In one aisle, men may only enter if they have received a ribbon from a woman—each woman receives five ribbons at the beginning of the event, which she may distribute to men other than her partner whom she wishes to join her for sex. Once a man leaves this ribbon-controlled area, his ribbon is taken from him to prevent re-entry. Male partners of the women in this area are not allowed to follow their female partners or the men with ribbons, ensuring that they do not witness their partner having sex with other men.  The other aisle is reserved as a shared area for women, their partners, and solo men. Here, male partners can watch their female partners having sex with other men.  Additional play areas in the club do not have these restrictions. | 2  (Hotwife events, 2023)  20 (10) / 90 (70) / 75 (65) |
| 38 | Club 2020  [Scandal Bizarre]  Malsch, BW  No restrictions.  Prior registration through Joyclub required. The organizers must be given access to facial photos. | The party is aimed at people with various, broadly defined fetishes. Newcomers are strongly encouraged to sign up. The event will feature a freestyle competition for the most outrageous party outfit, followed by a Open Relationship show. The organizers emphasize that, unlike other Open Relationship and fetish events, the focus of this party will be more on games and sex, with less emphasis on dancing. | 2  (Scandal Events, 2023)  40 (35) / 100 (90) / 110 (100) |
| 39 | Club Hemmungslos  Hannover, NI  Age restriction: Solos may not be older than 35 years. Of couples, one part may be older than 35, but not older than 45 years, and the sum of ages of the partners may not exceed 80 years. Solo women below 30 years of age are granted free entry. | Apart from the age restriction, distinctive features of this swinger party are not described. | 0  (Club Hemmungslos, 2023)  30 (20) / 80 (70) / 70 (60) |
| 40 | Michas Swingertreff  Lehrte, NI  Couples and solo women only. | No specific party features defined. Beginners are specifically encouraged to register for this event. | 0  (Michas Swingertreff, 2023a)  25 (20) / N/A / 65 (55) |
| 41 | Schloss Milkersdorf  Kolkwitz, BB  Couples and solo women only. | A swinger party.  The organizers encourage guests to wear masks with a color code: a black mask indicates that the guest is ready and willing to engage in sexual activity with others, whereas a white mask signifies that they are not. The organizers also emphasize the requirement to dress elegantly. | 0  (Schloss Milkersdorf, 2023)  95 (85) / N/A / 190 (170) |
| 42 | Fetisch Hof Berlin  Berlin, BE  No restrictions. | The event is a fall backyard party featuring live music, a bondage demonstration, and a spanking show. A barbecue takes place, and a cake buffet is offered in the afternoon. Tents are set up in the outdoor area of the courtyard. Guests can participate in a vibrator race to win prizes. Admission to the festival and market is free. Visitors can also engage in Open Relationship play or other forms of sexual activity in the Open Relationship lounge, which is part of the courtyard, starting from 2:00 p.m. For the evening event, guests require an admission wristband, which can be purchased at the festival. Admission for the evening event is limited to sixty persons. | 2  (Fetisch Hof Berlin, 2023)  0 (0) / 0 (0) / 0 (0) |
| 43 | EL BRASI Sex Film Club  Bochum, NW  No restrictions. Single men require advance admission after a Joyclub registration, where a profile which matches requirements is needed. | The venue is a porn cinema. The event begins with a non-fiction author reading from his book about cuckolding. Regarding the party that follows, the organizers emphasize that the wishes of the female guests are prioritized. Female guests have the option to engage in sexual activity in larger play areas but may also use a lockable area, provided it is used together with at least six men. | 0  (EL BRASI Sex Film Club, 2023a)  10 (10) / 25 (25) / 20 (20)  Food and drinks are not included. |
| 44 | SF Lounge  Kaarst, NW  Couples and solo women only. | Colored glowing bracelets are distributed at the party, allowing guests to express their preferences and streamline communication. Red bracelets indicate that the wearer prefers to observe at first, without engaging in sexual activity with others. Yellow bracelets signal a willingness to swap partners without intercourse, whereas green bracelets indicate a willingness to swap partners, including intercourse. A blue bracelet signifies that sex with others is explicitly intended, including group sex. The color pink indicates a bisexual inclination. Guests are free to change their bracelet colors at any time. No moderation of the event or special games are scheduled. | 0  (SF Lounge, 2023a)  40 (35) / N/A / 85 (75) |
| 45 | La Huître  Markgröningen, BW  No restrictions. | A swingers party. Music from the 1980s is played. | 0  (La Huître, 2023)  10 (5) / 110 (100) / 80 (70) |
| 46 | Angels of Fantasy  [REAL YOUNGSTERS]  Untermeitingen, BY  Couples and bi-sexual women only. Strict blocked-list policy directed against no-shows of registered persons.  Age restriction: The average age of couples should be between 35 and 37 years, and not beyond 45 years. Solo women have access up to an age of 45 years. | A swingers party. Color code bracelets, indicating the intentions of the visitors, are distributed. The color code itself is not explained in the party explanation on Joyclub. | 0  (REAL YOUNGSTER, 2023)  30 (25) / 90 (80) |
| 47 | Dreamlight  Nürnberg, BY  No restriction. Solo men were warned that they would not feel comfortable if they are not bisexual. | The event is targeted toward bisexual women, bisexual men, gay and lesbian individuals, transvestites, transgender people, transsexuals, male lingerie wearers, and crossdressers. Persons, particularly heterosexuals, who do not share any of these characteristics or preferences but are comfortable interacting with those who do, are also welcome.[[15]](#footnote-16)  In addition to the mixed play areas, separate play areas are set up, each of which only men or only women are allowed to enter. The area for men has the character of a darkroom. Colored wristbands are handed out: a white wristband indicates that the wearer is interested in same-gender interactions but has no prior experience, while a green wristband indicates interest in same-gender sex and familiarity with it. Those who do not wear a wristband indicate that they are not interested in same-gender acts. | 2  (Dreamlight, 2023b)  25 (20) / 110 (100) / 80 (70) |
| Date: October 7, 2023 | | | |
| 48 | Life  Gescher, NW  Couples and single women only. Advance registration and payment, three days before the event at the latest, required. | A swinger party with an “Oktoberfest” motto. Wearing traditional Bavarian clothes is mandatory. Bavarian food and beer are served.  A live band plays.  The event starts at 4 p.m. in the afternoon and ends late in the night. | 0  (Life - Club für Paare, 2023b)  80 (70) / N/A / 165 (155) |
| 49 | fetischhaus  Wiesbaden. HE  No restrictions. | The party is aimed at an audience that appreciates Open Relationship, as well as the PVC, leather, and latex style. Additionally, persons who do not intend to play but only wish to party are expressly welcome. The music set focuses on Depeche Mode, 1980s music, electro and gothic rock, electronic body music, and dark wave. The event description also provides detailed information about the Open Relationship-oriented play opportunities at the club and its corresponding facilities, along with a request not to disturb participants in the play areas with loud conversations. | 2  (fetischhaus, 2023)  55 (45) / 110 (100) / 120 (100) |
| 50 | Quicky Rhein-Neckar  [Lifestyles GmbH]  Weinheim, BW  No restrictions. | A swinger party. No age restriction. Young men up to age 40 are intended to be attracted by a reduced entrance fee of €2 per year of life. The party is intended to cater women who appreciate sex with younger men. | 0  (Lifestyle GmbH [Quicky Rhein-Neckar], 2023)  30 (25) / 120 (110) / 99 (89) |
| 51 | Steinenhaus  Hattingen, NW  Couples only. | A swinger party. Announced as an active party where partner swapping, including intercourse, is appreciated. At 9:00 p.m., the first six couples that actively engage in sex on the dancefloor are given a surprise. | 0  (Steinenhaus, 2023b)  N/A / N/A / 90 (80) |
| 52 | Club Equinoxe  Hamburg, HH  No restrictions. Anonymous registrations are not accepted. | A swinger party. The party is themed “Ibiza,” which is especially reflected in the which is particularly reflected in the selection of cocktails, which are included with the entrance fee. Near the dance floor, male dancers hired by the club perform in cages. The event description highlights the equipment of the dance area and the catering facilities. It also emphasizes that no exceptions are allowed regarding the dress code. The dress code for women is: hot, sexy, or a little black dress; for men: PVC, leather, fetish, or evening attire. It is also emphasized that there will be no surplus of men. | 0  (Club Equinoxe, 2023c)  40 (35) / 100 (90) / 85 (75) |
| 53 | DerCult – nightclub&more  [Tabou Events]  Nürnberg, BY  No restrictions. Strict dress code. | A dance event with small play areas. Drinks and food are not included. The DJ is an owner of the Schloss Milkersdorf venue in Kolkwitz, BB. Additionally, there will be live music. The party is themed around a “revolution” of hedonists of all stripes, united in rebelling against sexual repression and prudery, who have founded a “República del Hedonismo.” Dress code themes include end times, Matrix, uniform, steampunk, cybergoth, PVC, leather, latex, wet look, costumes and fantasy, lingerie, body painting, and burlesque. Persons in everyday clothes are excluded from admission. | 1  (Tabou Events, 2023)  20 (18) / 27 (24) / 47 (42) |
| 54 | Feuer und Eis  Bruchsal, BW  Couples and solo women only. | A swinger party. Colored luminous arm bracelets are issued to enable guests to show their preferences to everyone. | 0  (Feuer und Eis, 2023a)  35 (30) / N/A / 90 (80) |
| 55 | Saphir  [YourNITELIFE]  Leipzig, SN  Couples and solo women only.  Maximum age 45 years; may be exceptionally waived upon discretion. | A swinger party, focusing on guests with an age between 30 and 35 years. The description does not demonstrate any specific features. | 0  (YourNITELIFE, 2023a)  65 (55) / N/A / 150 (140) |
| 56 | Big Bamboo  [WAALberg]  Koblenz, RP  Couples and single women only. | The event description plays with fetish type motives around school. The dress code relates to schools. For the women, suggestions include a schoolgirl outfit, too short skirt, too low-cut blouse, high stockings, plus sneakers / chucks, sucker, school bag, teacher outfit with strict glasses, skirt, suspenders and wide-open blouse, Alice in Wonderland style, nice sexy lingerie. According to the description, first wobbly steps on high heels are also appreciated. For men, the suggestions include a teacher/business outfit with cloth pants, ironed plain shirt and good leather shoe, sports teacher or janitor outfit, student outfit, professor, or principal outfit, or that of a submissive student with a school bag. A play suggestion relates to the story of a schoolgirl caught smoking on the schoolyard, and who faces Open Relationship punishment for this, or has to pleasure the principal sexually.  Aside from the motto and dress code, games or plays moderated by the organizers are not announced. | 0  (WAALberg, 2023a)  30 (25) / N/A / 95 (85) |
| 57 | Tempeloase  Großbeeren, BB  No restrictions. When registering, the organizer must be granted access to a facial image. | A swingers party. Participants can use brightly colored bracelets to express their preferences to other guests. According to the description, a green bracelet means that other guests should not talk much but should take the wearer with them. A yellow bracelet expresses interest in couples. A red bracelet indicates that the wearer prefers to observe first and that any initiative should come from them. A pink bracelet signals interest in bisexual activities, and a blue bracelet identifies a solo woman or man seeking another solo person. | 0  (Tempeloase, 2023a)  10 (0) / 95 (85) / 95 (85) |
| 58 | Club Passion  Stuhr, NI  No restrictions. Prior registration required. | A swingers party. Newcomers can arrive between 6:30 p.m. and 7:30 p.m. During this time, the club and its procedures are explained to them, and they can ask questions. All other guests are asked not to show up until 8:00 p.m. at the earliest. Colored glowing bracelets are given out to guests, which they can use to signal their preferences to others. A blue wristband indicates that it is the wearer's first visit and that they intend to look around and talk to others initially. According to the description, this wristband is suitable for visitors who just want to have a friendly conversation first. A yellow bracelet shows that the wearer is open to new experiences and curious; while touching is fine, they do not pursue partner swapping. A green bracelet signals to other guests that almost anything is possible if there is mutual sympathy, including partner swapping. A pink bracelet indicates that the wearer has a bisexual inclination or is bi-curious and would like to explore this. A red bracelet specifically shows disinterest in solo men, with a preference for contact with couples or solo women. The colors can be combined and changed during the event. | 0  (Club Passion, 2023b)  25 (22) / 90 (81) / 75 (67) |
| 59 | Faro 8  Marl, NW  No hard restrictions. Admission limited to 170 guests; members of a local Open Relationship society preferred; no swingers without Open Relationship inclination. | A Open Relationship party, which is announced as a closed setting of a local Open Relationship society, but open to other interested persons.  A play slave auction is announced as part of the program, where play money is used.  The play money cannot be converted from or into real currency. Non-Open Relationship audience is not appreciated. | 2  (Faro 8, 2023b)  45 (38) / 100 (90) / 100 (90) |
| 60 | Dreamlight  Nürnberg, BY  Couples and solo women only. | A swingers party. Newcomers can take a tour of the club between 7:00 p.m. and 8:00 p.m., during which their questions are answered. They can then decide if they want to stay for the party. All other guests are asked to arrive only at 8:00 p.m. Colored glowing bracelets serve the purpose of signaling the wearer’s preferences to other guests. A red bracelet indicates that the wearer is new and not looking for contact initially. An orange bracelet means that there is interest in couples or ladies, but the wearer prefers to initiate contact at a slow pace. A green bracelet, on the other hand, indicates that full activity is desired when the wearer meets the right person or persons. | 0  (Dreamlight, 2023a)  35 (30) / N/A / 110 (100) |
| 61 | FFF-Extasia  [YourNITELIFE]  München, BY  Couples and solo women only.  Maximum age 45 years; may be exceptionally waived upon discretion. | A swinger party, focusing on guests aged between 30 and 35 years. The description does not demonstrate any specific features. | 0  (YourNITELIFE, 2023c)  45 (35) / N/A / 125 (115) |
| 62 | Beach Club St. Tropez  [WAALberg]  Affing, BY  No restrictions. Prior registration required. When registering, the organizer must be granted access to a facial image. | A swinger party. Buffet food described in detail. Guests can claim promo prices at a nearby hotel when mentioning the club in the booking. Women receive a promotion bag with items worth €55. | 0  (WAALberg, 2023b)  60 (60) / 150 (150) / 150 (150) |
| 63 | Catonium  [Living Dead Production]  Hamburg, HH  No restrictions. Prior registration and prepayment recommended. Extremely strict dress code. | A Open Relationship and fetish-oriented play party with some show acts. The dress code is strict. According to the description, any clothing in that one could appear at a company or family party is excluded. Thus, for men, suit, or pants with a white shirt, for example, are not permitted. Jeans and sneakers are not allowed in any case. Clothing such as latex, PVC, leather, lace, high heels, bondage, corsages, masks, uniforms, fantasy, burlesque, gothic erotic, drag, cosplay, glamorous, kinky, or modern primitive is desired. | 2  (Living Dead Production, 2023)  49 (49) / 49 (49) / 98 (98) |
| 64 | Cäsars Palace  Sprockhövel, NW  No restrictions. | A swinger party, which, according to the description, is directed at whom the organizers call evil girls. No specific features described. | 0  (Cäsars Palace, 2023b)  25 (20) / 110 (100) / 70 (60) |
| 65 | SunMoon  Sachsenheim, BW  No further restrictions, but admission only for persons who appreciate MFM constellations. Prior registration strictly required. | A party focusing on persons who prefer a MFM constellation where at least one of the men is of dark skin complexion. Men with dark skin complexion who do not register anonymously receive a discount.[[16]](#footnote-17) The organizers use the tag #sexagainstracism. They expect the guests to be a cosmopolitan audience that has no fear of contact with foreign skin, culture, and language. The description does not relate the term “foreign” to the complexion of the skin. | 2  (SunMoon, 2023a)  30 (25) / 100 (90) / 80 (70) |
| 66 | Club Hemmungslos  [Open Relationship GO]  Hannover, NI  No restrictions. | A Open Relationship event for which additional Open Relationship equipment is set up in the club. The event is explicitly also for newcomers, who are encouraged to ask questions. The dress code excludes the usual swinger clubwear. | 2  (Open Relationship GO, 2023)  25 (20) / 80 (70) / 70 (60) |
| 67 | SF Lounge  Kaarst, NW  Couples and solo women only. | A swinger party for couples and solo women. | 0  (SF Lounge, 2023b)  40 (35) / N/A / 85 (75) |
| 68 | Angel of Love  [GENERATION FUN Events]  Roth, BY  Couples and solo women only. Age restriction applies. Solo women may not be older than 34 years. Couples may not be older than 34 years in average, and no person may be older than 43 years. Confirmed pre-registration strictly required. | A swinger party directed at an audience below 35 years of age. The description does not reveal specific features. | 0  (GENERATION FUN Events, 2023)  40 (35) / N/A / 95 (85) |
| 69 | Lillith  Puchheim, BY  Couples and solo women only. | The swinger event is aimed specifically at newcomers. They are provided with swinger mentors on request, who show the club and explain what is happening. | 0  (Lillith, 2023)  50 (40) / N/A / 130 (120) |
| 70 | Villa Illusion  [sXXXtasy]  Euskirchen, NW  No restrictions. | A swinger party which is focused on women which are called curvy by the organizers. | 2  (sXXXtasy, 2023)  30 (25) / 85 (75) / 85 (75) |
| 71 | Loft54  Rheinstetten, BW  Couples and solo women only. Confirmed pre-registration required. The organizers must be given access to facial photos. | The swinger event is the opening party of a new club. The new club is described as modern, unique, and stylishly designed, with an industrial look. Changing areas, SM rooms, a bar area, a restaurant, a dance floor, many game rooms, a wellness area, a terrace, a rooftop penthouse, and a V.I.P area are accessible for all guests.  An option can be booked, according to which the premises could be used further after the end of the party at 4.00 a.m., namely until 10.00 a. m. The then booked afterparty then takes place in the rooftop penthouse. Alternatively, an overnight stay on the playgrounds in Loft54 is possible if the additional option is booked. This additional booking is only granted to fifty people, at an additional cost per person of €60. During the extended party time, the food and drinks are still accessible without additional payment for the people who have booked the additional option. | 0  (Loft54, 2023)  40 (30) / N/A / 110 (100) |
| 72 | Schloss Milkersdorf  Kolkwitz, BB  Couples and solo women only. Prepayment strictly required. | A swinger party. The dress code is strict. For orientation, guests should imagine that they are going to a fine dining restaurant, or a theater. The women should wear, for example, a ball gown, a sexy jumpsuit, an elegant pantsuit, or the very classic little black dress. Somewhat more erotic clothing should be in style. Men are expected to appear in a tuxedo or suit. It is also possible to wear suit pants with a noble long-sleeved shirt and matching shoes. Only on the upper floor it is allowed to be more revealing – according to the description, the focus should be set on being stylish. A nude suite on the upper floor may only be entered unclothed, although women are allowed to wear lingerie there. | 0  (Schloss Milkersdorf, 2023b)  100 (90) / N/A / 200 (180) |
| 73 | Club KARREE  Philippsburg, BW  No restrictions. Confirmed registration via Joyclub required. Organizers must be granted access to facial photos. Accompanied solos are required to indicate the nickname of the Joyclub profile of the accompanying person. | This “molly” party (chubby, see the description of party number 16 for further details) bears an “Oktoberfest” motto, so Bavarian dishes are served. Wearing Bavarian traditional clothes is encouraged, but not mandatory. | 2  (Club KARREE, 2023)  30 (25) / 100 (90) / 85 (75) |
| 74 | Doro  Hof, BY  Couples and solo women only. Anonymous registrations would not be confirmed. | A swinger party with a dress code according to which all pieces worn had be white. Only shoes were allowed to have a color other than white. | 0  (DORO, 2023)  60 (55) / 150 (140) |
| 75 | Club-Puzzles  Hamburg, HH  No restrictions. | A swinger party organized by a local group of female swingers with big breasts. Female guests are encouraged to wear clothes that reveal much of their breasts. They can choose to wear a blue bracelet, which indicates general consent to be touched at their breasts by all other visitors without the requirement of being asked before. That general consent would only relate to the breasts, not to other body parts. | 0  (Club-Puzzles, 2023)  30 (20) / 90 (80) / 70 (60) |
| 76 | Beach Club Cologne  [Calvin Kleinen]  Dormagen, NW  Age restriction up to 45 years for all guests. | A swinger party with an age restriction of 45 years. | 0  (Calvin Kleinen, 2023)  40 (40) / 150 (150) / 100 (100) |
| 77 | Michas Swingertreff  Lehrte, NI  Couples and solo women only. | A swinger party for couples and solo women. No specific features described. | 0  (Michas Swingertreff, 2023b)  25 (20) / 65 (55) |
| 78 | Studio 52  Hanau, HE  No restrictions. | A swinger party. No specific features described. | 0  (Studio 52, 2023)  30 (20) / 90 (80) / 50 (40) |
| 79 | RagaZza.Club  Rehlingen-Siersburg, SL  Couples and women only. | A swinger party for couples and solo women. No specific features described. | 0  (RagaZza.Club, 2023)  20 (10) / N/A / 75 (65) |
| 80 | Clubhotel Ollywood  Radebeul, SN  No restrictions. | The event is themed around the club’s 23rd anniversary. The club advertises extensive playgrounds for sex, a wellness area, and a pool equipped with a counter-current system, which is located next to the lounge and bar. Swinger mentors are available to newcomers. Very high-priced alcoholic drinks are not included in the admission price. | 0  (Clubhotel Ollywood, 2023)  35 (25) / 105 (95) / 100 (90) |
| 81 | Burg Ibiza  Fürstenfeldbruck, BY  No restrictions. | The motto of the swinger party relates to the end of the summer period. Specific features are not mentioned. | 0  (Burg Ibiza, 2023)  50 (40) / 130 (120) / 90 (80) |
| 82 | Oase  Rödermark, HE  Couples and solo women only. Mild age restriction to 55 years for solos and 110 years, ages summed up, for couples. Strict dress code | A swingers party. The first part of the evening is characterized by a casino setting, where roulette can be played at a roulette table with a real croupier and using play money. A starting capital of chips is given out. Further tokens can be acquired through sexual acts, starting with the removal of clothing, and ending with open sexual intercourse. Up to this point, a dress code applies; clothing must be appropriate to what is worn in a casino. At midnight, the winners of the roulette game are announced. Chips are not purchased for real currency and cannot be exchanged back to real currency. After midnight, the dress code is relaxed, which means that normal swingers club dress can be worn. Three partner hotels in the vicinity offer shuttle services for guests if it is specified at the time of booking that Club Oasis is to be visited. | 2  (HotnDirtyEvents / HnD, 2023b)  49 (44) / N/A / 149 (139) |
| 83 | Pärchenclubhotel Schiedel  Kamenz, SN  No restrictions. | The swinger party has an “Oktoberfest” motto. No further information is provided in the description. | 0  (Pärchenclubhotel Schiedel, 2023a)  30 (20) / 120 (110) / 90 (80) |
| 84 | 1000 qm Fetischlokation  [O-Events]  Restricted to persons who adhere to the role play in line with the “Story of O” (Réage, P. (1973). *Story of O*. Ballantine Books.). | The role play fetish event relates to the “Story of O” by Réage (1973). The dress code and behavior of both men and women attending should conform with the story of that book, which involves that attendees perform certain actions, as described in more detail. | 2  (O-Events, 2023)  20 (20) / 110 (100) / 90 (80) |
| 85 | Swingerclub Harz-Heide  Vechelde, NI  No restrictions. Block-list policy for registered persons who do not show up is applied. | A “molly” swinger party (chubby, see the description of party number 16 for further details). Solo men receive a discount and pay only €65 if they appear between 8:30 p.m. and 9:30 p.m. | 2  (Swingerclub Harz-Heide, 2023)  25 (20) / 85 (75) / 70 (63) |
| 86 | Swingerpark Extra-3  Bad Nenndorf, NI  No restrictions. | The swinger party has an “Oktoberfest” theme. Accordingly, Bavarian food and beer is served. Appearance in traditional Bavarian clothing is not obligatory. Nevertheless, those who wear these clothes receive a €10 discount on the entrance fee. | 0  (Swingerpark Extra-3, 2023)  25 (15) / 105 (95) / 99 (89) |
| 87 | EL BRASI Sex Film Club  Bochum, NW  No restrictions. | The venue is a porn cinema. Attending women have the option to engage in sexual activity in larger play areas but can also take advantage of a lockable area together with at least six men. | 0  (EL BRASI Sex Film Club, 2023b)  10 (10) / 25 (25) / 20 (20)  Drinks and food are not included. |
| 88 | XtraJOY Augsburg  Augsburg, BY  No restrictions. | The venue is a porn cinema. No specific features described. | 0  (XtraJOY Augsburg, 2023)  0 (0) / 25 (25) / 0 (0)  Drinks and food are not included. |

## References Related to the Footnotes in Table S15

IDO (2023). IDO Dance Sport Rules & Regulations. In *ido-dance.com*. Retrieved October 13, 2023, from <https://www.ido-dance.com/ceis/ido/rules/competitionRules/2023-IDO-Rule-Book.pdf>

Joyclub. (n.d.-a). *Was kostet ein Unternehmensprofil?* [What are the costs of an enterprise profile?]. Retrieved October 10, 2023, from <https://support.joyclub.com/hc/de/articles/4414446410258-Was-kostet-ein-Unternehmensprofil->

Joyclub. (n.d.-b). *Events - Gästeverwaltung Gesamtüberblick* [guest management, general overview]. Retrieved October 10, 2023, from [https://support.joyclub.com/hc/de/articles/12584639414290-Gesamt%C3%BCberblick](https://support.joyclub.com/hc/de/articles/12584639414290-Gesamtüberblick)

Schlager Radio (2022, August 19). *Schlager - Eine Definition* [Schlager – a definition]. Schlager Radio. <https://www.schlagerradio.de/schlager-eine-definition>

Zweites Deutsches Fernsehen. (2023, June 29). *“Layla” mitgesungen: Günther verteidigt sich. Umstrittener Partyhit “Layla”: Günther Verteidigt Mitsingen* [“Layla” sung along: Günther defends himself. Disputed party hit “Layla”: Günther defends singing along]. ZDFheute. <https://www.zdf.de/nachrichten/politik/guenther-layla-mitsingen-100.html>

## References Related to the Events Data Sources (Table S15)

Angel of Fantasy. (2023). *PAARE & BI-LADYS ABEND Swinger-Party - 86836 untermeitingen - JOYClub*. Joyclub. Retrieved September 15, 2023, from <https://www.joyclub.de/event/1432011.paare_bi_ladys_abend_untermeitingen.html>

Open Relationship GO. (2023). *Open Relationship Dark Rock Party Open Relationship- & Fetisch-Party - 30161 Hannover - JOYClub*. Joyclub. Retrieved October 14, 2023, from <https://www.joyclub.de/event/1398468.bdsm_dark_rock_party_hannover.html>

Beach Club St.Tropez. (2023). *VIP STUTENTREIBEN ROYAL VOL. I Swinger-Party - 86444 Affing - JOYClub*. Joyclub. Retrieved September 15, 2023, from <https://www.joyclub.de/event/1426498.vip_stutentreiben_royal_vol_i_affing.html>

Burg Ibiza. (2023). *SEASON CLOSING PARTY • SPECIAL PRICES Erotik-Party - 82256 Fürstenfeldbruck - JOYClub*. Joyclub. Retrieved October 14, 2023, from <https://www.joyclub.de/event/1443419.season_closing_party_special_prices_fuerstenfeldbr.html>

Calvin Kleinen. (2023). *Adam & Eva Party mit Calvin Kleinen und Mila Swinger-Party - 41540 Dormagen - JOYclub*. Joyclub. Retrieved October 14, 2023, from <https://www.joyclub.de/event/1457255.adam_eva_party_mit_calvin_kleinen_und_mila_dormage.htm>

Casa No.1. (2023). *Das Casa Oktoberfest Swinger-Party - 09113 Chemnitz - JOYclub*. Joyclub. Retrieved September 15, 2023, from <https://www.joyclub.de/event/1343739.das_casa_oktoberfest_chemnitz.html>

Cäsars Palace. (2023a). *90’ies BITCH Swinger-Party - 45549 Sprockhövel - JOYClub*. Joyclub. Retrieved September 22, 2023, from <https://www.joyclub.de/event/1389061.90_ies_bitch_sprockhoevel.html>

Cäsars Palace. (2023b). *DIE NACHT DER BÖSEN MÄDCHEN Swinger-Party - 45549 sprockhövel - JOYClub*. Joyclub. Retrieved October 14, 2023, from <https://www.joyclub.de/event/1421595.die_nacht_der_boesen_maedchen_sprockhoevel.html>

Chateau Royal. (2023). *Farben der Nacht Swinger-Party - 53604 Bad Honnef - JOYclub*. Joyclub. Retrieved September 22, 2023, from <https://www.joyclub.de/event/1425970.farben_der_nacht_bad_honnef.html>

Club 2020. (2023). *B-day Bash 2.Edition- the Place 2 be Erotik-Party - 76316 Malsch - JOYclub*. Joyclub. Retrieved September 15, 2023, from <https://www.joyclub.de/event/1425089.b_day_bash_2_edition_the_place_2_be_malsch.html>

Club Equinoxe. (2023a). *COCKTAIL, R&B & LATINO NIGHT Sonstiges - 20537 Hamburg - JOYClub*. Joyclub. Retrieved September 15, 2023, from <https://www.joyclub.de/event/1370746.cocktail_r_b_latino_night_hamburg.html>

Club Equinoxe. (2023b). *CODE UNIQUE Open Relationship- & Fetisch-Party - 20537 Hamburg - JOYClub*. Joyclub. Retrieved September 22, 2023, from <https://www.joyclub.de/event/1317511.code_unique_hamburg.html>

Club Equinoxe. (2023c). *IBIZA Night - Closing! Swinger-Party - 20537 Hamburg - JOYClub*. Joyclub. Retrieved October 14, 2023, from <https://www.joyclub.de/event/1370727.ibiza_night_closing_hamburg.html>

Club Hemmungslos. (2023). *YOUNG & HORNY Swinger-Party - 30161 Hannover - JOYClub*. Joyclub. Retrieved September 22, 2023, from <https://www.joyclub.de/event/1435439.young_horny_hannover.html>

Club KARREE. (2023). *Das Grosse Molly-Treffen XLVII “Oktoberfest” Swinger-Party 76661 Philippsburg*. Joyclub. Retrieved October 14, 2023, from <https://www.joyclub.de/event/1421150.das_grosse_molly_treffen_xlvii_oktoberfest_philipp.html> Access to the webpage only possible for members with a Joyclub membership. Free memberships qualify for access.

Club Passion. (2023a). *Back to the 80s/90s/00s Party & Tombola Swinger-Party - 28816 Stuhr - JOYClub*. Joyclub. Retrieved September 22, 2023, from <https://www.joyclub.de/event/1424981.back_to_the_80s_90s_00s_party_tombola_stuhr.html>

Club Passion. (2023b). *Newcomer -Leuchtbänder Party & DJ Mr.T Swinger-Party - 28816 Stuhr - JOYClub*. Joyclub. Retrieved October 14, 2023, from <https://www.joyclub.de/event/1430298.newcomer_leuchtbaender_party_dj_mr_t_stuhr.html>

Club Savannah. (2023a). *Die unanständige Schlager & Discofox Party!! Dance-Party - 04509 Wiedemar - JOYclub*. Joyclub. Retrieved September 15, 2023, from <https://www.joyclub.de/event/1408467.die_unanstaendige_schlager_discofox_party_wiedemar.html>

Club Savannah. (2023b). *Oktoberfest Party  frech-sexy & frivol ! Swinger-Party - 04509 Wiedemar - JOYclub*. Joyclub. Retrieved September 22, 2023, from <https://www.joyclub.de/event/1422815.oktoberfest_party_frech_sexy_frivol_wiedemar.html>

Clubhotel Ollywood. (2023). *23 Jahre Ollywood Swinger-Party - 01445 Radebeul - JOYClub*. Joyclub. Retrieved October 14, 2023, from <https://www.joyclub.de/event/1395731.23_jahre_ollywood_radebeul.html>

Club-Puzzles. (2023). *4. BigBoobs Norddeutschland Geburtstags-Party Swinger-Party - 22769 Hamburg*. Joyclub. Retrieved October 14, 2023, from <https://www.joyclub.de/event/1438639.4_bigboobs_norddeutschland_geburtstags_party_hambu.html> Access to the webpage only possible for members with a Joyclub membership. Free memberships qualify for access.

DIE OASE. (2023). *30 Jahre - Die Oase - Birthday Gala Swinger-Party - 63322 Rödermark - JOYClub*. Joyclub. Retrieved September 22, 2023, from <https://www.joyclub.de/event/1416981.30_jahre_die_oase_birthday_gala_roedermark.html>

DORO. (2023). *WHITE NIGHT - BE AN ANGEL Swinger-Party - 95030 Hof - JOYClub*. Joyclub. Retrieved October 14, 2023, from <https://www.joyclub.de/event/1394688.white_night_be_an_angel_hof.html>

Dreamlight. (2023a). *PAARE & DAMEN NEWCOMER & KNICKLICHTER Swinger-Party - 90411 Nürnberg - JOYClub*. Joyclub. Retrieved October 14, 2023, from <https://www.joyclub.de/event/1441152.paare_damen_newcomer_knicklichter_nuernberg.html>

Dreamlight. (2023b). *ROSAROT = DIE BI PARTY mit Leuchtbänder Swinger-Party - 90411 Nürnberg - JOYclub*. Joyclub. Retrieved September 22, 2023, from <https://www.joyclub.de/event/1416132.rosarot_die_bi_party_mit_leuchtbaender_nuernberg.html>

EL BRASI Sex Film Club. (2023a). *HÜ & HOT CUCKOLD SPECIAL Erotik-Party - 44879 Bochum - JOYClub*. Joyclub. Retrieved September 22, 2023, from <https://www.joyclub.de/event/1443011.hue_hot_cuckold_special_bochum.html>

EL BRASI Sex Film Club. (2023b). *HÜ & HOT Erotik-Party - 44879 Bochum - JOYClub*. Joyclub. Retrieved October 14, 2023, from <https://www.joyclub.de/event/1443449.hue_hot_bochum.html>

eyes touch de. (2023). *White IB!ZA & Mallorca ! Fiesta especial ! Swinger-Party - 76661 Philippsburg - JOYclub*. Joyclub. Retrieved September 22, 2023, from <https://www.joyclub.de/event/1267834.white_ib_za_mallorca_fiesta_especial_philippsburg.html>

Faro 8. (2023a). *Der Hurenball***Powered by SweetManiacS&qout; Swinger-Party - 45770 marl - JOYClub*. Joyclub. Retrieved September 22, 2023, from <https://www.joyclub.de/event/1243225.der_hurenball_powered_by_sweetmaniacs_marl.html>

Faro 8. (2023b). *Die 11 Gebote der Sodomiter Open Relationship- & Fetisch-Party - 45770 Marl - JOYclub*. Joyclub. Retrieved October 14, 2023, from <https://www.joyclub.de/event/1259455.die_11_gebote_der_sodomiter_marl.html>

Fetisch Hof Berlin. (2023). *Herbst Hoffest 2023 Open Relationship- & Fetisch-Party - 12055 Berlin - JOYClub*. Joyclub. Retrieved September 22, 2023, from <https://www.joyclub.de/event/1433187.herbst_hoffest_2023_berlin.html>

fetischhaus. (2023). *LET’S PLAY - MASTER AND SERVANT mit DJ BECO!  Open Relationship- & Fetisch-Party - 65201 Wiesbaden - JOYclub*. Joyclub. Retrieved October 14, 2023, from <https://www.joyclub.de/event/1396035.let_s_play_master_and_servant_mit_dj_beco_wiesbade.html>

Feuer und Eis. (2023a). *Couples & Ladies Night - Leuchtbändchen-Alarm Swinger-Party - 76646 Bruchsal - JOYClub*. Joyclub. Retrieved October 14, 2023, from <https://www.joyclub.de/event/1433924.couples_ladies_night_leuchtbaendchen_alarm_bruchsa.html>

Feuer und Eis. (2023b). *Young People Night - No. 189 Swinger-Party - 76646 Bruchsal - JOYClub*. Joyclub. Retrieved September 22, 2023, from <https://www.joyclub.de/event/1426547.young_people_night_no_189_bruchsal.html>

FFF-Extasia. (2023). *Back to the School - College Party Vol. 2 Swinger-Party - 81829 München - JOYclub*. Joyclub. Retrieved September 15, 2023, from <https://www.joyclub.de/event/1426540.back_to_the_school_college_party_vol_2_muenchen.html> Access to the webpage only possible for members with a Joyclub membership. Free memberships qualify for access.

Fun´n more Events. (2023). *Köln´s 1. EROTIK Schlager Party Erotik-Party - 50667 Köln - JOYclub*. Joyclub. Retrieved September 22, 2023, from <https://www.joyclub.de/event/1353784.koeln_s_1_erotik_schlager_party_koeln.html>

GENERATION FUN Events. (2023). *Junge Paare Party - JuPaPa U35 - Bayern Swinger-Party - 91154 Roth - JOYClub*. Joyclub. Retrieved October 14, 2023, from <https://www.joyclub.de/event/1431627.junge_paare_party_jupapa_u35_bayern_roth.html>

Grace & Beauty. (2023). *The Beauty & The Beat @le Coq SummerClosing Erotik-Party - 55286 Wörrstadt - JOYclub*. Joyclub. Retrieved September 22, 2023, from <https://www.joyclub.de/event/1377645.the_beauty_the_beat_le_coq_summerclosing_woerrstad.html>

HotnDirtyEvents / HnD. (2023a). *HND Royale Casino @ Oase Swinger-Party - 63322 Rödermark - JOYClub*. Joyclub. Retrieved October 14, 2023, from <https://www.joyclub.de/event/1362536.hnd_royale_casino_oase_roedermark.html>

HotnDirtyEvents / HnD. (2023b). *VIP Hot n Dirty meets Royal Dance Erotik-Party - 03099 Kolkwitz - JOYclub*. Joyclub. Retrieved September 15, 2023, from <https://www.joyclub.de/event/1319573.vip_hot_n_dirty_meets_royal_dance_kolkwitz.html>

Hotwife events. (2023). *Hotwifeverleih- Bang my Hotwife! Erotik-Party - 91154 Roth - JOYclub*. Joyclub. Retrieved September 22, 2023, from <https://www.joyclub.de/event/1393483.hotwifeverleih_bang_my_hotwife_roth.html>

La Huître. (2023). *Back to the 80’s & 90’s Swinger-Party - 71706 Markgröningen - JOYClub*. Joyclub. Retrieved September 22, 2023, from <https://www.joyclub.de/event/1425726.back_to_the_80_s_90_s_markgroeningen.html>

Life - Club für Paare. (2023a). *LEUCHTBANDPARTY - “DISCOVER THE LIGHT” Swinger-Party - 48712 Gescher - JOYClub*. Joyclub. Retrieved September 22, 2023, from <https://www.joyclub.de/event/1431597.leuchtbandparty_discover_the_light_gescher.html>

Life - Club für Paare. (2023b). *LIFE-WIESN-GAUDI 2023 Swinger-Party - 48712 Gescher - JOYClub*. Joyclub. Retrieved October 14, 2023, from <https://www.joyclub.de/event/1364278.life_wiesn_gaudi_2023_gescher.html>

Life - Club für Paare. (2023c). *ST. PAULI NIGHT von 17.00 Uhr - 04.00 Uhr Swinger-Party - 48712 Gescher - JOYclub*. Joyclub. Retrieved September 15, 2023, from <https://www.joyclub.de/event/1408584.st_pauli_night_von_17_00_uhr_04_00_uhr_gescher.html>

Lifestyle GmbH [Quicky Rhein-Neckar]. (2023). *YoungMen Night Swinger-Party - 69469 Weinheim - JOYClub*. Joyclub. Retrieved October 14, 2023, from <https://www.joyclub.de/event/1425341.youngmen_night_weinheim.html>

Lillith. (2023). *Paare Schnupperabend*WARTELISTE* Swinger-Party - 82178 Puchheim - JOYclub*. Joyclub. Retrieved October 14, 2023, from <https://www.joyclub.de/event/1426018.paare_schnupperabend_warteliste_puchheim.html>

Living Dead Production. (2023). *eXtravaganXa - this is your fetish! Open Relationship- & Fetisch-Party - 22525 Hamburg - JOYclub*. Joyclub. Retrieved October 14, 2023, from <https://www.joyclub.de/event/1425941.extravaganxa_this_is_your_fetish_hamburg.html>

Location One. (2023). *Happy Birthday Chef&lt;unk&gt; (23.09.23) Swinger-Party - 58706 Menden - JOYClub*. Joyclub. Retrieved September 22, 2023, from <https://www.joyclub.de/event/1423433.happy_birthday_chef_23_09_23_menden.html>

Loft54. (2023). *The UNHOLY NIGHT .. Erotik-Party - 76287 Rheinstetten - JOYClub*. Joyclub. Retrieved October 14, 2023, from <https://www.joyclub.de/event/1424760.the_unholy_night_rheinstetten.html>

Malibu Stars. (2023). *Malibu Stars feiert seinen 5. Geburtstag Swinger-Party - 48268 Greven - JOYclub*. Joyclub. Retrieved September 22, 2023, from <https://www.joyclub.de/event/1362830.malibu_stars_feiert_seinen_5_geburtstag_greven.html>

Michas Swingertreff. (2023a). *Nacht der Paare *warteliste* Swinger-Party - 31275 Lehrte - JOYclub*. Joyclub. Retrieved September 22, 2023, from <https://www.joyclub.de/event/1434340.nacht_der_paare_lehrte.html>

Michas Swingertreff. (2023b). *Nacht der Paare *Warteliste* Swinger-Party - 31275 Lehrte - JOYclub*. Joyclub. Retrieved October 14, 2023, from <https://www.joyclub.de/event/1434333.nacht_der_paare_warteliste_lehrte.html>

O-Events. (2023). *Die Nacht der O in München Open Relationship- & Fetisch-Party - 81245 München - JOYclub*. Joyclub. Retrieved October 14, 2023, from <https://www.joyclub.de/event/1381840.die_nacht_der_o_in_muenchen_muenchen.html>

Pärchenclubhotel Schiedel. (2023a). *SCHIED’LER OKTOBERFEST’2023+KÄpt’ Pinselbube Swinger-Party - 01917 Kamenz - JOYClub*. Joyclub. Retrieved October 14, 2023, from <https://www.joyclub.de/event/1435023.schied_ler_oktoberfest_2023_kaept_pinselbube_kamen.html>

Pärchenclubhotel Schiedel. (2023b). *Uniform Party 2.0 Swinger-Party - 01917 Kamenz - JOYClub*. Joyclub. Retrieved September 15, 2023, from <https://www.joyclub.de/event/1420006.uniform_party_2_0_kamenz.html>

Port of Senses. (2023). *der FemDomBall, the night of power Open Relationship- & Fetisch-Party - 76189 Karlsruhe - JOYclub*. Joyclub. Retrieved September 15, 2023, from <https://www.joyclub.de/event/1371089.der_femdomball_the_night_of_power_karlsruhe.html>

RagaZza.Club. (2023). *CouplesNight-Die Paare Party Swinger-Party - 66780 Rehlingen-Siersburg - JOYClub*. Joyclub. Retrieved October 14, 2023, from <https://www.joyclub.de/event/1451984.couplesnight_die_paare_party_rehlingen_siersburg.html>

REAL YOUNGSTER. (2023). *“YOUNGSTER COUPLE & BI-GIRLS NIGHT” Swinger-Party - 86836 Untermeitingen - JOYClub*. Joyclub. Retrieved September 22, 2023, from <https://www.joyclub.de/event/1422821.youngster_couple_bi_girls_night_mit_vk_untermeitin.html>

Scandal Events. (2023). *SCANDAL BIZARRE Open Relationship- & Fetisch-Party - 76316 Malsch - JOYClub*. Joyclub. Retrieved September 22, 2023, from <https://www.joyclub.de/event/1413973.scandal_bizarre_malsch.html>

Schloss Milkersdorf. (2023a). *EROTISCHE SCHLOSSNACHT - schwarz oder weiß Erotik-Party - 03099 Kolkwitz - JOYclub*. Joyclub. Retrieved September 22, 2023, from <https://www.joyclub.de/event/1359315.erotische_schlossnacht_schwarz_oder_weiss_kolkwitz.html>

Schloss Milkersdorf. (2023b). *SCHLOSSNACHT DELUXE Erotik-Party - 03099 Kolkwitz - JOYClub*. Joyclub. Retrieved October 14, 2023, from <https://www.joyclub.de/event/1359319.schlossnacht_deluxe_kolkwitz.html>

Secret Lions. (2023). *SCHAUMPARTY - die Megaparty Erotik-Party - 49811 Lingen - JOYClub*. Joyclub. Retrieved September 22, 2023, from <https://www.joyclub.de/event/1426302.schaumparty_die_megaparty_lingen.html>

SF Lounge. (2023a). *NACHT DER PAARE • COLOR EDITION Swinger-Party - 41564 Kaarst - JOYClub*. Joyclub. Retrieved September 22, 2023, from <https://www.joyclub.de/event/1420952.nacht_der_paare_color_edition_kaarst.html>

SF Lounge. (2023b). *NACHT DER PAARE Swinger-Party - 41564 Kaarst - JOYClub*. Joyclub. Retrieved October 14, 2023, from <https://www.joyclub.de/event/1423269.nacht_der_paare_kaarst.html>

Sin City Events. (2023). *[THE FUSION-COUPLE EDITION] SUMMER Erotik-Party - 63322 Rödermark - JOYClub*. Joyclub. Retrieved September 15, 2023, from <https://www.joyclub.de/event/1394241.the_fusion_couple_edition_summer_roedermark.html>

SNA Event Concept. (2023). *SNA - Alf Leila wa Leila Swinger-Party - 53881 Euskirchen - JOYclub*. Joyclub. Retrieved September 22, 2023, from <https://www.joyclub.de/event/1387392.sna_alf_leila_wa_leila_euskirchen.html>

Steinenhaus. (2023a). *Black-ToyBoy@Steinenhaus, Sa., 16.09 19 uhr Swinger-Party - 45527 Hattingen - JOYClub*. Joyclub. Retrieved September 15, 2023, from <https://www.joyclub.de/event/1416105.black_toyboy_steinenhaus_sa_16_09_19_uhr_hattingen.html>

Steinenhaus. (2023b). *KAIs Aktiv-Swinger-Party – PAARE-Edition Swinger-Party - 45527 Hattingen - JOYClub*. Joyclub. Retrieved October 14, 2023, from <https://www.joyclub.de/event/1242125.kais_aktiv_swinger_party_paare_edition_hattingen.html>

Steinenhaus. (2023c). *MOLLY_LOVER GOES STEINENHAUS  all inklusive Swinger-Party - 45527 Hattingen - JOYclub*. Joyclub. Retrieved September 22, 2023, from <https://www.joyclub.de/event/1241316.molly_lover_goes_steinenhaus_all_inklusive_hatting.html>

Studio 52. (2023). *Endlich Wochenende Dance-Party - 63452 Hanau - JOYClub*. Joyclub. Retrieved October 14, 2023, from <https://www.joyclub.de/event/1432800.endlich_wochenende_hanau.html>

SunMoon. (2023a). *Black in White “Interracial Night” Swinger-Party - 74343 Sachsenheim - JOYClub*. Joyclub. Retrieved October 14, 2023, from <https://www.joyclub.de/event/1425135.black_in_white_interracial_night_sachsenheim.html>

SunMoon. (2023b). *Gangbang , wild, versaut & extrem unartig Gangbang - 74343 Sachsenheim - JOYclub*. Joyclub. Retrieved September 22, 2023, from <https://www.joyclub.de/event/1430923.gangbang_wild_versaut_extrem_unartig_sachsenheim.html>

Swingerclub Harz-Heide. (2023). *Mollyparty ! Warteliste. Swinger-Party - 38159 Vechelde - JOYClub*. Joyclub. Retrieved October 14, 2023, from <https://www.joyclub.de/event/1454581.mollyparty_warteliste_vechelde.html>

Swingerpark Extra-3. (2023). *OKTOBERFEST 2023  Swinger-Party - 31542 Bad Nenndorf - JOYClub*. Joyclub. Retrieved October 14, 2023, from <https://www.joyclub.de/event/1427563.oktoberfest_2023_bad_nenndorf.html>

sXXXtasy. (2023). *C U R V y - l O v E r Swinger-Party - 53881 Euskirchen - JOYclub*. Joyclub. Retrieved October 14, 2023, from <https://www.joyclub.de/event/1338291.c_u_r_v_y_l_o_v_e_r_euskirchen.html>

Tabou Events. (2023). *República del Hedonismo - Live Night Dance-Party - 90427 Nürnberg - JOYclub*. Joyclub. Retrieved October 14, 2023, from <https://www.joyclub.de/event/1336793.republica_del_hedonismo_live_night_nuernberg.html>

Tempeloase. (2023a). *Bändchen Party Tempeloase mit DJ Zyko ! Swinger-Party - 14979 Großbeeren - JOYclub*. Joyclub. Retrieved October 14, 2023, from <https://www.joyclub.de/event/1432999.baendchen_party_tempeloase_mit_dj_zyko_grossbeeren.html>

Tempeloase. (2023b). *BERLIN Young and sexy couple ladies PAARE Swinger-Party - 14979 Großbeeren - JOYclub*. Joyclub. Retrieved September 15, 2023, from <https://www.joyclub.de/event/1340406.berlin_young_and_sexy_couple_ladies_paare_grossbee.html>

Tempeloase. (2023c). *Bye bye Summer Preisknaller !!! Swinger-Party - 14979 Großbeeren - JOYclub*. Joyclub. Retrieved September 22, 2023, from <https://www.joyclub.de/event/1432825.bye_bye_summer_preisknaller_grossbeeren.html>

Villa Party. (2023). *Villa Party am 23. September Erotik-Party - 25462 Rellingen - JOYclub*. Joyclub. Retrieved September 22, 2023, from <https://www.joyclub.de/event/1411711.villa_party_am_23_september_rellingen.html>

WAALberg. (2023a). *Die SCHULMÄDCHEN PARTY (Rheinland-Pfalz) Swinger-Party - 56075 Koblenz - JOYClub*. Joyclub. Retrieved October 14, 2023, from <https://www.joyclub.de/event/1378896.die_schulmaedchen_party_rheinland_pfalz_koblenz.html>

WAALberg. (2023b). *„Finest Moments - Club Night“ - St. Tropez Erotik-Party - 86444 Affing - JOYClub*. Joyclub. Retrieved October 14, 2023, from <https://www.joyclub.de/event/1408045.finest_moments_club_night_st_tropez_affing.html>

Wilder Süden. (2023). *EROTISCHE CASINO NIGHT! 2 Clubs - 1 party Erotik-Party - 86444 Affing - JOYClub*. Joyclub. Retrieved September 22, 2023, from <https://www.joyclub.de/event/1396013.erotische_casino_night_2_clubs_1_party_affing.html>

XtraJOY Augsburg. (2023). *XtraJOY “Pärchen FREI” Erotik-Party - 86154 Augsburg - JOYClub*. Joyclub. Retrieved October 14, 2023, from <https://www.joyclub.de/event/1458692.xtrajoy_paerchen_frei_augsburg.html>

YourNITELIFE. (2023a). *Leipzig Young & Sexy #YAS #LEIPZIG Swinger-Party - 04435 schkeuditz - JOYclub*. Joyclub. Retrieved October 14, 2023, from <https://www.joyclub.de/event/1418850.leipzig_young_sexy_yas_leipzig_schkeuditz.html>

YourNITELIFE. (2023b). *Munich Young Exclusive PAARE EDITION #YNL Swinger-Party - 81829 München - JOYClub*. Joyclub. Retrieved September 22, 2023, from <https://www.joyclub.de/event/1418847.munich_young_exclusive_paare_edition_ynl_muenchen.html>

YourNITELIFE. (2023c). *Munich Young Exclusive PAARE EDITION #YNL Swinger-Party - 81829 München - JOYClub*. Joyclub. Retrieved October 14, 2023, from <https://www.joyclub.de/event/1418852.munich_young_exclusive_paare_edition_ynl_muenchen.html>

# E. Figure Related to Study 1

## Figure S3: Location Key – Swinger Density

*Identified swingers per 100,000 inhabitants of German counties referenced in the study.*

County boundary data © GeoBasis-DE / BKG (2023) (data changed).


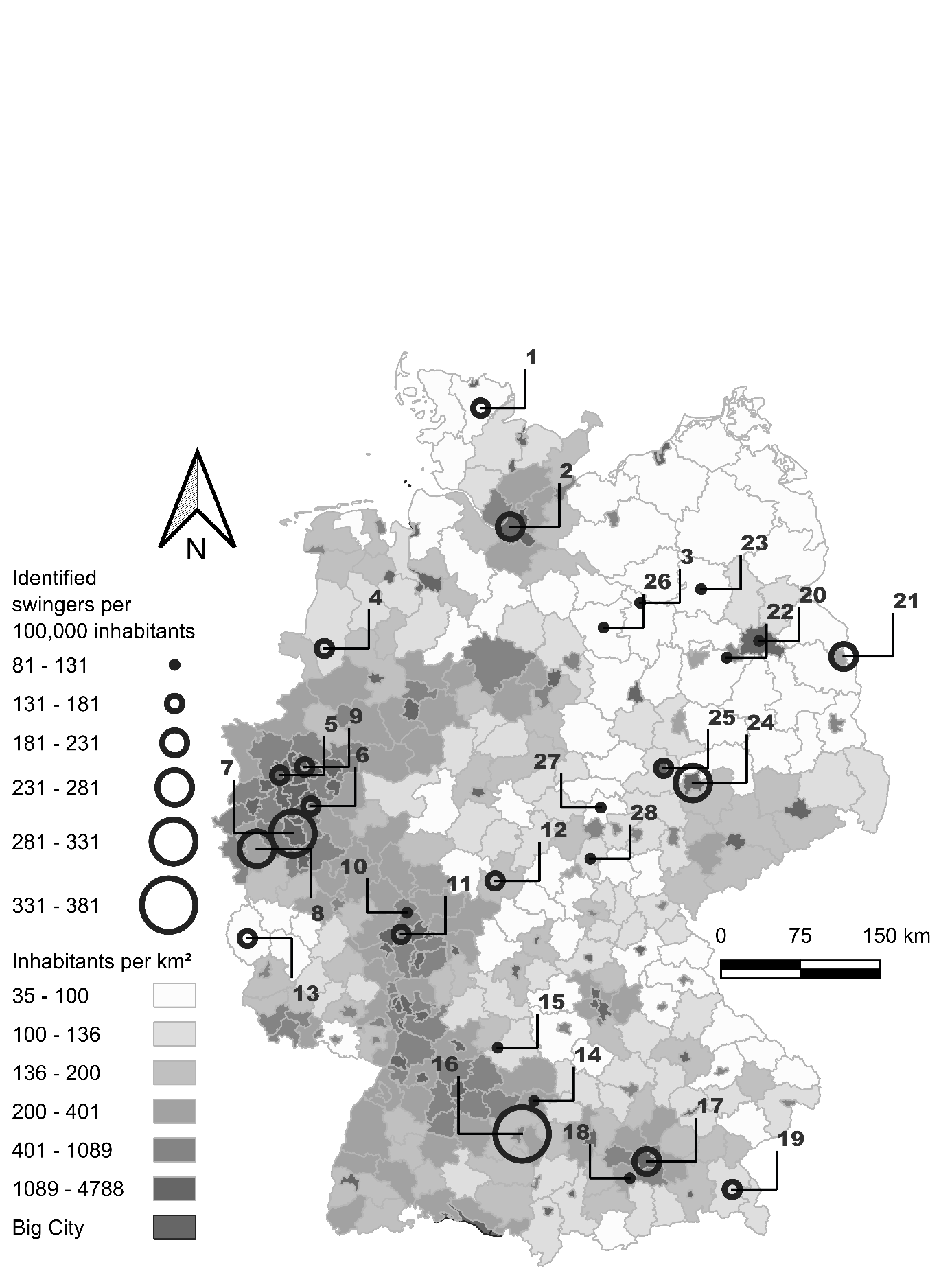


## Key to Figure S3

### Lines

The lines show the boundaries of counties.

### Table S16: Key List

*Key to the Numbers Used in Figure S3*

| Number | Official name of county |
| --- | --- |
| 1 | Schleswig-Flensburg, Landkreis |
| 2 | Hamburg |
| 3 | Lüchow-Dannenberg, Landkreis |
| 4 | Grafschaft Bentheim, Landkreis |
| 5 | Duisburg, kreisfreie Stadt |
| 6 | Remscheid, kreisfreie Stadt |
| 7 | Köln, kreisfreie Stadt [Cologne] |
| 8 | Düren, Kreis |
| 9 | Gelsenkirchen, kreisfreie Stadt |
| 10 | Hochtaunuskreis |
| 11 | Main-Taunus-Kreis |
| 12 | Fulda, Landkreis |
| 13 | Eifelkreis Bitburg-Prüm |
| 14 | Heidenheim, Landkreis |
| 15 | Ostalbkreis, Landkreis |
| 16 | Ulm, Stadtkreis |
| 17 | München, Landeshauptstadt and Landkreis [Munich] |
| 18 | Starnberg, Landkreis |
| 19 | Traunstein, Landkreis |
| 20 | Berlin |
| 21 | Frankfurt (Oder), kreisfreie Stadt |
| 22 | Potsdam-Mittelmark, Landkreis |
| 23 | Prignitz, Landkreis |
| 24 | Leipzig Stadt and Landkreis |
| 25 | Halle (Saale), kreisfreie Stadt |
| 26 | Altmarkkreis Salzwedel |
| 27 | Sömmerda, Kreis |
| 28 | Ilm-Kreis |

## License Related to Figure S3

Land border data: Data licence Germany – attribution – Version 2.0 (FITKO, n.d.).

## References Related to the Data Source and License

BKG (2023). *Verwaltungsgebiete 1:5 000 000, Stand 31.12.* [Administrative areas 1:5,000,000, as of December 31] (VG5000 31.12.) [Data Set]. Bundesamt für Kartographie und Geodäsie. <https://gdz.bkg.bund.de/index.php/default/verwaltungsgebiete-1-5-000-000-stand-31-12-vg5000-12-31.html>

FITKO (n.d.). *DL-DE->BY-2.0*. Govdata. <http://www.govdata.de/dl-de/by-2-0>

# F. Figure Related to Study 2

## Figure S4: Location Key – Venue Locations

*Locations of event venues in Germany referenced in the study.*

State boundary data © GeoBasis-DE / BKG (2023) (data changed).


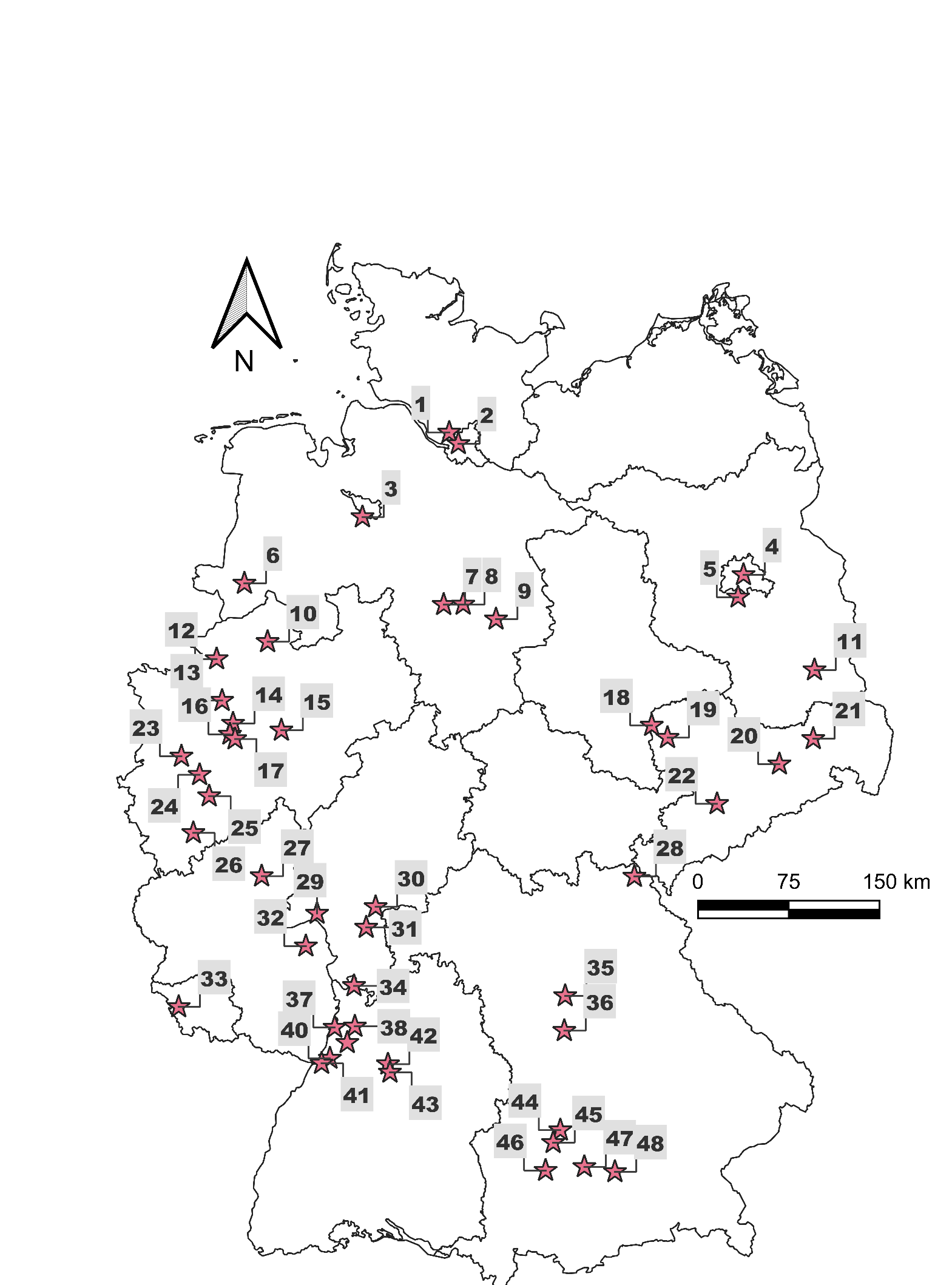


## Key to Figure S4

### Lines and Stars

The lines represent land borders of the German Länder (states). The stars show the location of cities and towns of event venues referenced in the study.

### Table S17: Key List

*Key to the Numbers Used in Figure S4*

| Number | City or town |
| --- | --- |
| 1 | Rellingen |
| 2 | Hamburg |
| 3 | Stuhr |
| 4 | Berlin |
| 5 | Großbeeren |
| 6 | Lingen |
| 7 | Hannover |
| 8 | Lehrte |
| 9 | Vechelde |
| 10 | Greven |
| 11 | Kolkwitz |
| 12 | Gescher |
| 13 | Marl |
| 14 | Bochum |
| 15 | Menden |
| 16 | Hattingen |
| 17 | Sprockhövel |
| 18 | Wiedemar |
| 19 | Leipzig |
| 20 | Radebeul |
| 21 | Kamenz |
| 22 | Chemnitz |
| 23 | Kaarst |
| 24 | Dormagen |
| 25 | Cologne [Köln] |
| 26 | Euskirchen |
| 27 | Koblenz |
| 28 | Hof |
| 29 | Wiesbaden |
| 30 | Hanau |
| 31 | Rödermark |
| 32 | Wörrstadt |
| 33 | Rehlingen-Siersburg |
| 34 | Weinheim |
| 35 | Nürnberg |
| 36 | Roth |
| 37 | Philippsburg |
| 38 | Malsch |
| 39 | Bruchsal |
| 40 | Karlsruhe |
| 41 | Rheinstetten |
| 42 | Sachsenheim |
| 43 | Markgröningen |
| 44 | Affing |
| 45 | Augsburg |
| 46 | Untermeitingen |
| 47 | Fürstenfeldbruck |
| 48 | Munich [München] |

## License to Figure S4

Land border data: Data licence Germany – attribution – Version 2.0 (FITKO, n.d.).

## References Related to the Data Source and License

BKG (2023). *Verwaltungsgebiete 1:5 000 000, Stand 31.12.* [Administrative areas 1:5,000,000, as of December 31] (VG5000 31.12.) [Data Set]. Bundesamt für Kartographie und Geodäsie. <https://gdz.bkg.bund.de/index.php/default/verwaltungsgebiete-1-5-000-000-stand-31-12-vg5000-12-31.html>

FITKO (n.d.). *DL-DE->BY-2.0*. Govdata. <http://www.govdata.de/dl-de/by-2-0>

# G. Legal and Ethical Assessment

## Legality

### Database Protection Laws

Applicable database protection laws are those of the Federal Republic of Germany. The author as well as the operator of the website Joyclub ([https://www.joyclub.de](https://www.joyclub.de/)) have their residence or legal seat, respectively, in Germany (Joyclub, n.d.-a).

German database protection laws expressly regulate data harvesting from databases that are accessible for researchers.

- Written confirmation from F & P GmbH, Selbitz, the operators of Joyclub, has been obtained confirming the compliance of the use of the data for this study with their terms and conditions.
- According to Section 87b of the German Copyright Act (Federal Republic of Germany [Federal Ministry of Justice] & Federal Office of Justice, n.d.-a), which regulates database protection, "the producer of a database has the exclusive right to reproduce and distribute the database as a whole or a qualitatively or quantitatively substantial part of the database and to make it available to the public." According to Section 87a of the German Copyright Act, a *database* is any data "arranged in a systematic or methodical way and individually accessible by electronic or other means and whose obtaining, verification or presentation requires a substantial qualitative or quantitative investment."
- According to Section 60d of the German Copyright Act, text and data mining for purposes of non-commercial scientific research are expressly permitted, thus limiting the exclusive right of the database producer (Mandon, 2018). According to Section 60g(1) of the Copyright Act, a the database producer may not rely on conflicting contractual agreements, such as i.e. contained in its general terms and conditions.
- The contents of the databases collected in this way may also be transmitted to third parties for the purpose of checking the quality of scientific research, i.e., in particular for peer reviews (Section 60d(4)2 of the German Copyright Act). This applies only to the corpus of data, not to the source material, which may not be redistributed (Brettschneider, 2021); upon permitted sharing of the corpus, the sources must be stated (Mandon, 2018).
- The database right is not a right over the information stored in the database (Duisberg, 2017). Database protection exists independent of the content of the database (Kleinkopf, 2022, p. 162). Therefore, analysis in the context of text and data mining as such is permitted by copyright law even without a separate legal norm that would allow it (Kleinkopf, 2022, p. 170). Consequently, if database data are processed, in particular aggregated, and thus the specific arrangement of the data in the original database is not included in the new work thus created, it is not identical with the protected work. The producer of the original database then cannot claim any rights in the resulting new work.
- For this study, the consequences are as follows:
  - The requirements for the legality of the data collection, as provided for in the database protection laws, were fulfilled. The data for this study were collected with a properly set up Joyclub profile, which allows access to the respective data. Eventual contravening terms and conditions were not identified but would anyway be legally irrelevant under German law.
  - The statutory requirements for the legality of the production of a structured corpus from the data collected were fulfilled.
  - The corpus itself may only be transmitted to third parties for the purpose of checking the quality of scientific research, i.e., in particular in peer reviews.
  - The data in the original database do not enjoy any legal protection by themselves. The same applies to data derived from them. The database protection relates to the combination of, as the first element, the arrangement, and as the second element, the content of the database, and not to any of these two elements without the respective other element. Therefore, it does not relate to the data contained in the database alone, and not to any data deriving from them. Because the aggregated data (e.g., medians, standard deviations) do not reflect the structure of the original database, and are newly generated and not the original data, they are not subject to the database rights which rest with the original database and can thus be shared.
  - In addition, because they do not form a product of any human creativity, the original data retrieved are not subject to any copyright. This is even more true for derivative data.
  - To avoid misunderstandings, it has to be emphasized that the collection of the metadata of the events had not been based on structured data (and, thus, a database), but retrieved from individual entries on the webpage produced by the organizers of the events.

### Data Protection Laws

- Data protection law, in particular the European Union General Data Protection Regulation (GDPR) (European Union: Publications Office of the EU, 2015), which is directly applicable law in Germany, refers only to personal data. According to Article 4 of the GDPR, *personal data* are data relating to an identified or identifiable natural person (*data subject*). A data subject is a natural person who can be identified, directly or indirectly, in particular by reference to an identifier such as a name, an identification number, location data, an online identifier or to one or more factors specific to the physical, physiological, genetic, mental, economic, cultural, or social identity of that natural person. As long as data are collected that allow individualization, they are personal data. In that case, the processing of the data is subject to the GDPR.
- Profile data in specific the form in which they can be retrieved in Joyclub, as the result of a member search or as registration data for an event, are therefore personal data, if a profile name is provided. A profile name chosen by individuals serves the purpose to identify them pseudonymously or openly. Therefore, it clearly serves the purpose of an identifier within the meaning of Article 4 of the GDPR.
- For the assessment of the legality of sharing the corpus under the GDPR, it is relevant if individuals can be re-identified from simple age and county data, as they are contained in the corpus. The corpus does not contain further identifiers, so the data would not have the status of personal data if they would not allow re-identification. Without that status, any restrictions imposed in the GDPR would not apply. In the other case, any use of the data would be subject to the limitations provided for by the GDPR.
- The county with the smallest number of inhabitants processed in the analysis was the city of Frankfurt (Oder) in Brandenburg, Germany. Frankfurt (Oder) has a population of ca. 54,000 inhabitants.
- The admitted visitors could visit the respective events from whole of Germany, with more than 83 million inhabitants, or from abroad.
- Sweeney (2000) has shown that it is nearly impossible to re-identify persons in populations of that size if only the age, in years, and the gender are known. Rocher et al. (2019) have demonstrated that the possibility of the re-identification of individuals rises with the increasing number of attributes known but have confirmed the result that Sweeney had presented before relating to cases where only two or three attributes are known, and where the population of the relevant region is as high as 50,000. Therefore, the risk of re-identifying persons from the data collected for this study appears to be low. With respect to visitors of events, a re-identification of persons only on the basis of the age and gender of the visitor appears highly unlikely, because visitors travel longer distances to events, as the results have shown. In these cases, the population to which the individuals belong is too large for identification.
- To comply with highest ethical standards, data in the corpus, which contain data on the age, the gender, and the county of residence of persons are not published as open data. Also, in line with the legal restrictions mandated with a view to database protection, see the Database Protection Laws section, the corpus will only be made accessible individually for the sole purpose of checking the quality of scientific research, i.e., in particular in peer reviews.
- In the light of the above, the GDPR rules might become relevant for this research as follows.
  - In a very short transitional phase, data from Joyclub profiles, without any profile pictures, were retrieved and stored as source data. These data were collected in the form of the textual data available to all members of Joyclub ([https://www.joyclub.de](https://www.joyclub.de/)), containing the profile data shown on the results pages as search results and of event registrations, respectively. In detail, they contained the profile pseudonym (nickname), any alternative texts to the profile pictures provided by the users, and shown on the results pages, each along with the age, gender, and residence county data. The detailed profile pages of members were not accessed.
  - These source data were immediately converted into a streamlined dataset, using the method described in the Scripts folder in the data package. That streamlined data set only contained information on the age or ages, county, and gender or genders; the plural applies to couple profiles.
  - The collected source data are *particularly protected data* within the meaning of Article 9 of the General Data Protection Regulation. Within the context of Joyclub, the data concern sex lives of natural persons.
  - However, these data are exempt from the restrictions which are generally imposed on the use of particularly protected data by the General Data Protection Regulation, because they were each manifestly made public by the respective data subjects within the meaning of Article 9(2)(e) of the Regulation.
  - The European Court of Justice interpreted the exemption contained in Article 9(2)(e) in its decision *Meta Platforms and Others v Bundeskartellamt* of July 4, 2023 (European Court of Justice, 2023) in some detail. According to the court, the decisive test for the application of the exemption rule is whether the data have been made available to an unlimited number of other individuals by the data controller on the basis of an informed decision. "Where, on the basis of individual settings selected with full knowledge of the facts, those users have clearly made the choice to have the data made accessible to an unlimited number of persons" (European Court of Justice, 2023, para. 82), the conditions for the application of the exemption are fulfilled. "If no such individual settings are available, it must be held [...] that, where users voluntarily enter information into a website [...], they must, in order to be deemed to have manifestly made that data public, have explicitly consented, on the basis of express information provided by that site or app prior to any such entering or clicking or tapping, to the data being viewed by any person having access to that site." (European Court of Justice, 2023, para. 83).
  - This applies to the profiles themselves, because they are created precisely for the purpose of serving as a presentation of the own person and preferences. The presentation of the profile content to a general audience is exactly the purpose of the profile creation. If the persons creating profiles do not want the data published, they either would not enter them, or could subsequently delete their profiles.
  - When registering for an event, users have the option to register anonymously, as is evident from every event page on Joyclub (https://www.joyclub.de). They will be informed of this option before registering and that anonymous registration prevents the possibility of newly made acquaintances contacting them afterwards. In the case of anonymous registration, the profile of the person registering is disclosed to the organizer. Only other guests see the profile only with an avatar, the writing "Anonymous" instead of the profile name and the age and gender of the registered visitor. The anonymously registered and the non-anonymous visitors are displayed on the page of the event after activation by the organizer.
  - Due to the possibility to register anonymously for events, and because it is immediately obvious when calling up event pages that registrants will be listed on the event page after confirmation, non-anonymous event registrations are thus manifestly made public within the sense of the interpretation of the European Court of Justice. They are therefore subject to the exception in Article 9(2)(e).
  - As Joyclub states a number of registered members on several of its webpages in the website, and since that number exceeds five million, and because, from their own registration, users know that everyone has the possibility to register to the website for free, it is also clear to users that they do not communicate to a small number of persons, but to a potentially unlimited audience.
  - In addition to the above, the exception in Article 9(2)(j) of the General Data Protection Regulation, which grants the Member States legislative competence to regulate data processing for scientific research purposes, applies. In Section 27(1) of the German Federal Data Protection Act (Federal Republic of Germany [Federal Ministry of Justice] & Federal Office of Justice, n.d.-a), the German federal legislature has provided a general provision for the use of particularly research purposes on condition that the interest of the processor clearly outweighs that of the data subject and that appropriate safeguards have been put in place. Because of the immediate anonymization, against the background that the Data Subjects themselves have made the data accessible to an unlimited number of persons, because the data are pseudonymous at the onset, as users mostly do not use real names as profile names, and against the background that the corpus would only be transferred for the limited purpose of the assessment of the quality of science, the interest of the processor clearly outweighs the interest of the data subjects. The immediate anonymization of the data provided for in Section 27 (3) of the Federal Data Protection Act has been carried out.
- In summary, the data retrieved are immediately anonymized and unlikely to allow re-identification of the data subjects, and likely not be considered as data which are related to an identifiable data subject, which excludes them from the scope of the European data protection regulations. Even if the data should be considered person-related, an exemption would apply to them, because the data subjects knowingly and manifestly made the data public. In addition, German federal legislation applies which allows the processing of data for scientific purposes, as the interest in processing clearly outweighs the interests of the data subjects for the reasons stated.

### Issue of the Notification of Data Subjects

Subsequent notification of the data subjects about the processing of their data is neither legally mandated nor reasonable under Section 27(2) of the German Federal Data Protection Act. In the design of this research project, priority had been given to immediate anonymization. A notification would not have added value for the data subjects. On the contrary, identifying information would have to be retained beyond the short period of time before the built-up of the corpus. In addition, it would not have been logistically feasible to notify the data subjects. As the only possibility for such communication would have been internal Joyclub messages, the manual creation of messages within Joyclub to the owners of the pseudonymous profiles would have been necessary.

## Ethical Considerations beyond legal questions

The comprehensive considerations that the European and German legislators have made on individual issues of data use in terms of copyright and data protection law are obviously strongly guided by ethical considerations. The resulting laws form a legally binding framework for research ethics.

### Possibility of Group-Based Discrimination

An ethical consideration that goes beyond purely legal considerations relates to address group-based discrimination against the individuals under investigation. Haywood (2022) had discussed that issue in connection with a study which involved harvesting swinger data in the United Kingdom. However, the situations of swingers in the United Kingdom on one hand and of swingers in Germany on the other hand are different. In Germany, swingers do not have to fear group-related collective discrimination or other disadvantages, as explained in further detail here below.

### Legality of Swinging in Germany

- From a legal point of view, swinging is allowed in Germany without any restrictions which are not contained in general laws, e.g., regarding consent and a minimum age for sexual acts. Swinger clubs are considered to be restaurants and require a permit if alcohol is served there.
- In 2002, the highest German court responsible for administrative law (Bundesverwaltungsgericht, 2002) ruled on a complaint against the refusal of a restaurant permit for the operation of a swingers’ club. The court had to decide whether the competent authority must refuse the requested license. According to the laws, the respective authorities must not permit the operation of a restaurant if facts justify the assumption that the applicant holder does not possess the necessary reliability for the business operation, in particular encourages immorality. The court held that operating a swingers’ club does not constitute immorality within the meaning of the statute. According to the court, the concept of immorality must reflect the right of the individual to the free development of his or her personality, which also includes the right to freedom in the organization of the intimate sphere. The court explained that individuals enjoy a right to self-determination as to the form in which they organize their sexual lives, insofar as this does not violate the constitutional order of values, normative requirements, or the rights of others. The court argued that the purpose of the immorality restriction contained in the restaurant law was not to promote morality as such or to educate to it. The generation of income from sex-related behavior of others could generally not be considered immoral, according to the court, since the legislature had also made it possible, for example, to create good working conditions for prostitutes in luxury brothels and sauna clubs. Finally, the human dignity of the visitors had not been seen violated by the court, because no objective role was imposed on the visitors that could be considered degrading, but they freely decide in which way they participate in the event.
- In the past 20 years, there were no incidents reported where authorities or courts have seen any reason to intervene against swingers’ clubs, including on the basis of complaints from neighbors. In a recent case, the city administration of Koblenz, Rhineland-Palatinate, had issued a permit which expressly stated the operation of a swinger club as the type of operation of a restaurant. This happened in response to a complaint from a neighbor who had noticed that the earlier license of the already existing swinger club did not contain the appropriate mention. As a consequence to the reaction of the city administration, the neighbor filed a lawsuit that was directed against the updated permit and that, in addition, was aimed at obtaining a court order obliging the administration to intervene against the operation of the club. The neighbor did not state immorality, but purported nuisance by traffic and noise resulting from the operation as the reason for the complaint. The lawsuit was unsuccessful before the Higher Administrative Court (Rheinland-Pfalz, 2021), as it was in the subsequent final instance (Bundesverwaltungsgericht, 2022).
- As emphasized by Stühler (2006), municipalities are not allowed to define and implement a swingers club policy in the context of urban land use planning. They lack any own regulatory competence on these matters.

### No Danger of Job Loss

Individual guests of swingers’ clubs in Germany do not have to fear any legal disadvantages due to their activity. Employment contracts may not be terminated, and civil servants or soldiers must not be dismissed because of extramarital sexual relations (Mayer, 2013).

### No Disadvantages in Family Law

Disadvantages of a family law nature, such as the assumption of an unwritten public order as at least previously in Spain (Navarro, 2017), have not been reported in Germany.

### No Negative Press Coverage

Negative coverage of swingers’ clubs in the German public sphere cannot be identified. In contrast to reports on British media reactions to sex in public places (Hennelly, 2010), the German tabloid press portrays the clubs in a positive light, highlighting, for example, how visits increase guests' self-confidence (Witte, 2023) or how an older gentleman took over the management of a club in order to remain sprightly (Selig, 2023). The national press reported about Joyclub in a neutral manner, presenting the website to a larger audience (Ponath, 2022; Süddeutsche Zeitung, 2012).

### Clubs not Operating in Secrecy

Because of the neutral to friendly treatment of the topic in the media as well as by the administrative and judicial practice, a clandestine appearance of the scene is not required. The secrecy of the clubs described by Haywood (2022) with regard to the United Kingdom is not prevalent in Germany.

- In contrast, in the city of Hattingen, as can also be seen from the bus network map for the city (Verkehrsgesellschaft Ennepe-Ruhr mbH & Verkehrsverbund Rhein-Ruhr AöR, 2020), a bus stop is named after the "Steinenhaus" swingers club next to which it is located.
- Even though the majority of the German population may not be inclined towards CNM relationships and does not engage in swinger activities, the topic has not caused any commotion for decades and has not led to attempts at containment by politicians or authorities. Individual court cases are rather due to neighbor disputes, which thematically dealt with noise and comparable problems (Rheinland-Pfalz, 2021).

### Joyclub is not a Clandestine Niche Website

The portal Joyclub itself is not a clandestine operation, as well.

- The platform originated from a chat, which then turned into a forum for discussing erotic topics and was transformed into the Joyclub platform in 2005 (Ponath, 2022).
- According to its own information on the member search page (Joyclub, n.d.-b), accessed October 15, 2023, it had 5,247,900 members at that time. The alleged membership figure could not be independently verified, nor does it appear in the audited financial statement documents of the operating company F & P GmbH, Selbitz (Ackermann & Noack, 2024).
- The platform, which was already described as a market leader in the national press in 2012 (Süddeutsche Zeitung, 2012), employed four managing directors and 174 employees including mini-jobbers in 2022. In the fiscal year 2022, it was able to increase sales by 26.9% compared to the previous year. Against the background of an equity ratio of 80.5%, the coverage of all accruals liabilities by liquid funds and a return after taxes in relation to total output of 29.1%, the management described the situation in the last published annual financial statements (Ackermann & Noack, 2024) as very good and the sales and earnings development as pleasing.

### Conclusion

For this reason, I decided, in the interest of the transparency of the research, to take a different approach than Haywood (2022), and to expressly mention the portal Joyclub as well as the venues where the different events which were researched took place.

## References Related to the Legal and Ethical Statement

Ackermann, I., & Noack, F. (2024). *F & P GmbH Selbitz: Jahresabschluss zum Geschäftsjahr vom 01.01.2022 bis zum 31.12.2022* [F & P GmbH Selbitz: Annual financial statements for the financial year from January 1, 2022 to December 31, 2022]. Unternehmensregister. <https://www.unternehmensregister.de/ureg/search1.1.html>

Brettschneider, P. (2021). Text und Data-Mining – juristische Fallstricke und bibliothekarische Handlungsfelder [Text and data mining—legal pitfalls and library action areas]. *Bibliotheksdienst, 55*(2), 104–126. <https://doi.org/10.1515/bd-2021-0020>

Bundesrepublik Deutschland [Bundesministerium der Justiz] & Bundesamt für Justiz (Eds.). (n.d.-a). *BDSG - Bundesdatenschutzgesetz* [Federal Data Protection Act]. [gesetze-im-internet.de](http://gesetze-im-internet.de/). Retrieved October 15, 2023, from <https://www.gesetze-im-internet.de/bdsg_2018/BJNR209710017.html>

Bundesrepublik Deutschland [Bundesministerium der Justiz] & Bundesamt für Justiz (Eds.). (n.d.-b). *Act on Copyright and Related Rights (Urheberrechtsgesetz – UrhG)* (U. Reusch, Trans.). [gesetze-im-internet.de](http://gesetze-im-internet.de/). Retrieved October 27, 2023, from <https://www.gesetze-im-internet.de/englisch_urhg/englisch_urhg.html>

Bundesverwaltungsgericht. (2002). BVerwG 6 C 16.02, Judgment of November 6, 2022, ECLI:DE:BVerwG:2002:061102U6C16.02.0. <https://www.bverwg.de/de/061102U6C16.02.0>

Duisberg, A. (2017). „Datenhoheit und Recht des Datenbankherstellers“ – Recht am Einzeldatum vs. Rechte an Datensammlungen ["Data sovereignty and the right of the database producer"—Right to the individual data vs. rights to data collections] [PDF]. In FZI Forschungszentrum Informatik [Smart-Data-Begleitforschung] (Ed.), *Daten als Wirtschaftsgut: Europäische Datenökonomie oder Rechte an Daten?* (pp. 16–27). <https://www.digitale-technologien.de/DT/Redaktion/DE/Downloads/Publikation/2017-11-22_smartdata_daten_wirtschaftsgut.pdf?__blob=publicationFile&v=3>

European Court of Justice. (2023, July 4). Judgment of July 4, 2023, Meta Platforms and Others v Bundeskartellamt, C-252/21, ECLI:EU:C:2023:537 [Press release]. <https://curia.europa.eu/juris/document/document.jsf?text=&docid=275125&pageIndex=0&doclang=EN&mode=req&dir=&occ=first&part=1&cid=1628197>

European Union: Publications Office of the EU (Ed.). (2015). REGULATION (EU) 2016/679 OF THE EUROPEAN PARLIAMENT AND OF THE COUNCIL of 27 April 2016 on the protection of natural persons with regard to the processing of personal data and on the free movement of such data, and repealing Directive 95/46/EC (General Data Protection Regulation). *Official Journal of the European Union*, L119, 1. <https://eur-lex.europa.eu/legal-content/EN/TXT/PDF/?uri=CELEX:32016R0679>

Haywood, C. (2022). Sex clubs in the UK: Recreational sex, erotic diversity and Geographies of desire. *International Journal of the Sociology of Leisure, 5*(3), 297–320. <https://doi.org/10.1007/s41978-022-00108-8>

Hennelly, S. (2010). Public space, Public Morality: The media construction of sex in public places. *The Liverpool Law Review, 31*(1), 69–91. <https://doi.org/10.1007/s10991-010-9073-x>

Joyclub. (n.d.-a). *Impressum* [Imprint]. JoyCLUB. Retrieved October 15, 2023, from <https://www.joyclub.de/impressum/>

Joyclub. (n.d.-b). *Members*. Retrieved October 15, 2023, from <https://www.joyclub.com/en/member/> Accessible after free registration.

Kleinkopf, F. L. (2022). *Text- und Data-Mining* [Text and data mining]. Nomos. <https://doi.org/10.5771/9783748935360>

Mandon, S. (2018, May). *7. Sonderfall: Text und Data Mining* [7. Special case: Text and data mining] (Universität Bremen, Ed.). Retrieved October 27, 2023, from <https://www.uni-bremen.de/urheberrecht/wissensplattform/7-sonderfall-text-und-data-mining>

Mayer, C. (2013). Ehebruch als Dienstvergehen? [Adultery as a service offence?]. *JuristenZeitung, 68*(7), 350–354. <https://www.jstor.org/stable/23467599>

Navarro, P. P. (2017). Beyond inclusion: Non-monogamies and the borders of citizenship. *Sexuality and Culture, 21*(2), 441–458. <https://doi.org/10.1007/s12119-016-9398-2>

Ponath, N. (2022, February 19). *». . .sie wurden gewahr, dass sie alle nackt sind«* [". . .they became aware that they were all naked"]. DER SPIEGEL. Retrieved October 9, 2023, from <https://www.spiegel.de/partnerschaft/joyclub-wie-ein-erotikportal-zum-groessten-digitalen-swingerclub-wurde-a-48f1c59c-7033-4f15-a180-c6dd15f00c69>

Rheinland-Pfalz. (2021, January 12). *Nachbarklage gegen Swingerclub in Koblenz erfolglos* [Neighboring action against swinger club in Koblenz unsuccessful]. Retrieved October 15, 2023, from [https://ovg.justiz.rlp.de//presse-aktuelles/pressemitteilungen/detail/nachbarklage-gegen-swingerclub-in-koblenz-erfolglos](https://ovg.justiz.rlp.de/presse-aktuelles/pressemitteilungen/detail/nachbarklage-gegen-swingerclub-in-koblenz-erfolglos)

Rocher, L., Hendrickx, J. M., & De Montjoye, Y. (2019). Estimating the success of re-identifications in incomplete datasets using generative models. *Nature Communications, 10*(1). <https://doi.org/10.1038/s41467-019-10933-3>

Selig, F. (2023, June 5). *Swingerclub: Chef mit 79, weil ihm sein Arzt dazu riet | Regional* [Swinger club: Boss at 79 because his doctor advised him to do so | Regional]. bild.de. Retrieved October 9, 2023, from  <https://www.bild.de/regional/chemnitz/chemnitz-news/swingerclub-chef-mit-79-weil-ihm-sein-arzt-dazu-riet-84174602.bild.html>

Stühler, H.-U. (2006). Swinger-Clubs in baurechtlicher Sicht [Swinger clubs from the point of view of zoning law]. *Gewerbearchiv 52*(1), 20–26.

Süddeutsche Zeitung. (2012, October 13). *Sex als Konsum* [Sex as consumption]. Retrieved October 9, 2023, from <https://www.sueddeutsche.de/leben/boom-von-swinger-clubs-bei-jungen-gaesten-alle-mal-anfassen-1.1386420-3>

Sweeney, L. (2000). *Simple demographics often identify people uniquely* (Data Privacy Working Paper 3). dataprivacylab.org; Carnegie Mellon University. Retrieved October 27, 2023, from <https://dataprivacylab.org/projects/identifiability/paper1.pdf>

Verkehrsgesellschaft Ennepe-Ruhr mbH & Verkehrsverbund Rhein-Ruhr AöR (Eds.). (2020, December). *Bus network map of the Ennepe-Ruhr district*. Retrieved October 9, 2023, from  <https://www.vrr.de/fileadmin/user_upload/pdf/Stadtlinienplaene/Ennepe-Ruhr-Kreis_rs_2021-compressed.pdf>

Witte, C. (2023, February 26). *Witten: BILD im ältesten Swingerclub von NRW | Regional* [Witten: BILD in the oldest swingers club of North Rhine-Westphalia | Regional]. bild.de. <https://www.bild.de/bild-plus/regional/ruhrgebiet/ruhrgebiet-aktuell/witten-bild-im-aeltesten-swingerclub-von-nrw-83012484.bild.html>

# H. References Related to Sources of Sociodemographic County Data

## Overview

- *Name of the county, population of the county, density of the county*:
  Statistisches Bundesamt (2022b).
- *Average income of the county*:
  Statistisches Bundesamt (2022a).
- *Proportion of inhabitants with a migration background*:
  Statistische Ämter des Bundes und der Länder (2023a), except:
- *Berlin*: Amt für Statistik Berlin-Brandenburg [statistik Berlin Brandenburg] (2023).
- *Hamburg*: Statistisches Amt für Hamburg und Schleswig-Holstein (2023).
- *Köln [Cologne]*: Stadt Köln (2021, p. 14).
- *Leipzig*: Stadt Leipzig (2022).
- *Lüchow-Dannenberg*: Landesamt für Statistik Niedersachsen (2020).
- *München [Munich]*: Landeshauptstadt München [Kreisverwaltungsreferat] (2023).
- *Remscheid*: Stadt Remscheid (2021).
- *Ulm*: Häusser (2020).
- *Proportion of inhabitants in certain age strata*:
  Statistisches Bundesamt (2023c).
- *Number of divorces in the county*:
  Statistische Ämter des Bundes und der Länder (2023b).
- *Share of employees in the production, human services, merchant services, MINT sector and others*:
  Arbeitskreis „Erwerbstätigenrechnung der Länder“ im Auftrag der Statistischen Ämter der Länder [Statistische Ämter der Länder] (2022).

## References Related to Data Sources

Amt für Statistik Berlin-Brandenburg [statistik Berlin Brandenburg]. (2023). *Statistischer Bericht:  A I 5 – hj 1 / 23 - Einwohnerregisterstatistik Berlin 30. Juni 2023 - Bestand - Grunddaten* [Population register statistics Berlin 30 June 2023 - Population - Basic data]. Retrieved September 18, 2023, from <https://download.statistik-berlin-brandenburg.de/1ed2bda91b255dd1/9efa16b6afd4/SB_A01-05-00_2023h01_BE.pdf>

Arbeitskreis „Erwerbstätigenrechnung der Länder“ im Auftrag der Statistischen Ämter der Länder [Statistische Ämter der Länder]. (2022, December). *Erwerbstätigenrechnung*: *Reihe 2 Band 1* [Employment accounts: Series 2 Volume 1] (Hessisches Statistisches Landesamt, Ed.). [statistikportal.de](http://statistikportal.de/). Retrieved September 19, 2023, from <https://www.statistikportal.de/sites/default/files/2022-12/ETR_R2B1_2021_0.pdf>

Häusser, T. (2020, September). *Bevölkerungsvorausrechnung Stadt Ulm 2040*: *Ergebnisbericht* [Population forecast for the city of Ulm in 2040: Results report] (Stadt Ulm, Ed.). Statistik BW. Retrieved September 17, 2023, from <https://www.statistik-bw.de/FaFo/Management/DKB421_BevVorausRechn.pdf>

Landesamt für Statistik Niedersachsen. (2020). *Bevölkerung nach Migrationsstatus 2019 nach Landkreisen und kreisfreien Städten* [Population by migration status in 2019 according to counties and independent cities] [Dataset]. <https://www.statistik.niedersachsen.de/download/159912>

Landeshauptstadt München [Kreisverwaltungsreferat]. (2023). *Bevölkerung am 31.12.2022 nach Alter, Geschlecht und Migrationshintergrund* [Population as of 31.12.2022 by age, gender and migration background] (Statistisches Amt München, Ed.). Retrieved September 17, 2023, from <https://stadt.muenchen.de/dam/jcr:ab9fa157-c362-4a23-90fd-a79f6d0a92fa/jt230103.pdf>

Stadt Köln. (2021). Nationalitäten in Köln: Entwicklung und Status Quo der Immigration [Nationalities in Cologne: Development and status quo of immigration]. *Kölner Statistische Nachrichten, 2021*(10). <https://www.stadt-koeln.de/mediaasset/content/pdf15/statistik-einwohner-und-haushalte/ksn_10_2021_nationalitäten_in_köln.pdf>

Stadt Leipzig. (2022). *Migrantinnen und Migranten in Leipzig 2022* [Migrants in Leipzig 2022]. [leipzig.de](http://leipzig.de/). Retrieved September 19, 2023, from <https://static.leipzig.de/fileadmin/mediendatenbank/leipzig-de/Stadt/02.1_Dez1_Allgemeine_Verwaltung/18_Ref_Migration_und_Integration/Statistik/Faltblatt_Migrantinnen_und_Migranten_in_Leipzig_2022.pdf>

Stadt Remscheid. (2021, June). *Remscheider Einwohnerinnen und Einwohner am 31. März 2021* [Residents of Remscheid on 31 March 2021]. [remscheid.de](http://remscheid.de/). Retrieved September 16, 2023, from <https://www.remscheid.de/neuigkeiten-wissenswertes/stadtportrait/download-pool/3.32-Remscheider-Einwohner-am-31.03.2021.pdf>

Statistische Ämter des Bundes und der Länder. (2023a). *Bevölkerung am Hauptwohnort nach Migrationsstatus - Jahr - regionale Tiefe: Kreise und kreisfreie Städte* [Population at main place of residence by migration status - year - regional depth: counties and county-level cities] [Dataset; 12211-Z-04]. [https://www.regionalstatistik.de/genesis//online?operation=table&code=12211-Z-04&bypass=true&levelindex=1&levelid=1697464736083#abreadcrumb](https://www.regionalstatistik.de/genesis/online?operation=table&code=12211-Z-04&bypass=true&levelindex=1&levelid=1697464736083#abreadcrumb)

Statistische Ämter des Bundes und der Länder. (2023b). *Ehescheidungen - Jahr - regionale Tiefe: Kreise und krfr. Städte :  Statistik rechtskräftiger Urteile in Ehesachen* [Divorces - Year - Regional depth: Counties and cities : Statistics of final judgments in marital matters] (20.09.2023 / 18:48:00) [Dataset; 12631-01-02-4]. [https://www.regionalstatistik.de/genesis//online?operation=table&code=12631-01-02-4&bypass=true&levelindex=0&levelid=1698364569391#abreadcrumb](https://www.regionalstatistik.de/genesis/online?operation=table&code=12631-01-02-4&bypass=true&levelindex=0&levelid=1698364569391#abreadcrumb)

Statistisches Amt für Hamburg und Schleswig-Holstein. (2023, September 4). *Bevölkerung mit Migrationshintergrund in den Hamburger Stadtteilen 2022* [Population with migration background in Hamburg’s districts 2022]: *Anteil der Menschen mit Migrationshintergrund gestiegen – Unterschiede zwischen den Stadtteilen* [Proportion of people with a migration background increased - differences between the city districts]. [statistik-nord.de](http://statistik-nord.de/). Retrieved September 17, 2023, from <https://www.statistik-nord.de/fileadmin/Dokumente/Presseinformationen/SI23_109.pdf>

Statistisches Bundesamt. (2022a). *Haushalte und Familien - Ergebnisse des Mikrozensus - Fachserie 1 Reihe 3 - 2021 (Erstergebnisse) - (Letzte Ausgabe - berichtweise eingestellt)* [Households and families - Results of the microcensus - Subject series 1, part 3 - 2021 (first results) - (latest edition - discontinued on a report-by-report basis)] (No. 2010300217004). Retrieved October 20, 2023, from <https://www.destatis.de/DE/Themen/Gesellschaft-Umwelt/Bevoelkerung/Haushalte-Familien/Publikationen/Downloads-Haushalte/haushalte-familien-2010300217004.pdf>

Statistisches Bundesamt. (2022b). *Daten aus dem Gemeindeverzeichnis: Kreisfreie Städte und Landkreise nach Fläche, Bevölkerung und Bevölkerungsdichte: Gebietsstand: 31.12.2021* [Data from the directory of municipalities: Cities and rural districts by area, population and population density: As of: 31.12.2021] (Herausgebergemeinschaft Statistische Ämter des Bundes und der Länder, Ed.) [Dataset]. <https://www.destatis.de/DE/Themen/Laender-Regionen/Regionales/Gemeindeverzeichnis/Administrativ/Archiv/Standardtabellen/04_KreiseVorjahr.xlsx?__blob=publicationFile>

Statistisches Bundesamt. (2022c). *Bevölkerung: Kreise, Stichtag, Altersgruppen: Fortschreibung des Bevölkerungsstandes* [Population: counties, reference date, age groups: Update of the population level] (30.06.2023 / 18:10:28) [Dataset; 12411-0017]. <https://www-genesis.destatis.de/genesis/downloads/00/tables/12411-0017_00.csv>

1. Although the correlation is statistically significant at p < .05, the bootstrapped 95% confidence interval includes zero, indicating that the correlation may not be robust. This suggests that any observed correlation should be interpreted with caution. [↑](#footnote-ref-2)
2. See footnote 1. [↑](#footnote-ref-3)
3. See footnote 1. [↑](#footnote-ref-4)
4. BB – Brandenburg; BE – Berlin; BY – Bavaria; HE – Hesse; HH – Hamburg; NI – Lower Saxony; NW – North Rhine-Westphalia; SH – Schleswig-Holstein; SL – Saarland; SN – Saxony. Names of cities and towns are used according to the German official spelling. [↑](#footnote-ref-5)
5. The clubs do usually not define the term “couple.” Where single men are not admitted, it is clear from the context that also male-male couples would not be admitted. [↑](#footnote-ref-6)
6. (0) Ordinary sex-oriented event; (1) Event focusing on food, drink, eventually dance; the mere announcement of catering, a music style played, or a DJ are not sufficient; (2) Event providing specific sex-oriented programs, like play games, or strongly focusing on preferences (BDSM, skin complexion, „molly“ (chubby)); not sufficient is a specific dress code or the mere mentioning that active participation or the swapping of partners are appreciated; (3) Parties focusing on both 2 and 3. [↑](#footnote-ref-7)
7. In Euro, rounded to the next full Euro. Based on the assumption of a pre-registration with a free and validated Joyclub profile, and the use of a prepayment option, if available. Specific prices for women are not considered for TS/TG persons who do not present a government ID that states their female gender. If, in case of doubt, TS/TG persons can demonstrate that they are considered female by state institutions, they are treated as female also in terms of entrance fees. The reverse constellation is irrelevant in practice, as, without exception, women pay lower prices than men for the events discussed here. [↑](#footnote-ref-8)
8. Prices for people (men, women, or couples with couple profile) who have a paid premium membership at Joyclub. The events can be advertised in Joyclub free of charge. The management of registrations, including the use of convenience functions, is also free of charge for event organizers. Nevertheless, preferential listing occurs when an event organizer grants a discount to Joyclub premium members, the minimum amount of which is stipulated by Joyclub. See Joyclub (n.d.-a & n.d.-b). [↑](#footnote-ref-9)
9. The venue offers prices with all-inclusive drinks, and another pricing where the drinks have to be paid for extra. In order to allow better comparisons, the drinks-inclusive prices were taken into consideration here. The non-inclusive prices provide for Joyclub premium member discounts. [↑](#footnote-ref-10)
10. *Schlager* is a term for mostly German-language, simple, danceable light music characterized by catchy melodies and straightforward instrumentation. The lyrics typically convey simple messages on topics such as love and justice, or, in party contexts, may feature humorous, silly, parodic, or supposedly taboo-breaking content. See Schlager Radio (2022) and Zweites Deutsches Fernsehen (2023) for examples of supposedly taboo-breaking explicit content in a Schlager song. [↑](#footnote-ref-11)
11. *Discofox*, precisely, is not a music genre but a social partner dance. According to the IDO (2023, p. 90), it “is known and named as Disco Hustle, Swing Fox, Disco Swing, Rock Fox in different regions.” [↑](#footnote-ref-12)
12. A price for solo women is mentioned on the website, but only couples were registered. [↑](#footnote-ref-13)
13. With respect to a definition, see the footnotes to event no 10. [↑](#footnote-ref-14)
14. See Footnote to event number 4. [↑](#footnote-ref-15)
15. This reflects an explicit statement in the description. The author’s distance from the concept of this statement is expressly noted here. [↑](#footnote-ref-16)
16. The author is aware of the sensitivity of this issue. [↑](#footnote-ref-17)
